# Atrioventricular node dysfunction in pressure overload-induced heart failure—Involvement of the immune system and transcriptomic remodelling

**DOI:** 10.3389/fphar.2023.1083910

**Published:** 2023-04-04

**Authors:** Claire Wilson, Min Zi, Matthew Smith, Munir Hussain, Alicia D’Souza, Halina Dobrzynski, Mark R. Boyett

**Affiliations:** ^1^ Division of Cardiovascular Sciences, University of Manchester, Manchester, United Kingdom; ^2^ Institute of Systems, Molecular and Integrative Biology, University of Liverpool, Liverpool, United Kingdom; ^3^ Faculty of Life Sciences, University of Bradford, Bradford, United Kingdom; ^4^ Department of Anatomy, Jagiellonian University Medical College, Kraków, Poland

**Keywords:** heart failure, atrioventricular node, heart block, RNA sequencing, transcriptome, ion channels, inflammation

## Abstract

Heart failure is associated with atrioventricular (AV) node dysfunction, and AV node dysfunction in the setting of heart failure is associated with an increased risk of mortality and heart failure hospitalisation. This study aims to understand the causes of AV node dysfunction in heart failure by studying changes in the whole nodal transcriptome. The mouse transverse aortic constriction model of pressure overload-induced heart failure was studied; functional changes were assessed using electrocardiography and echocardiography and the transcriptome of the AV node was quantified using RNAseq. Heart failure was associated with a significant increase in the PR interval, indicating a slowing of AV node conduction and AV node dysfunction, and significant changes in 3,077 transcripts (5.6% of the transcriptome). Many systems were affected: transcripts supporting AV node conduction were downregulated and there were changes in transcripts identified by GWAS as determinants of the PR interval. In addition, there was evidence of remodelling of the sarcomere, a shift from fatty acid to glucose metabolism, remodelling of the extracellular matrix, and remodelling of the transcription and translation machinery. There was evidence of the causes of this widespread remodelling of the AV node: evidence of dysregulation of multiple intracellular signalling pathways, dysregulation of 109 protein kinases and 148 transcription factors, and an immune response with a proliferation of neutrophils, monocytes, macrophages and B lymphocytes and a dysregulation of 40 cytokines. In conclusion, inflammation and a widespread transcriptional remodelling of the AV node underlies AV node dysfunction in heart failure.

## Introduction

Heart failure (HF) is associated with atrioventricular (AV) node dysfunction. First-degree AV block is present in 15%–51% of HF patients as compared to a prevalence of 2.1%–4% in the general population (Aro et al., 2014; Nikolaidou et al., 2016). First-degree AV block in HF is associated with an increased risk of mortality and HF hospitalisation, and optimisation of AV delay in patients by means of cardiac resynchronisation therapy is an important treatment (Nikolaidou et al., 2016). AV node dysfunction has also been widely observed in animal models of HF (e.g., Nikolaidou et al., 2015). The aim of this study was to investigate the causes of the AV node dysfunction by studying all changes occurring in the AV node in HF. For this, we used RNAseq to study the transcriptome of the AV node. The aetiology of HF in patients is likely to be mixed and complicated (Roger, 2021). Nevertheless, hypertension is the commonest cause of HF (Bui et al., 2011) and, although it is not a model of hypertension *per se* (Riehle and Bauersachs, 2019), we studied a mouse model of pressure overload-induced HF: the transverse aortic constriction model (Richards et al., 2019). Widespread changes were observed (in 3,077 transcripts), showing that AV node dysfunction is the result of the failure of multiple cellular systems.

## Materials and methods

Male eight-week-old C57Bl/6N mice underwent transverse aortic constriction (TAC) or sham operations. Mice were terminated 8 weeks post-surgery or when HF symptoms developed. In the conscious mouse, the heart rate was measured weekly following surgery using an ECGenie. Prior to termination, in anaesthetised mice, echocardiography was carried out to determine heart function, and the electrocardiogram (ECG) was recorded to determine the PR interval, etc*.* AV node biopsies were collected from the triangle of Koch bordered by the coronary sinus, tendon of Todaro and tricuspid valve annulus (Li et al., 2008); the base, height and area of the triangle was ∼0.5 mm, ∼0.75 mm and 0.19 mm^2^, respectively. AV node biospies taken from three mice were pooled and three such replicates were obtained from HF (TAC) and control (sham-operated) mice. RNA was extracted and sequenced (RNAseq) to yield the expression level of 55,385 transcripts. Statistical analysis was carried out to determine significant differences in transcript expression, and gene ontology and pathway analysis was carried out to understand the functional significance of the data. All experimental procedures were approved by the University of Manchester and were in accordance with the United Kingdom Animals (Scientific Procedures) Act 1986. Further details of the methods used are given in the Supplementary Materials and Methods.

## Results

### TAC mouse model of pressure overload-induced HF and AV node dysfunction

**FIGURE 1:**
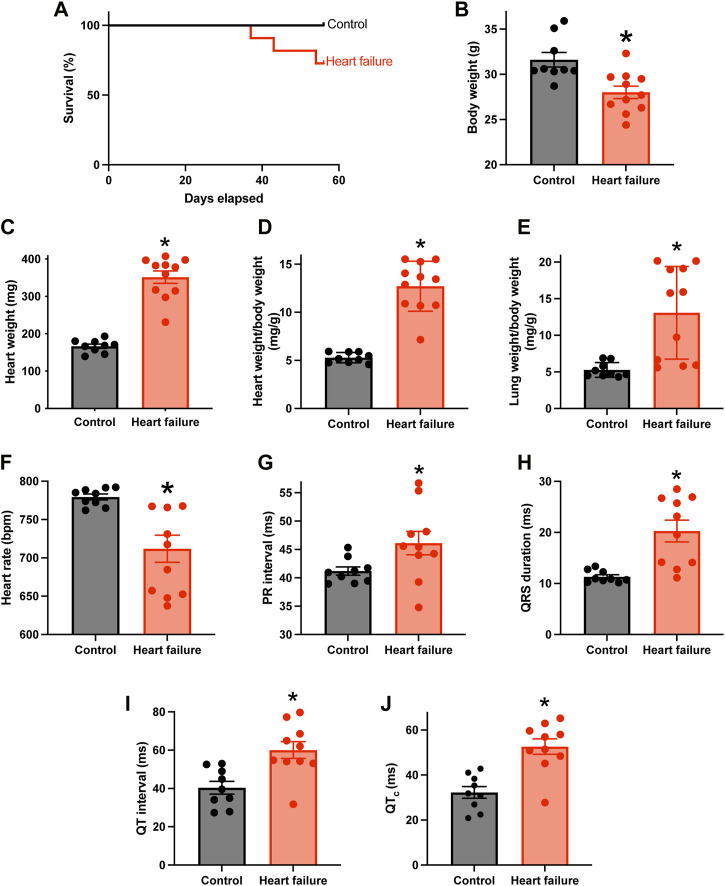
HF model with AV node dysfunction. **(A)**, percentage survival of control (*n* = 9; subject to sham surgery) and HF (*n* = 11; subject to TAC surgery) mice in the days following surgery. **(B–F)**, body weight **(B)**, heart weight **(C)**, heart weight:body weight ratio **(D)**, lung weight:body weight ratio **(E)** and *in vivo* heart rate (**(F)**; measured using the ECGenie) of the two cohorts of mice (*n* = 9 and 11) at the end of the experiment. **(G–J)**, PR interval **(G)**, QRS duration **(H)**, QT interval **(I)** and corrected QT interval **(J)** of the two cohorts of mice (*n* = 9 and 10) measured in the anaesthetised animals at the end of the experiment. In B-J, means ± SEM as well as individual data points shown. *p < 0.05.

Figure 1A shows that from day 36 following TAC surgery, some mice reached prescribed endpoints and had to be culled, whereas none of the control sham-operated mice had to be culled. Figures 1B–D shows that by the end of the experiment, in the case of the mice subjected to TAC surgery, the body weight was significantly reduced, and the heart weight and heart weight:body weight ratio were significantly increased, indicative of HF. The relative loss of body weight (as compared to the body weight of the control mice) occurred progressively after the TAC surgery (Supplementary Figure S1A). Following TAC surgery, the mice developed congestive HF—the lung weight:body weight ratio was significantly increased in the mice subjected to TAC surgery by the end of the experiment (Figure 1E). Echocardiography at the end of the experiment confirmed HF: fractional shortening and ejection fraction were significantly reduced and left ventricular mass and end diastolic and systolic diameters were significantly increased (Supplementary Figure S1B–F). HF was accompanied by electrophysiological changes: the ECG was recorded in the anaesthetised mouse at the end of the experiment, and in the HF mice there was a significant increase in the PR interval indicative of AV node dysfunction (a slowing of AV node conduction) (Figure 1G). There was also a significant decrease in the heart rate and significant increases in the QRS duration and uncorrected and corrected QT intervals in the HF mice as expected (Figures 1F,H–J). See the Supplementary Material for further discussion of the data as well as justification of the mouse TAC model as a model of HF. From this point onwards, pressure overload-induced HF will be simply referred to as HF.

### Transcriptomic remodelling of the AV node

**FIGURE 2:**
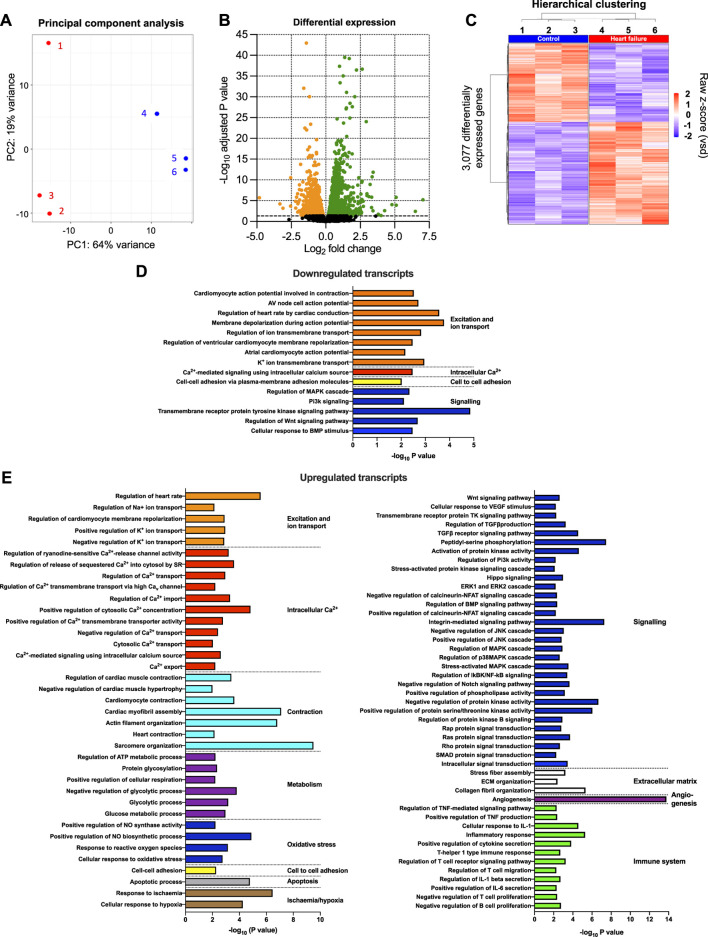
Transcriptomic remodelling of the AV node in HF. **(A)**, principal component analysis (PCA) of all six replicates based on the 500 most highly expressed transcripts. The *x*-axis shows the PCA 1 score, which represents the maximum variance direction in the data set. This is principal component 1 (PC1) and it accounts for 64% of the variance in the samples and can be attributed to the variance between the control and HF cohorts. The *y*-axis shows the PCA 2 score, which represents the second highest variance direction in the data set. This is principal component 2 (PC2) and it accounts for 19% of the variance in the samples and can be attributed to the variance among all samples. The red symbols (1, 2 and 3) represent the three HF technical replicates and the blue symbols (4, 5 and 6) represent the three control technical replicates. Each technical replicate contained three pooled AV node biopsies. **(B)**, volcano plot showing -log_10_ adjusted *p*-value plotted against log_2_ fold change for each transcript measured. Dashed line corresponds to adjusted *p* = 0.05. The red and blue symbols show the 3,077 transcripts significantly upregulated (red symbols; 1,740 transcripts) or downregulated (blue symbols; 1,337 transcripts). **(C)**, hierarchical clustering of the 3,077 differentially expressed transcripts (adjusted *p* < 0.05) performed using Pearson’s correlation distance and ward. D2 agglomeration method. Data for the six samples shown. Expression (raw z-score) goes from high (red) to low (blue). **(D and E)**, functional enrichment analysis performed using the topGO R package on downregulated **(D)** and upregulated **(E)** differentially expressed genes in the HF samples compared to the control samples. Significantly enriched biological process GO terms related to cardiac function are presented.

The transcriptome of the AV node was determined using RNAseq, and to have sufficient material, AV node tissue isolated from three mice was pooled. Three biological replicates (each a pool from three mice) were isolated from each cohort of mice (the study therefore involved nine HF and nine control mice). 55,385 transcripts were detected and their abundance quantified. Principal component analysis (PCA) for each of the three technical replicates in the transcriptomics dataset showed clear distinction of the samples into two groups corresponding to the HF and control cohorts of mice (Figure 2A). 64% of the variation between samples (PC1) was attributed to HF, whereas 19% of the variation (PC2) was attributed to variation between technical replicates (Figure 2A). Figure 2B shows a volcano plot of the Benjamini-Hochberg-adjusted *p*-value against the fold change for all transcripts. The dashed line corresponds to *p* = 0.05, the threshold for significance. There were significant changes in the abundance of 3,077 transcripts (5.6% of the total). Unsupervised hierarchical clustering of the transcripts changing significantly in HF resulted in a grouping of the HF and control cohorts of mice into two distinct clusters (Figure 2C). 1,337 transcripts were significantly downregulated and 1,740 transcripts including well-known HF markers were significantly upregulated in HF (Figures 2B,C; Supplmentary Figure S2). A gene ontology analysis was carried out of the transcripts significantly downregulated or upregulated in HF using topGO and this showed that HF had widespread effects on the cellular systems of the AV node. In Figures 2D,E the most significant of these pathways is shown (*p* < 0.01). The set of transcripts downregulated in HF is enriched for transcripts related to “excitation and ion transport” for example, (Figure 2D), whereas the set of transcripts upregulated in HF is enriched for transcripts related to “intracellular Ca^2+^,” “contraction,” “metabolism,” “apoptosis,” “signalling,” “extracellular matrix” and the “immune system” for example, (Figure 2E). The effects of HF on some of these systems is now considered in detail.

### Downregulation of transcripts in the AV node expected to impact conduction

**FIGURE 3:**
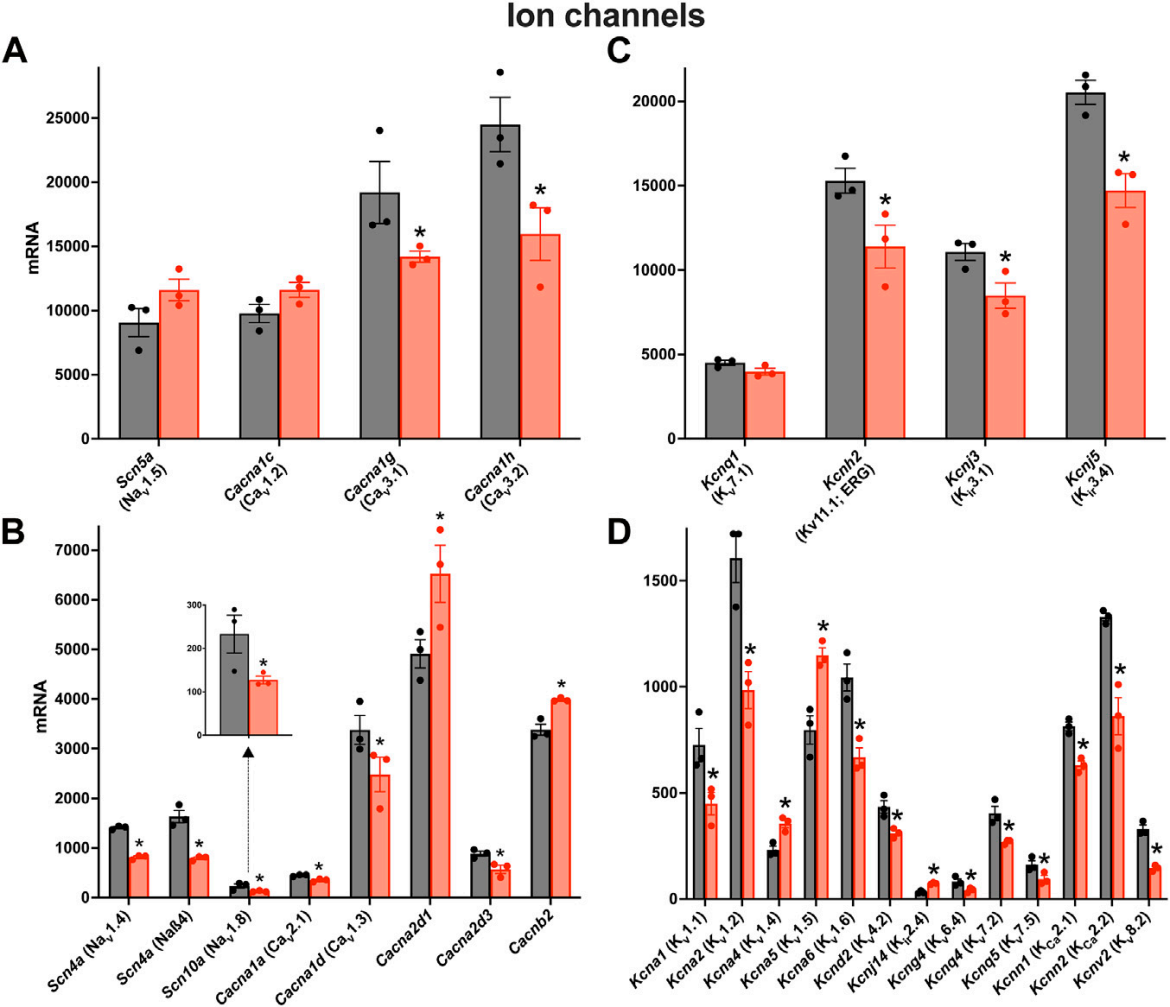
Downregulation of many key ion channel transcripts in the AV node in HF. **(A–D)**, expression of transcripts for Na^+^ and Ca^2+^ channels **(A and B)** and K^+^ channels **(C and D)** in control (black bars) and HF (red bars). Highly expressed ion channel transcripts are shown in the upper panels **(A and C)** and more poorly expressed ion channel transcripts are shown in the lower panels **(B and D)**. The data for *Scn10a* are also shown in a zoomed panel. Gene names and more common names are shown. Means ± SEM as well as individual data points shown *p < 0.05.

There was a remodelling of ion channel transcripts in the AV node in HF, some of which are expected to impact AV node conduction. Whereas *Scn5a* responsible for the principal Na^+^ channel in the heart, Nav1.5, was unaffected (Figure 3A), other less abundant Na^+^ channel transcripts were significantly downregulated (Figure 3B). *Scn10a* (responsible for Nav1.8) was scarce compared to *Scn5a*, but it was significantly downregulated (Figure 3B) and this may be important: GWAS studies have implicated *Scn10a* as a modulator of cardiac conduction (Park and Fishman, 2014) including the PR interval (Chambers et al., 2010; Holm et al., 2010; Pfeufer et al., 2010), and mice treated with a selective inhibitor of Nav1.8 channels show a marked prolongation of PR and QRS intervals (Park and Fishman, 2014). The mechanism of action of *Scn10a* is unclear (Park and Fishman, 2014). Three key Ca^2+^ channel transcripts were significantly downregulated: *Cacna1d*, *Cacna1g* and *Cacna1h*, responsible for the L- and T-type Ca^2+^ channels, Cav1.3, Cav3.1 and Cav3.2 (Figures 3A,B). This is likely to be important, because in mice, loss-of-function of Cav1.3 and Cav3.1 impairs AV conduction and in humans AV block has been attributed to loss-of-function of Cav1.3 and Cav3.1 (Mesirca et al., 2015). The Ca^2+^ channel accessory subunit, *Cacna2d3*, was significantly downregulated (Figure 3B) and it too has been linked to cardiac conduction (Auxerre-Plantié et al., 2019). However, *Cacna2d1* was significantly upregulated (Figure 3B) and this is expected to increase the L-type Ca^2+^ current (Templin et al., 2011). Three key abundant K^+^ channel transcripts were significantly downregulated, *Kcnh2*, *Kcnj3* and *Kcnj5* (Figure 3C); *Kcnh2* is responsible for the ERG (Kv11.1) channel, and *Kcnj3* and *Kcnj5* are responsible for Kir3.1 and Kir3.4, which together make up the ACh-activated K^+^ channel. Regan et al. (Regan et al., 2005) observed that selective pharmacological block of ERG (responsible for the K^+^ current, *I*_K,r_) and KvLQT1 (responsible for the K^+^ current, *I*_K,s_) both cause a prolonged AH interval and AV node effective refractory period in rats. Although *Kcnh2* responsible for ERG was downregulated in HF, *Kcnq1* responsible for KvLQT1 was not (Figure 3C). Regardless, this demonstrates that K^+^ channels are able to affect AV node conduction and refractoriness. Mesirca et al. (Mesirca et al., 2016) reported that ablation of the ACh-activated K^+^ current (carried by Kir3.1 and Kir3.4) relieves the AV block in Cav1.3 knockout mice. The downregulation of *Kcnj3* and *Kcnj5* responsible for Kir3.1 and Kir3.4 in HF (Figure 3C) could, therefore, be a compensatory mechanism, although it is also expected to reduce the response of the AV node to vagal stimulation. There were many significant changes of the less abundant K^+^ channel transcripts (Figure 3D). *Kcna1* and *Kcna2* (responsible for Kv1.1 and Kv1.2) were both significantly downregulated in HF (Figure 3D). In the mouse, systemic knockout of *Kcna1* (Kv1.1) results in AV block–although this was attributed to the autonomic nervous system, it could be a direct action on the AV node (Glasscock et al., 2010; Glasscock, 2019). Transgenic mice expressing high levels of activated RhoA (a GTPase implicated in cardiac hypertrophy) develop lethal ventricular failure (Sah et al., 1999). The mice develop AV block and Sah et al, (1999) hypothesised that this was the result of RhoA physically associating with and suppressing the activity of Kv1.2 (Cachero et al., 1998). Interestingly, *RhoA* was significantly upregulated in the AV node in HF in the present study (data not shown). Transcripts for four Ca^2+^-activated K^+^ channels were present and *Kcnn2* (responsible for K_Ca_2.2 or SK2) was the most abundant (Figure 3D). *Kcnn2* and *Kcnn1* (responsible for K_Ca_2.1 or SK1) were significantly downregulated in HF (Figure 3D) and this is likely to be functionally important. In the mouse, deletion of one copy of the *Kcnn2* gene results in AV node dysfunction–a prolongation of the AV node action potential, a slowing of AV node spontaneous activity and an increase in the PR interval–and deletion of both copies results in complete AV block (Zhang et al., 2008).

It is possible that the changes in ion channel expression are dependent on the severity of HF. To explore this, for each of the six pooled samples measured, the expression of transcripts for key ion channel subunits was plotted against *Myh7*, a well-known HF marker, and in all cases with one exception there was a significant correlation (Supplementary Figure S3). However, they were not correlated with *Nppa* and *Nppb*, two other HF markers.

Changes of further ion channels and transporters are considered in the (Supplementary Figures S4–S7).

### Evidence of a shift from fatty acid to glucose metabolism

**FIGURE 4:**
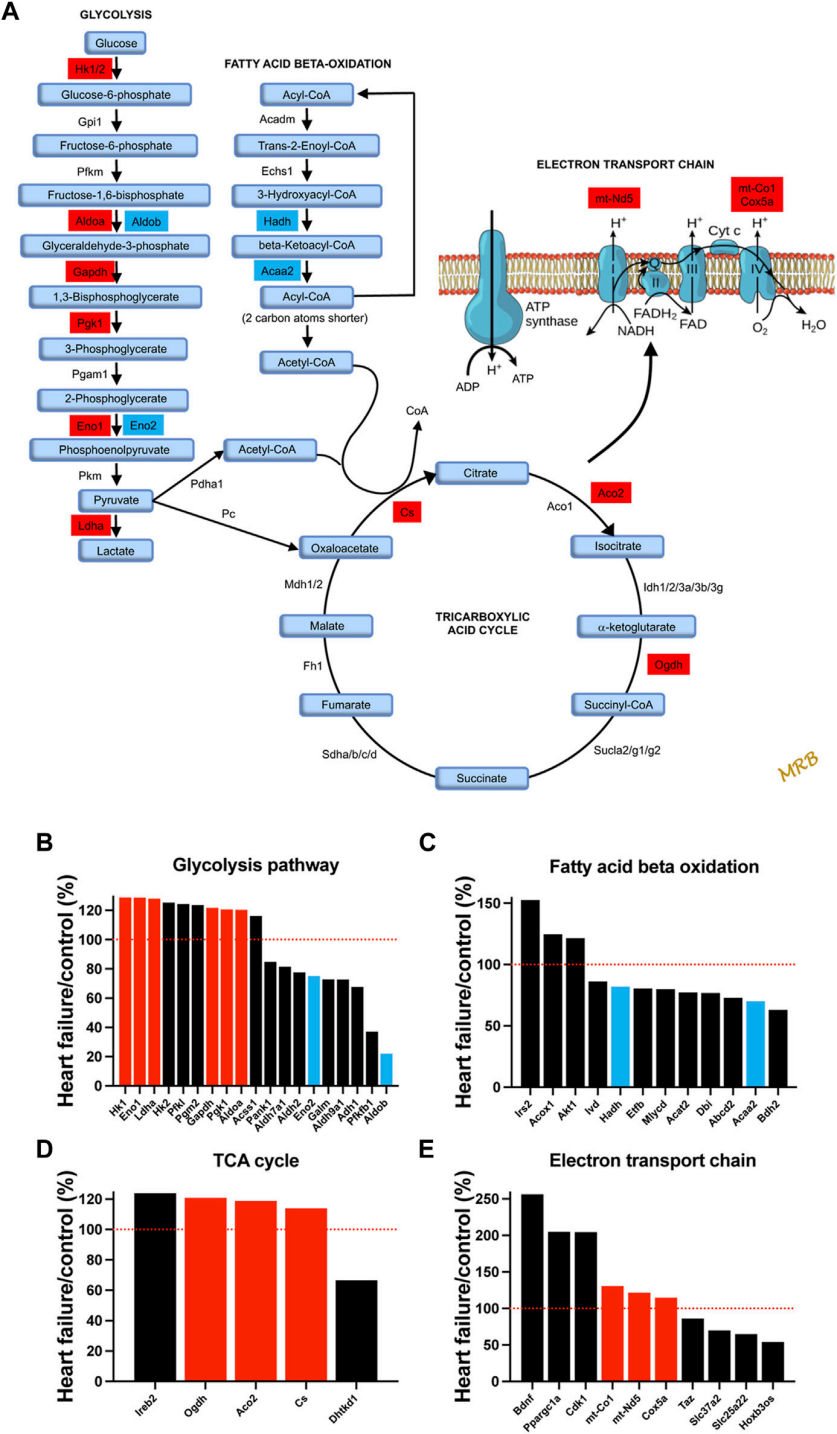
Evidence of a shift from fatty acid to glucose metabolism in the AV node in HF. **(A)**, schematic diagram of glycolysis, fatty acid beta-oxidation, the tricarboxylic acid cycle, and the electron transport chain. For glycolysis, fatty acid beta-oxidation, and the tricarboxylic acid cycle, key enzymes and metabolic intermediaries (in light blue) are shown. Transcripts of enzymes highlighted in red were significantly upregulated in HF and transcripts of enzymes highlighted in dark blue were significantly downregulated. For the electron transport chain, ATP synthase and complexes I to IV are shown. Transcripts associated with complexes I and IV were significantly upregulated and are highlighted in red. **(B–E)**, expression of transcripts associated with glycolysis **(B)**, fatty acid beta-oxidation **(C)**, the tricarboxylic acid cycle **(D)** and the electron transport chain **(E)** in HF mice as a percentage of that in control mice. All transcripts changed significantly in HF. Significantly upregulated transcripts highlighted in **A** are shown in red and significantly downregulated transcripts highlighted in **A** are shown in blue. Red dotted lines correspond to 100%.

It is well known that HF is associated with metabolic remodelling and energy insufficiency (Doenst et al., 2013; Wende et al., 2017). This insufficiency is thought to play an important role in the development of HF. Under normal conditions, the heart derives 50%–70% of the ATP needed from fatty acids with glucose contributing less (Wende et al., 2017). However, under stress conditions, there can be a shift from fatty acid to glucose utilisation (Tran Diem and Wang Zhao, 2019). Glucose generates more ATP than fatty acid for each mole of oxygen (Shiojima and Walsh, 2006). For the first time, we show that a qualitatively similar metabolic remodelling occurs in the AV node. Transcripts which change significantly in HF and are associated with glycolysis, fatty acid beta-oxidation, the tricarboxylic cycle, and the electron transport chain are shown in Figure 4. Glucose entry into cardiac myocytes is facilitated by GLUT1-13 transporters (Kewalramani and Rodrigues, 2009), but there were no changes in the corresponding transcripts (*Slc23a1-13*; Supplementary Figure S8A). Transcripts for six key enzymes in glycolysis were significantly upregulated in HF (Figure 4B) and are highlighted red in the metabolic pathway (Figure 4A). In two cases there appeared to be a partial isoform switch: whereas *Aldoa* and *Eno1* were significantly upregulated in HF, *Aldob* and *Eno2* were significantly downregulated (Figures 4A,B). Fatty acid is transported into cardiac myocytes by FAT/CD36 (*Cd36*), FABPpm (*Got2*) and FATP1-6 (*Slc27a1-6*) transporters (Chabowski et al., 2008); *Cd36* was significantly upregulated and *Slc27a1* was significantly downregulated (Supplementary Figure S8B). Transcripts for two key enyzmes in the fatty acid beta-oxidation pathway (*Hadh* and *Acaa2*) were significantly downregulated in HF (Figure 4C) and are highlighted blue in the metabolic pathway shown in Figure 4A. Although in the same set of transcripts shown in Figure 4C, *Irs2* and *Akt1* were upregulated in HF, AKT enhances glycolysis by promoting glucose uptake and may inhibit fatty acid oxidation (Shiojima and Walsh, 2006), and IRS similarly enhances glycolysis and inhibits fatty acid oxidation by acting *via* AKT (as well as AMP-activated protein kinase) (Long et al., 2011). *Ppargc1a* (PGC-1α) is a master regulator of mitochondrial functions (Austin et al., 2011) and *Ppargc1a* and also *Ppara* are involved in transcriptional regulation of fatty acid oxidation enzymes (Wende et al., 2017); both were upregulated (Supplementary Figure S9A). AMP-activated protein kinase is a central regulator of metabolism and it promotes both glycolysis and fatty acid oxidation (Kewalramani and Rodrigues, 2009), and there was an upregulation of three key AMP-activated protein kinase subunits (Supplementary Figure S9B). Transcripts for three key enzymes in the tricarboxylic acid cycle were significantly upregulated in HF (Figure 4D) and are again highlighted red in the metabolic pathway (Figure 4A). Finally, transcripts for three key enzymes in the electron transport chain were significantly upregulated in HF (Figure 4E) and are highlighted red in the metabolic pathway (Figure 4A); *mt-Nd5* is responsible for NADH dehydrogenase 5, part of complex I, and *mt-Co1* and *Cox5a* are responsible for cytochrome c oxidase subunits I and 5a of complex IV. In addition, *Ppargc1a* (the master regulator of mitochondrial functions) was significantly upregulated (Figure 4E) as already discussed and this has been shown to affect the electron transport chain (Austin et al., 2011).

### Evidence of activation of intracellular signalling pathways and transcription factors

**FIGURE 5:**
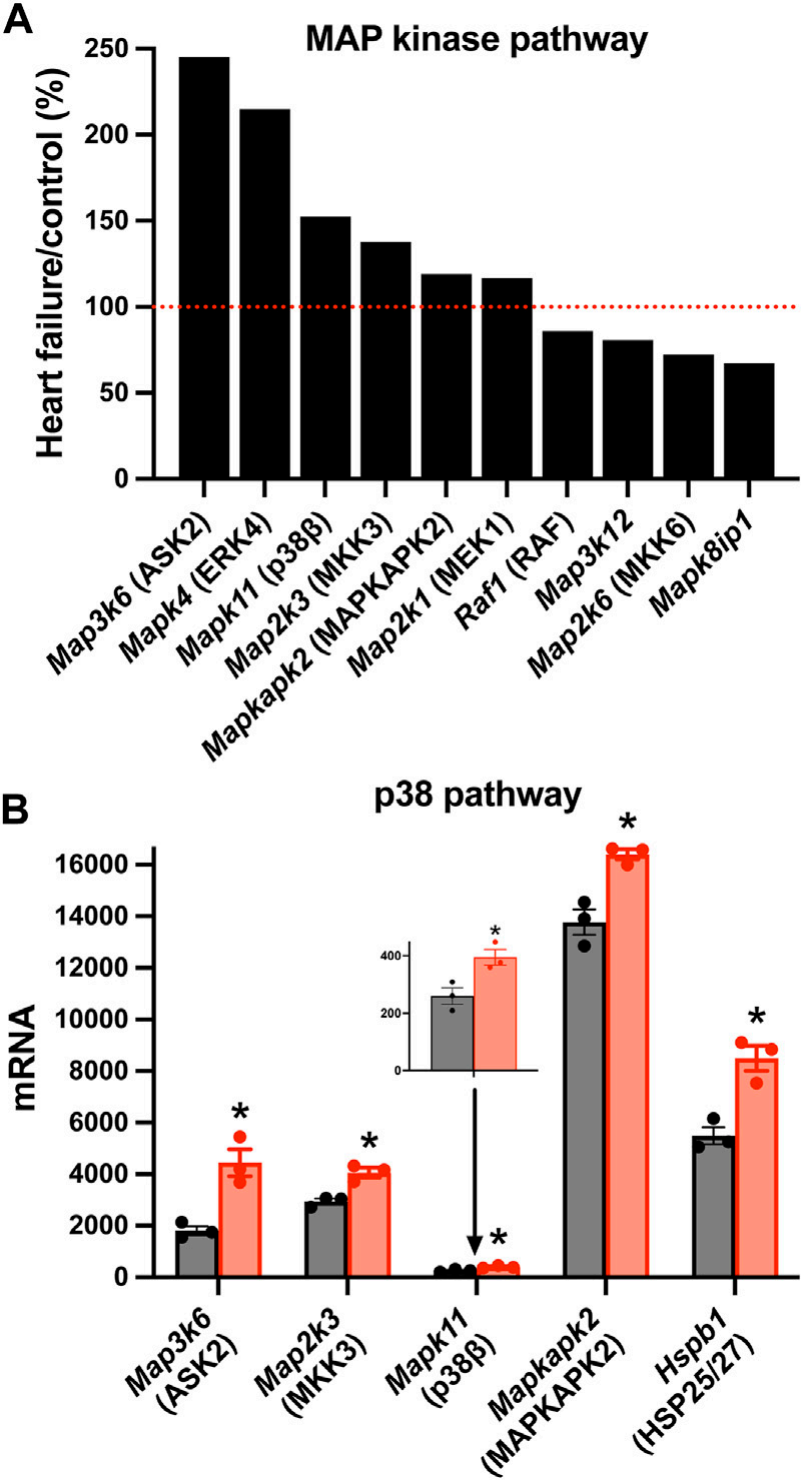
Changes in MAP kinase pathway transcripts in the AV node in HF. **(A)**, significant changes in the expression of MAP kinase pathway transcripts. Expression in HF mice is shown as a percentage of that in control mice. Red dotted line corresponds to 100%. **(B)**, mean (+SEM) expression (as well as individual data points) of p38 pathway transcripts in control (black bars) and HF (red bars) mice. The data for *Mapk11* are also shown in a zoomed in panel. *p < 0.05.

There was a significant upregulation of key transcripts in various intracellular signalling pathways and, as an example, the mitogen-activated protein (MAP) kinase pathway will be considered here. MAP kinases play an important signalling role in the heart (Rose et al., 2010). Various members of the family were significantly affected in the AV node in HF (Figure 5A). In particular, various members of the p38 signalling pathway (one arm of the MAP kinase signalling network) were upregulated (Figure 5B). MAP kinase signalling is prototypically activated by three tiers of phosphorylation: a MAP kinase kinase phosphorylates and activates a MAP kinase, which in turn phosphorylates and activates a MAP kinase (mediator of biological responses) (Rose et al., 2010). In the p38 pathway, *Map3k6* was upregulated (Figure 5); *Map3k6* is responsible for ASK2, which together with ASK1, acts as a MAP kinase kinase (Takeda et al., 2007). *Map3k6* has previously been reported to be upregulated in the heart in the mouse model of pressure overload-induced HF (Matkovich et al., 2012). In the p38 pathway, *Map2k3* was upregulated (Figure 5); *Map2k3* is responsible for MKK3 and acts as a MAP kinase. Finally, in the p38 pathway, *Mapk11* was upregulated (Figure 5); *Mapk11* is responsible for p38β (one of four p38 isoforms present). p38α/β phosphorylates the downstream kinase, MAPKAPK2, which phosphorylates the heat shock protein HSP25/27 and the transcripts for both (*Mapkapk2* and *Hspb1*) were significantly upregulated (Figure 5B). p21-activated kinases (PAKs) are a group of p38 activators and *Pak2* and *Pak3* were upregulated in HF (although *Pak6* was downregulated; data not shown) (Zarubin and Han, 2005). The p38 pathway is activated in HF and the resultant pathological remodelling can lead to cardiac arrhythmias (Romero-Becerra et al., 2020). Interestingly, p38β regulates Hippo signalling (Huang et al., 2016). p38 inhibition improves heart function in pressure-overload right ventricular hypertrophy (Kojonazarov et al., 2017). The activity of MAP kinases are tightly controlled by a family of dual-specificity MAP kinase phosphatases (DUSPs) and they have been shown to have a variety of physiological and pathological roles (Liu and Molkentin, 2016). 10 DUSP transcripts were significantly up or downregulated in HF (data not shown).

There was also a significant upregulation of key transcripts in the protein kinase A, Ca^2+^-calmodulin-dependent protein kinase II (CaMKII), Hippo, WNT, JAK-STAT, and NOTCH signalling pathways (Supplementary Figures S10–S13). Protein phosphorylation is an important signalling mechanism, and it is determined by a balance of protein kinases and phosphatases and there was a remodelling of both protein kinases and phosphatases at the transcript level (Supplementary Figures S14, S15). Transcript expression is primarily governed by transcription factors, and in the mouse Zhou et al. (2017) have identified 941 transcription factors. Of these, transcripts for 927 were identified in the AV node and 148 (16%) changed significantly in HF (Supplementary Figure S16). 81 (55%) were upregulated and 67 (45%) downregulated (Supplementary Figure S16).

### Remodelling of extracellular matrix transcripts

**FIGURE 6:**
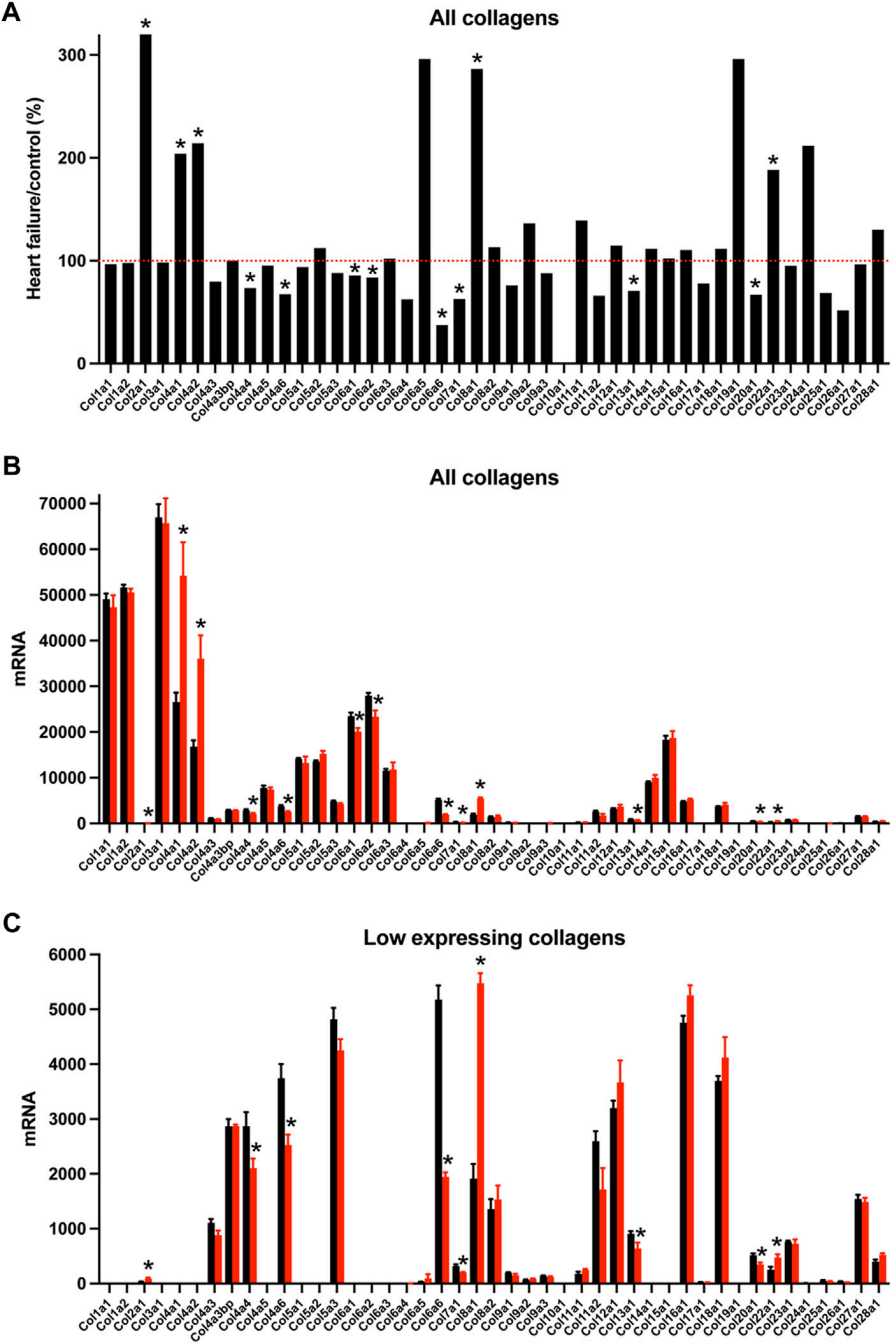
Transcriptomic remodelling of the extracellular matrix of the AV node in HF. **(A)**, expression of all collagen transcripts in HF mice as a percentage of that in control mice. Red dotted line corresponds to 100%. **(B and C)**, expression of highly expressed collagen transcripts **(B)** and more poorly expressed collagen transcripts **(C)** in control (black bars) and HF (red bars) mice. Only means + SEM shown (individual data points not shown for clarity). *p < 0.05.

Nodal dysfunction has frequently been attributed to fibrosis, i.e., a proliferation of the fibrillar collagens (Suarez-Mier et al., 1995; Bailey et al., 2007; Swaminathan et al., 2011; Csepe et al., 2015), an important component of the extracellular matrix. The “Matrisome” from Naba et al, (2016) lists 1,110 transcripts in the mouse known to be associated with the extracellular matrix divided into four different categories. In the AV node in HF, many significant changes in Matrisome transcripts were observed (221 transcripts, i.e. 20% of the total) showing that there was likely a remodelling of the extracellular matrix. Collagens are the most abundant proteins in the extracellular matrix. Transcripts for two fibrillar collagens were present, types 1 and 3; they were the most abundant of the collagens, but they did not change in HF (Figure 6). Basement membranes are dense extracellular matrix structures, and previously we have shown that basement membrane surrounds each sinus node myocyte (Linscheid et al., 2019). Collagen type 4 is a major component of basement membranes (Steffensen and Rasmussen, 2018) and *Col4a1* and *Col4a2*, the two most abundant collagen type 4 α-chain transcripts, were significantly upregulated in the AV node in HF (Figure 6). Collagen type 4 expression is known to be altered in various pathophysiological conditions (Shamhart and Meszaros, 2010). An increase in collagen type 4 may thicken the basement membrane of myocytes and impede extracellular communication with other cells (de Castro Brás et al., 2014). Collagen type 6 α-chain transcripts were significantly downregulated in the AV node in HF (Figure 6). Collagen type 6 carries out cytoprotective functions as well as mechanical roles (Cescon et al., 2015). In part by binding to collagen type 4 in the basement membrane, collagen type 6 links the myocyte to the extracellular matrix. In dilated cardiomyopathy in the human, Gil-Cayuela et al, (2016) reported there is an increase in the transcript (*Col8a1*) and protein for the non-fibrillar collagen eight α1 subunit, and there is a significant relationship between left ventricular mass and *Col8a1* expression. There was also a significant upregulation of *Col8a1* in the AV node in HF (Figure 6). Gil-Cayuela et al, (2016) hypothesise that overexpression of a number of non-fibrillar collagen genes may promote pathological remodelling by inducing apoptosis and hypertrophy of cardiomyocytes. The sum of all collagen transcripts was not significantly altered in HF (data not shown). Changes in other components of the Matrisome are considered in the Supplementary Results (Supplementary Figures S17–S19).

### Evidence of an immune response

**FIGURE 7:**
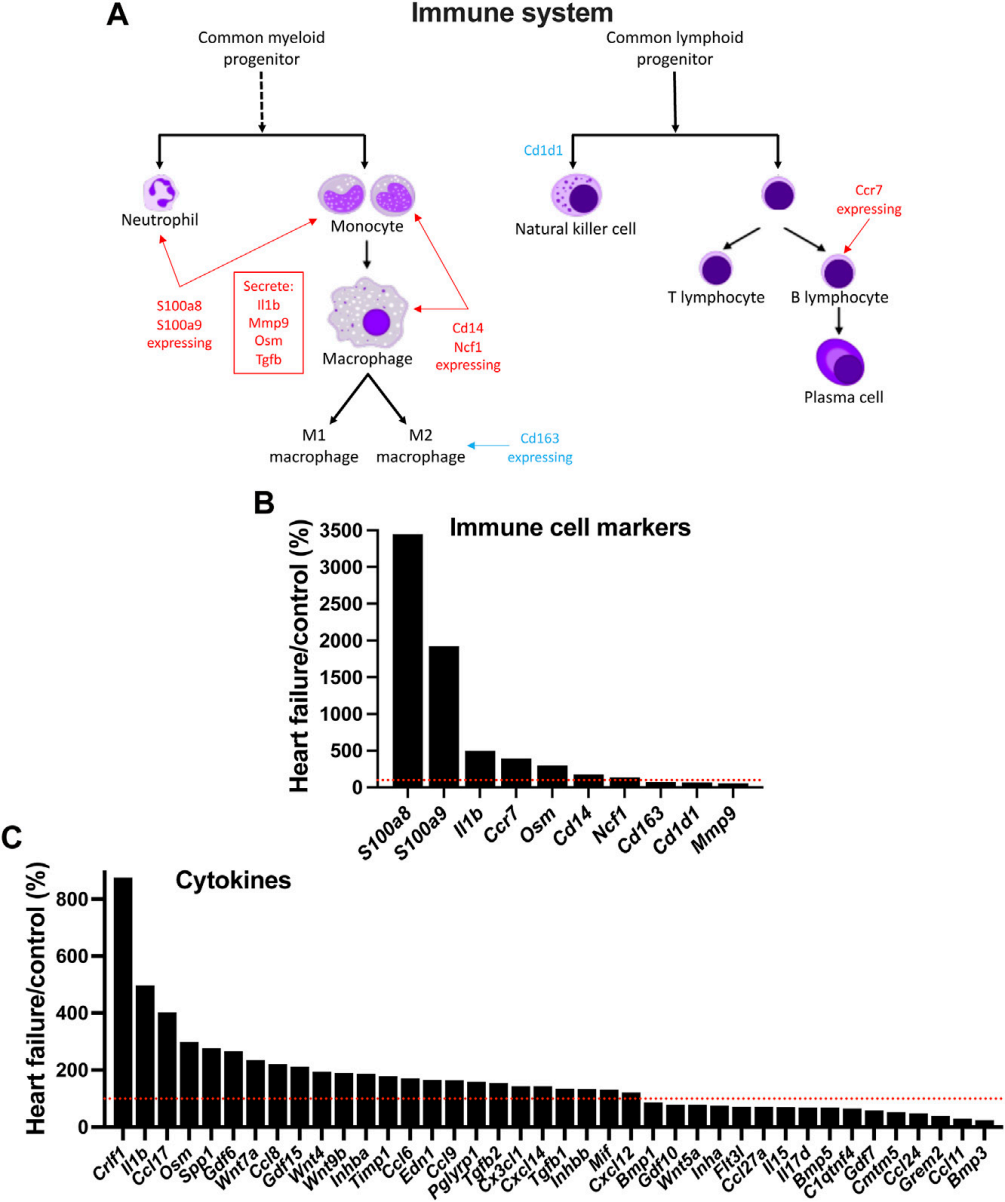
Evidence of infiltration of the AV node by immune cells in HF. **(A)**, schematic diagram of the cells of the immune system. **(B)**, expression of immune cell markers (transcripts) in HF mice as a percentage of that in control mice. **(C)**, expression of cytokine transcripts in HF mice as a percentage of that in control mice. Red dotted lines correspond to 100%.

In HF, although inflammation plays a key role in the development of ventricular dysfunction (Martini et al., 2019; Strassheim et al., 2019), it is not known whether it plays a similar role in AV node dysfunction. Using single cell RNAseq to measure the expression of transcript markers, Martini et al, (2019) showed that the myocardium is infiltrated by a variety of immune cell types in the mouse TAC model of HF. Figures 7A,B shows the expression of various immune cell marker transcripts in the AV node which changed significantly in HF. *S100a8* and *S100a9*, expressed in neutrophils and monocytes, were upregulated (Figures 7A,B). During inflammation, S100A8/A9 is released and modulates the inflammatory response by stimulating leukocyte recruitment and inducing cytokine secretion (Wang et al., 2018). *Cd14* is expressed in monocytes, macrophages, dendritic cells and neutrophils (Marcos et al., 2010) and was upregulated in the AV node in HF (Figures 7A,B). *Ncf1* forms a subunit of NADPH oxidase, which is primarily active in phagocytes (including monocytes and macrophages) and plays an essential role in the immune system; *Ncf1* was upregulated in the AV node in HF (Figures 7A,B). These data suggest that neutrophils, monocytes and macrophages are infiltrating the AV node in HF. However, this may not be true of all macrophages, which exist as M1 cells (pro-inflammatory) or M2 cells (involved with resolution of inflammation and repair of damaged tissues). The data suggest a decline in the M2 cell type: the M2-like marker, *Cd163* (Martini et al., 2019), was downregulated and also the repair associated transcript, *Mmp9* (Martini et al., 2019), was downregulated (Figures 7A,B). *Cd1d1* is involved in natural killer T cell development (Sundararaj et al., 2018) and this too was downregulated (Figures 7A,B). However, phagocytes may not be the only immune cell type to be infiltrating the AV node in HF, because *Ccr7*, a marker of B lymphocytes (Mcheik et al., 2019), was also upregulated (Figures 7A,B). Cytokines are signalling molecules, largely secreted by cells of the immune system (predominantly macrophages and T helper cells), which mediate communication among immune and non-immune cells and are the means by which immune cells may impact the heart. Figure 7C shows that a large number of cytokine transcripts were significantly altered in the AV node in HF, some with known connections to HF; see SupplementaryFigure S20 for further information.

### HF-dependent changes in transcripts identified by GWAS as determinants of the PR interval or associated with hereditary AV node dysfunction

GWAS studies have identified 44 genes associated with an abnormal PR interval (van Setten et al., 2018). Of these, the transcripts associated with 11 genes changed significantly in the AV node in HF (Table 1) and therefore could be involved in the AV node dysfunction in HF. The function of some of the transcripts (*Fat1, Fermt2, Fign*, *Lrch1*, *Tmem182*) is either unknown or has an unknown relevance for the AV node; some transcripts are involved in heart development (*Meis1*, *Id2*, Tbx3); two transcripts are known to be involved in HF (*Camk2d*, *Myocd*); and one transcript (*Scn10a*) is known to modulate AV node conduction (Table 1). Three transcripts known to be associated with hereditary AV node dysfunction (*Lmna*, *Prkag2*, *Trpm4*; Table 1) changed significantly in the AV node in HF.

**TABLE 1:** Transcripts which changed significantly in the AV node in HF and the corresponding genes have been identified by GWAS studies as being associated with the PR interval or naturally-occurring mutations in the corresponding genes are associated with AV block. Gene/transcript name, transcript abundance in HF as a percentage of that in control animals, adjusted *p*-value of the change in HF, and notes on the functions of the genes shown.

Gene	HF/control (%)	Adjusted *p*-value	Function
GWAS studies			
*Camk2d*	116.3	0.028	Signalling molecule uniquely positioned to promote HF and arrhythmias; suggested to be causative of sinus node dysfunction Wu and Anderson (2014)
*Fat1*	121.6	0.043	FAT cadherins are evolutionarily conserved cell adhesion molecules, which play key roles in modulating tissue morphogenesis Helmbacher (2018)
*Fermt2*	121.3	0.0055	Important in integrin activation and cell-cell adhesion Yan et al. (2020)
*Fign*	62.0	2.04E-05	Unknown
*Id2*	79.4	0.033	Transcriptional regulator; a molecular pathway including ID2, TBX5 and NKX2-5 is required for cardiac conduction system development Moskowitz et al. (2007)
*Lrch1*	141.5	0.0015	Unknown
*Meis1*	82.7	0.014	Homeobox transcription factor essential for vertebrate heart development and normal physiology of adult heart Dupays et al. (2015)
*Myocd*	188.6	1.71E-10	Transcription factor; upregulated in cardiac tissue from patients with heart disease; upregulation causes left ventricular systolic dysfunction, and impairment of electrical activity and hypertrophy; downregulation attenuates cardiac muscle dysfunction and death Miano (2015)
*Tbx3*	69.3	0.0032	Transcription factor; involved in formation of AV node
*Tmem182*	120.8	0.0092	Unknown
*Scn10a*	54.5	0.0047	Modulator of AV node conduction Park and Fishman (2014)
**Studies of naturally-occurring mutations**			
*Lmna*	120.8	0.0022	Mutations known to be linked to high degree AV block Crasto et al. (2020)
*Prkag2*	163.7	5.13E-05	Gain-of-function mutations known to be linked to AV node dysfunction Gollob (2008)
*Trpm4*	66.0	8.86E-08	Loss-of function mutations known to be linked to AV block Bianchi et al. (2018)

### Other HF-dependent changes

There were many other HF-dependent changes and some of these are considered in the Supplementary Figures S22–S25.

## Discussion

This study has shown that in a model of pressure overload (hypertension)-induced cardiac hypertrophy and HF there is a significant prolongation of the PR interval, evidence of AV node dysfunction as observed in HF patients (Nikolaidou et al., 2016). This was accompanied by significant changes in 5.6% of the transcriptome of the AV node: for example, in transcripts responsible for the PR interval, the sarcomere, fatty acid and glucose metabolism, the extracellular matrix, and the transcription and translation machinery. Importantly, there were also significant changes in transcripts involved in multiple signalling molecules and pathways, all of which have the potential to be involved in cardiac remodelling. Finally, there was evidence of an activation of the immune system and a proliferation or infiltration of macrophages and other immune cells in the AV node. The complexity and multiplicity of effects of HF on the AV node is a novel finding. There are likely to be many more effects on the AV node; the reader can peruse the list of all transcripts in the Supplementary Material (All transcripts. xlsx).

### Potential immediate cause of AV dysfunction

In the AV node in HF there was a downregulation of key ion channel transcripts involved in AV node conduction: *Scn10a* responsible for Nav1.8; *Cacna1d* (Cav1.3) in part responsible for the L-type Ca^2+^ current, *I*_Ca,L_; *Cacna1g* (Cav3.1) and *Cacna1h* (Cav3.2) responsible for the T-type Ca^2+^ current, *I*_Ca,T_; and *Kcnh2* (ERG) responsible for the rapid delayed rectifier K^+^ current, *I*_K,r_. These changes could be responsible for the slowing of AV node conduction and AV node dysfunction (Regan et al., 2005; Chambers et al., 2010; Holm et al., 2010; Pfeufer et al., 2010; Park and Fishman, 2014; Mesirca et al., 2015; Auxerre-Plantié et al., 2019).

### Widespread transcriptomic remodelling of the AV node

Transcriptomic remodelling of the AV node in HF was not restricted to ion channels–there was a remodelling of other systems as well. There was a transcriptomic remodelling of receptors and G proteins, which potentially could impact AV node conduction (Supplementary Figure S22). Some of the transcriptomic remodelling in the AV node in HF is similar to that reported for the rest of the heart, for example, the changes in natriuretic peptide and myosin heavy chain transcripts (Supplementary Figure S2) (Dirkx et al., 2013; Man et al., 2018). The observed changes in metabolic transcripts are qualitatively similar to changes observed in whole heart studies: in a rat TAC model of HF, Bugger et al, (2009) reported that six proteins involved in fatty acid beta-oxidation were downregulated including HADH (downregulation of *Hadh* was observed in this study–Figure 4C), three involved in glycolysis were upregulated, two involved in the tricarboxylic acid cycle were upregulated including aconitase 2 (upregulation of *Aco2* was observed in this study–Figure 4D), and some proteins involved in the electron transport chain were upregulated and some downregulated including subunits of NADH dehydrogenase and cytochrome c oxidase. In the mouse TAC model of HF, a decreased abundance of proteins involved in fatty acid metabolism and increased abundance of proteins involved in glycolysis has been reported in the ventricles (Dai et al., 2012). HF is reported to result in fibrosis and this can be arrhythmogenic by interrupting the conduction of the action potential (Nguyen et al., 2017). However, there was no evidence of upregulation of transcripts for the fibrillar collagens, *Col1* and *Col3*, in the AV node in HF, although there was a widespread transcriptional remodelling of the extracellular matrix (Figure 6 and Supplementary S17-S19).

### Potential causes of the adverse remodelling of the AV node

**FIGURE 8:**
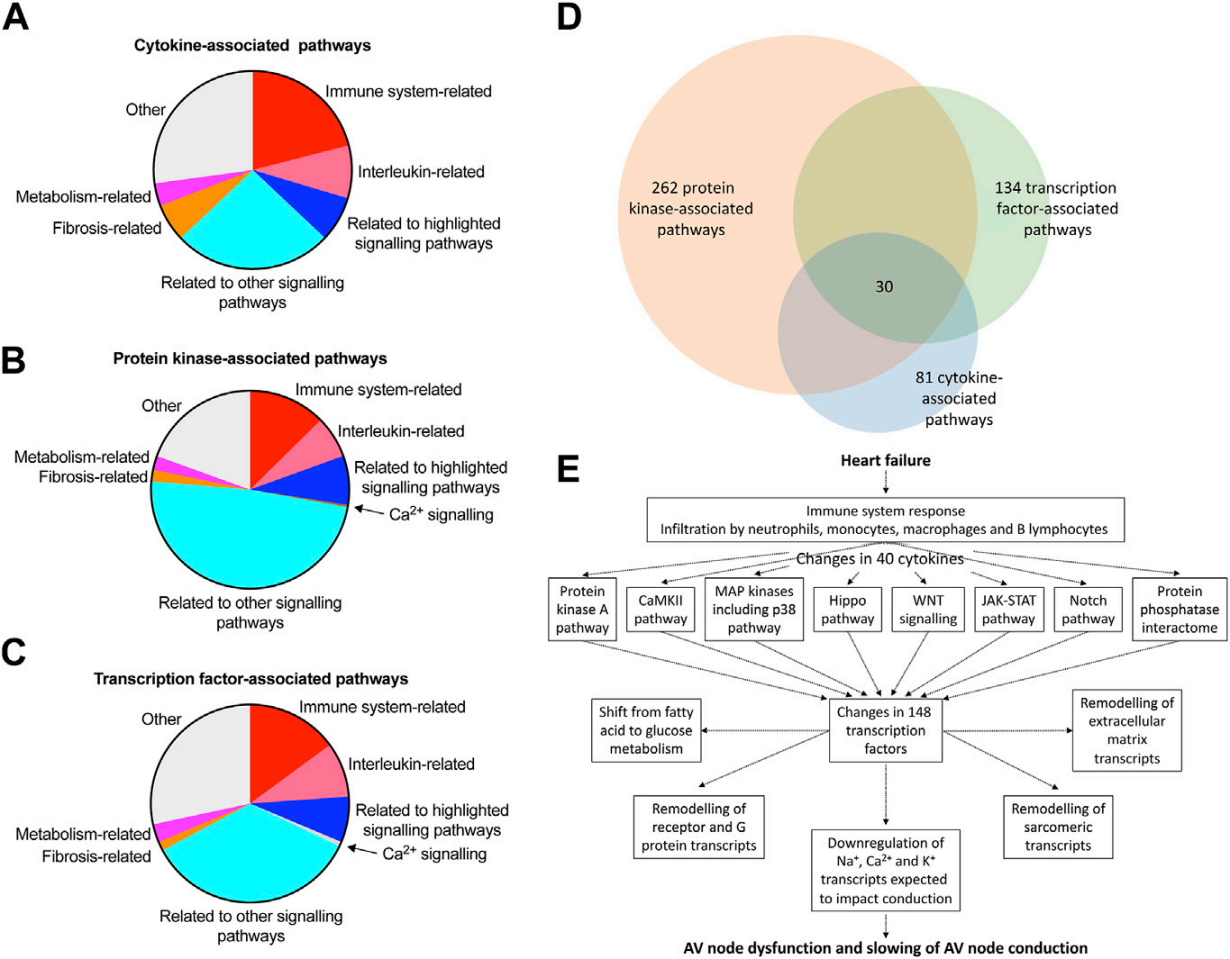
Pathways involved in AV node dysfunction in HF. **(A–C)**, summary of canonical pathways (identified by Ingenuity Pathway Analysis) associated with the cytokines **(A)**, protein kinases **(B)** and transcription factors **(C)** changing significantly in the AV node in HF. Details of the pathways are given in the Supplementary File, Canonical pathways. xlsx. 81, 262 and 134 pathways associated with the cytokines, protein kinases and transcription factors, respectively, were identified in total and the area of the pie chart for each group of pathways reflects the number of pathways in each group. **(D)**, Venn diagram of the pathways associated with the cytokines, protein kinases and transcription factors; there is substantial overlap of the pathways and 30 pathways were common to the cytokines, protein kinases and transcription factors. **(E)**, schematic diagram showing a potential chain of events in the AV node during HF leading to AV node dysfunction. Although the schema is based on the results of this study, it remains hypothetical. Although unidirectional arrows are drawn, they could be bidirectional.

This study has shown a transcriptomic remodelling of the protein kinase A, CaMKII, p38-MAP kinase, Hippo, WNT, JAK-STAT and Notch signalling pathways as well as the protein phosphatase interactome in the AV node in HF; it has also shown evidence of an immune response; each of these has been shown in other studies to be involved in cardiac pathological remodelling in disease. This study has also shown significant changes in signalling molecules: 40 cytokines, 109 protein kinases and 148 transcription factors (Figure 7C, Supplementary S14 and S16). Ingenuity pathway analysis was used to identify the canonical pathways significantly associated with the cytokines, protein kinases and transcription factors and the results are given in a Supplementary file (Canonical pathways. xlsx) and summarised in Figures 8A–C; in each case, many molecules are associated with the immune system especially the interleukin pathway, many are associated with intracellular signalling pathways (both signalling pathways highlighted in this study and many other pathways), and some are associated with Ca^2+^ signalling, metabolism and fibrosis. Figure 8D shows a large amount of overlap between the three sets of associated canonical pathways and this increases confidence in the predictions. The highlighted signalling pathways identified by the canonical pathway analysis include protein kinase A signalling, p38 MAPK signalling, PAK signalling, HIPPO signalling, WNT/β-catenin signalling, JAK/Stat signalling, STAT3 pathway and Notch signalling–this increases the likelihood that these pathways are involved. The involvement of multiple signalling pathways in the AV node in HF is not surprising because there were widespread changes (involving >3,000 transcripts) in the AV node in HF. This raises reservations concerning therapeutic strategies targeting only one pathway.

The activation of the immune system has the potential to be the driver of all the other changes observed in the AV node. The function of the immune system is not only to protect the animal from invasion by foreign organisms. In the case of the heart, it is also concerned with repair of the heart after myocardial infarction (Kologrivova et al., 2021), the adverse remodelling of the working myocardium in heart disease (Strassheim et al., 2019; Murphy et al., 2020), and even ageing (Appel et al., 2021). For the first time, we show here evidence that in HF there is a proliferation of immune cells (neutrophils, monocytes, macrophages and B lymphocytes) in the AV node (Figure 7). One of the principal lines of evidence for a proliferation of neutrophils and monocytes were increases in *S100a8* and *S100a9* to 3,447% and 1,923% of control (Figure 7B). During inflammation, S100A8/A9 is actively released and exerts a critical role in modulating the inflammatory response and serves as a candidate biomarker for disease (Wang et al., 2018). S100A8/A9 plasma levels are significantly higher in chronic HF patients (Wang et al., 2018).

In the AV node in HF there was an increase in transcripts for proinflammatory cytokines (and proinflammatory cytokine-related molecules): *Il1b* (IL-1b), *Osm* (oncostatin M; cytokine of interleukin-6 family), *Tnfrsf1a* (TNF receptor 1), *Tnfrsf1b* (TNF receptor 2), *Tgfb1* (TGFβ-1) and *Tgfb2* (TGFβ-2) (Figure 7C). *Lgals3*, responsible for galectin-3, which can have cytokine-like regulatory actions in immune cells (Jeon et al., 2010), was also significantly upregulated in the AV node in HF (Supplmentary Figure S20C). These upregulated cytokines and cytokine-related molecules have a known role in HF: *Il1b* is upregulated in HF and associated with worse prognosis and trials have been undertaken to evaluate the role of interleukine-1 blockade to reduce inflammation and ventricular remodelling in HF (Van Tassell et al., 2015; Szekely and Arbel, 2018). There is evidence that IL-1b downregulates *I*_Ca,L_ and K^+^ current and Ca^2+^-handling proteins (Szekely and Arbel, 2018). Patients suffering chronic dilated cardiomyopathy show upregulation of oncostatin M and its receptor, Oβ (oncostatin M receptor; *Osmr*) (Kubin et al., 2011). Cardiac oncostatin M (*Osm*) induces dedifferentiation of cardiomyocytes and promotes progression of HF, and an antibody targeting Oβ or genetic inactivation of a single allele of *Osmr* reduces cardiomyocyte remodelling and dedifferentiation and improves cardiac performance and survival (Kubin et al., 2011; Pöling et al., 2013). TNF-α and its receptors have both pathogenic and cardioprotective roles in heart disease (Rolski and Błyszczuk, 2020). In the mouse TAC model of HF, there is a correlation between progressive hypertrophy and increasing myocardial levels of TNF-α and TNF receptor 1 (Rolski and Błyszczuk, 2020). Increased concentrations of circulating TNF receptors 1 and 2 are associated with increased risks of cardiovascular events and mortality in patients with stable coronary heart disease (Carlsson et al., 2018). TGF-β is another cytokine. Myocardial TGF-β expression is upregulated in animal models of myocardial infarction and cardiac hypertrophy and in patients with dilated or hypertrophic cardiomyopathy, and it plays an important role in cardiac remodelling and fibrosis (Dobaczewski et al., 2011). Galectin-3 overexpression and secretion is associated with several diseases including HF, in which galectin-3 has been shown to be a useful biomarker (Gehlken et al., 2018; Suthahar et al., 2018). Galectin-3 induces pathologic remodelling of the heart, and is a culprit in the development of cardiac fibrosis in HF and electrical remodelling in atrial fibrillation (Takemoto et al., 2016; Gehlken et al., 2018). Proof that inflammation is the cause of AV node remodelling and dysfunction in HF is beyond the scope of this study. However, if it is the cause there may be a correlation between the two and with one exception there are significant correlations between the two well-known heart failure markers, *Nppa* and *Nppb* (responsible for ANP and BNP) and the macrophage marker, *Cd14*, the cytokine transcript, *Crlf1*, and the transcript for the cytokine-like galectin-3, *Lgals3* (Supplementary Figure S21).

In conclusion, the ultimate reason for the dysfunction of the AV node in HF is likely to be the downregulation of Na^+^, Ca^2+^ and K^+^ channel transcripts, as a result of transcription factors—as well as micro-RNAs (Yanni et al., 2020). It is feasible that these changes are driven by the protein kinase A, CaMKII, p38-MAP kinase, Hippo, WNT, JAK-STAT and Notch signalling pathways as well as the proliferation of immune cells and upregulation of cytokine transcripts in the AV node in HF. Figure 8E presents a hypothetical scheme of the possible hierarchy of changes. In addition to this, the changes in transcripts known to be related to AV block listed in Table 1 have also to be considered.

## Data Availability

The raw data from this study are available in a repository:https://www.ebi.ac.uk/biostudies/arrayexpress/arrayexpress/studies/E-MTAB-12384?accession=E-MTAB-12384. Further data are available in the Supplementary Material.

## Abbreviations

AV, atrioventricular; ECG, electrocardiogram; HF, heart failure; RNAseq, RNA-sequencing; TAC, transverse aortic constriction.
