# Supplementary material for: Atrioventricular node dysfunction in pressure overload-induced heart failure—Involvement of the immune system and transcriptomic remodelling
Source: Front Pharmacol. 2023 Apr 4;14:1083910. doi: 10.3389/fphar.2023.1083910 (PMC10110994; doi:10.3389/fphar.2023.1083910)
Supplement: Supplementary file 2 [file DataSheet1.pdf]

SUPPLEMENTARY MATERIAL
Atrioventricular node dysfunction in pressure overload-induced heart failure – involvement of the immune system and transcriptomic remodelling

Claire Wilson, Min Zi, Mathew Smith, Munir Hussain, Alicia D'Souza,
Halina Dobrzynski and Mark Boyett

SUPPLEMENTARY MATERIALS AND METHODS

Generation of heart failure (HF) mouse model

Animal studies were performed in accordance with the United Kingdom (Scientific Procedures Act) 1986. Ethical approval was provided by the University of Manchester Ethics Committee. During the study, animals were housed in a temperature and humidity-controlled room, with free access to food and water. Hypertension is the commonest cause of HF: although the risk of HF associated with hypertension is smaller than that associated with myocardial infarction, hypertension is more prevalent; 75% of the HF cases in the Framingham cohort are associated with hypertension (Bui *et al.*, 2011). In this study, a pressure overload (hypertension) model of HF was used. Male eight-week-old C57Bl/6N mice (Charles River) underwent transverse aortic constriction (TAC; HF) or sham (control) operations as previously described (Yanni *et al.*, 2020). Animals were induced with 5% isoflurane in 100% O_2 and intubated orally. Throughout the surgery, anaesthesia was maintained at 3% isoflurane in 100% O_2 using a ventilator set to 200 breaths/minute and 0.1 ml tidal volume (Minivent 845, Harvard Apparatus). To begin the surgery, a mini-thoracotomy was performed and the aortic arch visualised. A 27-gauge needle was placed over the aorta at the point between the brachiocephalic trunk and the left common carotid artery. A 7-0 prolene suture was wrapped around the needle and tied. This constriction has been shown to result in a pressure gradient of 25-30 mmHg between the right and left carotid arteries (Mohamed *et al.*, 2016) The chest was then closed using 6.0 prolene sutures. Analgesia in the form of buprenorphine (0.1 mg/kg) was administered intraperitoneally. Sham-operated animals underwent surgery in which the aortic arch was exposed, and a suture was passed under the artery and removed without tying. Following surgery, animals were left to recover in a 30°C incubator for up to 5 h before returning to normal housing. Animals were terminated eight weeks post-surgery or when HF symptoms (lethargy, dyspnea, weight loss or >20% loss of body weight) developed. Animals were terminated by cervical dislocation.

Electrocardiogram (ECG) recordings from the conscious mouse

Heart rate measurements from the conscious mouse were collected every week post-surgery and were obtained non-invasively using an ECGenie enclosure (Mouse Specifics). The enclosure consists of an elevated platform embedded with ECG electrodes. Electrodes are connected to an amplifier (e-MOUSE), with contact between the animal's paws and the electrodes resulting in an ECG signal. The signal was recorded through a PowerLab/4SP (ADInstruments) and analysed using LabChart (ADInstruments, Version 8). Prior to data collection, animals were allowed to acclimatise to the set up for 10 min. Heart rate was measured over 100 beats from continuous recordings at a sampling rate of 2 kHz, with a high-pass filter set at 100 Hz and low-pass filter set at 3 Hz. A peak detection algorithm on LabChart was used to identify R-waves from which conscious heart rate was calculated.

Echocardiography

An echocardiogram was performed seven weeks post-surgery using a Vevo 77 high-resolution imaging system (Fujufilm Visualsonics, Canada) fitted with a 14 MHz transducer. Mice were induced in 1.5% isoflurane in 100% O_2 and maintained at 1% isoflurane in 100% O_2 via a Fluovac anaesthetic facemask connected to a coaxial unit (Harvard Apparatus). Hearts were imaged in the parasternal short-axis view and M-mode echocardiography was recorded. From the recordings, left ventricular end diastolic diameter (LVEDD), left ventricular end systolic diameter (LVESD), posterior wall thickness in diastole (LVPWd) and systole (LVPWs), and interventricular septal thickness in diastole (IVSd) and systole (IVSs) were measured using the leading-edge method over three cardiac cycles. From these measurements, further indices were calculated:

Fractional shortening (%) = $((\mathrm{LVEDD} - \mathrm{LVESD})/\mathrm{LVEDD}) \times 100$

Ejection fraction (%) = $((\mathrm{LVEDD}^3 - \mathrm{LVESD}^3)/\mathrm{LVEDD}^3) \times 100$

Left ventricular mass (mg) = $((\mathrm{IVSd} + \mathrm{LVEDD} + \mathrm{LVPWd})^3 - \mathrm{LVEDD}^3) \times 1.05$

ECG recordings from the unconscious mouse

Prior to termination, the ECG was recorded from the unconscious mouse using a three-lead system. Mice were induced in 3% isoflurane in 100% O_2, and anaesthesia was maintained at 2% isoflurane in 100% O_2 via a Fluovac anaesthetic facemask connected to a coaxial unit (Harvard Apparatus). Animals were placed in a supine position on a heat pad connected to an Animal Temperature Controller (WPI). Body temperature was maintained at 36-37°C. Electrodes (WPI) were inserted subcutaneously in the lead II arrangement (left and right forelimbs and left hindlimb), and ECG signals were recorded. Signals were amplified using a ML136 Dual Bio amplifier (ADInstruments) and a PowerLab/4SP (ADInstruments) was used to record signals at a sampling frequency of 2 kHz. ECG parameters were analysed using LabChart (ADInstruments, Version 8) and averaged over 100 consecutive beats. P, Q, R, S and T wave positions were manually verified and refined, with waveform measurements based on deflections from the isoelectric line. The end of the T wave was defined as the point at which the wave returned to the isoelectric baseline, which included the low-amplitude portion of the T wave. QT interval was corrected for heart rate (QTc) using the Mitchell equation:

$$QTc = QT/(RR/100)^3$$

Statistical analysis of physiological measurements

Statistical analysis was performed using GraphPad Prism 8 (GraphPad Software). Differences between body weight and heart rate at different time points post-surgery were tested using a two-way ANOVA with a Bonferroni post hoc test. To assess the difference between single time point measurements in HF and control animals, Student's t-test was used. Normal distribution of data was tested using the Shapiro-Wilk test and equal variance was tested using the F-test. When the null hypothesis of normality and/or equal variance was rejected, the non-parametric Mann-Whitney test was used. In figures, data are shown as mean±SEM. A P value of <0.05 was regarded as significant, with an asterisk indicating significance on figures.

Tissue collection

Mice were culled by cervical dislocation and the hearts were quickly removed and placed in Tyrode solution (100 mM NaCl, 4 mM KCL, 1.2 mM $MgSO_4$, 1.2 mM KH_2PO_4, 1.8 mM $CaCl_2$, 25 mM $NaHCO_3$, 10 mM glucose, pH 7.4). The atrioventricular (AV) node was dissected out, snap frozen and stored at -80°C until use. AV node biopsies were collected from the triangle of Koch bordered by the coronary sinus, tendon of Todaro and tricuspid valve annulus (Li *et al.*, 2008); the base, height and area of the triangle was ~0.5 mm, ~0.75 mm and 0.19 mm^2, respectively. AV node tissue isolated from three mice was pooled. Three biological replicates (each a pool from three mice) were isolated from control and HF mice. Subsequent experiments and data analysis were performed blind.

RNA extraction and sequencing

Total RNA was isolated from the pooled AV node biopsies using a RNeasy Micro kit (Qiagen) according to the manufacturer's instructions. Following RNA extraction, total RNA was submitted to the Genomic Technologies Core Facility (University of Manchester). RNA quantity and integrity were measured using a 2200 TapeStation (Agilent Technologies). TrueSeq Stranded mRNA assays (Illumina) were used to produce stable, single-stranded cDNA libraries, as previously described (Wang *et al.*, 2021b). Poly-A containing mRNA was purified using magnetic separation technology. The purified mRNA sequences were then fragmented into parts using divalent cations under elevated temperatures. Single-stranded cDNA was synthesised from RNA fragments using reverse transcriptase and random primers, followed by second strand cDNA synthesis using DNA Polymerase I and RNase H. The final cDNA library was generated by an addition of a single 'A' base, binding of adapters to the fragments and purification and enrichment via a PCR reaction. cDNA libraries were multiplexed, then pooled and clustered using a cBlot instrument (Illumina). mRNA samples were then pair-end sequenced and mRNA was quantified using a HiSeq4000 instrument (Illumina). Sequence adapter identification and QC trimming were performed using BBMerge and

BBDuk from BBMap v36.32 (DOE Joint Genome Institute; https://sourceforge.net/projects/bbmap/). Genome mapping was performed with STAR v2.7.2b (with 2-pass mapping; Cold Spring Harbor Laboratory; https://github.com/alexdobin/STAR) against the reference mouse genome Mouse mm10 with Gencode vM24 annotation (GENCODE; https://www.gencodegenes.org/mouse/release_M24.html). Normalisation was carried out using DESeq2 v1.26.0 and the normalised counts were plotted.

Statistical analysis of gene expression

For RNA-Seq data, P values were corrected for multiple testing using the Benjamini-Hochberg method to control the false discovery rate. In figures, data are shown as mean±SEM. A P value of <0.05 was regarded as significant, with asterisks indicating significance on figures. Hierarchical clustering was performed using Pearson's correlation distance and ward.D2 agglomeration method and a heatmap was generated (Figure 2C) for the 3,077 differentially expressed transcripts (adjusted P value <0.05) using ComplexHeatmap v2.2.0 (https://www.bioconductor.org/packages/release/bioc/html/ComplexHeatmap.html). The Z-score is the number of standard deviations a given data point lies above or below the mean.

Gene ontology (GO) enrichment analysis

Enrichment analysis was performed using the R package topGO v2.38.1 (Bioconductor; https://bioconductor.org/packages/release/bioc/html/topGO.html). Analysis was performed on downregulated and upregulated differentially expressed genes identified using RNAseq. Significance was tested with Fisher’s exact test and the elim algorithm. Significantly enriched biological processes related to cardiac function and HF were selected.

Qiagen Ingenuity Pathway Analysis (IPA)

IPA (https://www.qiagenbioinformatics.com/products/ingenuity-pathway-analysis) was used for canonical pathway analysis. All significantly expressed transcripts were entered into IPA and core analysis was performed. Default parameters were used for the analysis. Right-tailed Fisher’s exact test was performed to assess the statistical significance of enriched pathways. P<0.05 was considered significant.

SUPPLEMENTARY RESULTS

Further information on electrophysiological changes, upregulation of HF markers, and remodelling of ion channel and related transcripts in HF

The heart rate measured in the conscious mouse using an ECGenie steadily declined following TAC surgery (Figures 1F and S1G) as a result of sinus node dysfunction (Yanni *et al.*, 2020). There was also a significant increase in the QRS interval indicative of His-Purkinje dysfunction (a slowing of conduction through the His-Purkinje system) and uncorrected and corrected QT intervals indicative of an increase in ventricular action potential duration by the end of the experiment in the HF mice (Figure 1H-J).

It is well known that *Nppa* (responsible for atrial natriuretic peptide, ANP), *Nppb* (responsible for brain natriuretic peptide, BNP) and *Myh7* (responsible for β-myosin heavy chain) are upregulated in HF (Man *et al.*, 2018; Dirkx *et al.*, 2013) and all three transcripts were significantly upregulated in the AV node in HF (Figure S2).

Knockout of *Hcn4* results in AV block (Baruscotti *et al.*, 2011). There was a ~25% downregulation of *Hcn4* in HF, but it was not significant (Figure S4A). Seven Clcn transcripts for Cl^- channels were detected (*Clcn4*>*Clcn7*>*Clcn3*>*Clcn6*>*Clcn1*>*Clcn5*>*Clcn2*) and of these *Clcn1* and *Clcn2* were significantly downregulated (Figure S4B). The downregulation of *Clcn2* may be important, because the CLCN2 channel carries slowly activating inward current at diastolic potentials like the HCN4 channel and has been shown to contribute to pacemaking in the sinus node (Huang *et al.*, 2009). The downregulation of *Clcn1* may also be important, because myotonic dystrophy patients have abnormal splicing of *Clcn1* and an increased incidence of a prolonged PR interval and AV block (McNally and Sparano, 2011) (although the abnormal *Clcn1* splicing may only be associated with the AV node dysfunction and not the cause of it).

Inhibition of the ‘Ca^{2+} clock’ can slow AV node conduction and increase the PR interval (Saeed *et al.*, 2018). As already discussed, some Ca^{2+} channel transcripts were downregulated. However, other Ca^{2+} clock transcripts were ether unaffected or upregulated; for example, *Slc8a1*

and *Casq2*, responsible for the Na^+-Ca^{2+} exchanger and calsequestrin 2, were both significantly upregulated (Figure S5). Via the Na^+-Ca^{2+} exchanger, the intracellular Ca^{2+} concentration is closely linked to the intracellular Na^+ concentration, which is set by the Na^+-K^+ pump. In HF, there was a significant upregulation of *Atp1a1* (α1 isoform of the Na^+-K^+ pump) and a significant downregulation of *Atp1a2* (α2 isoform) (Figure S6). In the heart, the α2 isoform preferentially assembles with the β2 isoform (*Atp1b2*) (Clausen *et al.*, 2017) and in HF *Atp1b2* was downregulated by a similar percentage as *Atp1a2* (Figure S6). The α2 isoform localises close to the Na^+-Ca^{2+} exchanger and may help in the regulation of intracellular Ca^{2+} (Clausen *et al.*, 2017). The FXYD family including phospholemman (FXYD1) has been demonstrated to regulate the Na^+-K^+ pump; two of the *Fxyd* transcripts showed significant changes (Figure S6C).

Gap junctions made up of connexins are key determinants of action potential conduction, but no changes in connexin transcripts known to be linked to AV node conduction were detected (Figure S7).

Evidence of activation of multiple intracellular signalling pathways and transcription factors in HF

Protein kinase A. The role of the sympathetic nervous system and the β-adrenergic receptor pathway in causing or exacerbating cardiac disease has long been recognised (Bernstein *et al.*, 2011). β-adrenergic receptor stimulation leads to an activation of protein kinase A via adenylate cyclase and a G protein. In the AV node in HF, there was no change in transcripts for β-receptors, the G protein or adenylate cyclase (see below). Protein kinase A is made up of catalytic and regulatory subunits and whereas catalytic subunit transcripts were unaffected, regulatory subunit transcripts were affected; *Prkar1a* was the most abundant of the regulatory subunit transcripts and it was upregulated by 76% (Figure S10A). A similar pattern of change in protein kinase A subunits (protein) has been observed in HF in the human (Han *et al.*, 2013). Phosphodiesterases able to hydrolyse cAMP (Figure S10B,C) antagonise protein kinase A by hydrolysing cAMP. In the AV node in HF, the expression of the most abundant of the cAMP-selective phosphodiesterase transcripts, *Pde4a*, was downregulated (Figure S10C). Ablation of Pde4 has been shown to affect excitation-contraction coupling and predispose to the development of HF; downregulation of Pde4a is observed in the failing human heart (Richter *et al.*, 2011). Expression of some transcripts for other phosphodiesterases able to hydrolyse cAMP were also significantly changed or showed a trend towards a change in HF (Figure S10B,C). In contrast, there were no changes in cGMP-selective phosphodiesterase transcripts (Figure S10D).

Ca^{2+}-calmodulin-dependent protein kinase II (CaMKII). CAMK2D is upregulated in HF, and transgenic mice overexpressing *Camk2d* develop a dilated cardiomyopathy (Zhang *et al.*, 2003). Sinus node dysfunction in HF has been attributed to activation of CaMKII (Swaminathan *et al.*, 2011). Transcript for the dominant CaMKII isoform, *Camk2d*, was significantly upregulated in the AV node in HF (Figure S11).

Hippo pathway. Studies have demonstrated a role for the Hippo pathway in cardiac disease (Ikeda *et al.*, 2019; Chen *et al.*, 2020). On stimulation, MST1/2 and its adaptor protein, SAV1, are phosphorylated and activated, and they in turn phosphorylate and activate the LATS1/2-MOB1 complex (Figure S12A). The activated LATS1/2 phosphorylates YAP1 leading to the cytoplasmic retention and possible degradation of the YAP1/TAZ complex (Figure S12A) (Chen *et al.*, 2020). When the Hippo pathway is inactive, YAP1/TAZ is mainly localised in the nucleus and together with transcriptional partners (e.g. TEADS) initiates or impedes transcription of target genes (Figure S12A) (Chen *et al.*, 2020). The Hippo pathway can be cardioprotective in HF, although it can also exacerbate it (Chen *et al.*, 2020). Clinical ischaemic heart disease and idiopathic dilated cardiomyopathy is characterised by increased YAP1/TAZ protein levels and transcriptional activity and consequent upregulation of target genes such as *Ccn2* (responsible for connective tissue growth factor, CTGF) (Chen *et al.*, 2020). CTGF is a central mediator of tissue remodelling and fibrosis and its inhibition can reverse the process of fibrosis (Lipson *et al.*, 2012). Inactivation of CTGF (using a monoclonal antibody) in mice following myocardial infarction reduced the heart weight:body weight ratio, left ventricular mass, cardiomyocyte hypertrophy, and fibrosis (Vainio *et al.*, 2019). There were many changes to the Hippo pathway in the AV node in HF (Figure S12B). Many changes potentially favour nuclear localisation of YAP1/TAZ. In addition, *Tead1*, *Tead4* and *Wwtr1*, necessary for YAP target gene transcription, were upregulated. One of the target genes at least, *Ccn2* (CTGF), was upregulated (Figure S12B).

WNT signalling. Although WNT-signalling is quiescent under normal conditions, it is activated by pathological stress, and activation of WNT signalling is sufficient for the induction of cardiac hypertrophy and cardiomyopathy (Malekar *et al.*, 2010; Zhao *et al.*, 2018; Foulquier *et al.*, 2018). WNT protein binds to a Frizzled receptor; co-receptors (LRP5, LRP6, ROR1 and ROR2) may be required (Foulquier *et al.*, 2018). This causes an accumulation of β-catenin in the cytoplasm and its eventual translocation into the nucleus to act as a transcriptional coactivator of transcription factors. Various *Wnt* transcripts (including the most abundant, *Wnt9b*), various *Fzd* transcripts responsible for Frizzled receptors (including the most abundant, *Fzd4*), and *Ctnnb1* (responsible for β-catenin) were significantly upregulated in HF (Figure S13).

Protein kinases and the protein phosphatase interactome. Protein phosphorylation is an important signalling mechanism, and it is determined by a balance of protein kinases and phosphatases. Some kinases have already been considered; in total there were significant changes in 109 protein kinase transcripts (Figure S14). Some phosphatases (DUSPs) have again already been considered. Protein phosphatase type-1 (PP1) plays an important role in cardiac physiology and pathophysiology (Chiang *et al.*, 2016; Chiang *et al.*, 2018). PP1 is known to play an important role in HF and some studies have reported the activity of PP1 to be increased in HF (presumably all what is known relates to the ventricles) (Chiang *et al.*, 2016). PP1 is made up of a catalytic subunit and regulatory subunits. Transcript for the catalytic subunit has been reported to be upregulated in HF patients (Chiang *et al.*, 2016). Two regulatory subunits, PPP1R3A (Cordero *et al.*, 2019) and PPP1R7 (Chiang *et al.*, 2018), have been implicated in HF; mice lacking PPP1R3A are protected against HF (TAC model) (Cordero *et al.*, 2019). In this study, transcript for one of two abundant catalytic subunit isoforms, *Ppp1cb*, was significantly upregulated in the AV node in HF (Figure S15). There was a significant upregulation of the regulatory subunit *Ppp1r3a*, but not of *Ppp1r7* (Figure S15). There was a significant up and down regulation of transcripts for other regulatory PP1 subunits (Figure S15). The largest upregulation was of *Ppp1r3c* (Figure S15). PPP1R3C is involved in the control of metabolism: during hypoxia, hypoxia-inducible factor 1 (HIF1) promotes glycogen accumulation by regulating PPP1R3C (Shen *et al.*, 2010). In this study, *Hif1a*, as well as *Ppp1r3c*, was significantly upregulated in the AV node in HF (Figure S15). In this study, another regulatory subunit, *Ppp1r1a*, was significantly downregulated in HF (Figure S15). This is significant, because PPP1R1A is a potent inhibitor of the catalytic subunit of PP1 and a downregulation of PPP1R1A in HF has been observed in other studies (Chiang *et al.*, 2016). In the present study, in HF, an upregulation of various subunits for protein phosphatases types 2, 3 and 4 (PP2-4) was also observed (Figure S15). PP2 is a critical regulatory molecule in both health and disease, with a myriad of targets in heart (DeGrande *et al.*, 2013; Lubbers and Mohler, 2016). There can be an increase in PP2 in HF, including in the human, and modulation of PP2 may contribute to the pathophysiology of cardiac disease (DeGrande *et al.*, 2013; Lubbers and Mohler, 2016).

Other pathways. In addition to the pathways above, there was evidence of changes in the JAK-STAT pathway (considered below) and the NOTCH pathway (significant downregulation of *Notch2* to 85% of control and significant upregulation of *Notch4* to 173% of control).

Transcription factors. Transcript expression is primarily governed by transcription factors, and in the mouse Zhou et al. (Zhou *et al.*, 2017) have identified 941 transcription factors. Of these, transcripts for 927 were identified in the AV node and 148 (16%) changed significantly in HF (Figure S16). 81 (55%) were upregulated and 67 (45%) downregulated (Figure S16).

Remodelling of further extracellular matrix transcripts in HF

Glycoproteins and proteoglycans. Glycoproteins make the extracellular matrix a cohesive network of molecules (Megías *et al.*, 2019). They link structural molecules to each other, and also to cells (Megías *et al.*, 2019). Fibronectins, laminins and tenascins are major glycoproteins (Megías *et al.*, 2019). Fibronectin (encoded by *Fn1*) binds integrins in the cell membrane to components of the extracellular matrix such as collagen and fibrin (Megías *et al.*, 2019); *Fn1* was significantly upregulated in HF (Figure S17A). Laminins are cell adhesion molecules found predominantly in basement membranes (Megías *et al.*, 2019); one of their functions is to interact with receptors in the cell membrane and thereby regulate signalling pathways (Megías *et al.*, 2019); there were significant changes in three laminin subunit transcripts in HF (Figure S17A). Tenascin-C (*Tnc*) is a large extracellular matrix glycoprotein, which is upregulated during physiological and pathological remodelling and is involved in important signalling pathways (Imanaka-Yoshida *et al.*, 2020); *Tnc* was significantly upregulated in the AV node in HF (Figure S17A). Elastin (encoded by *Eln*) is a

glycoprotein and provides elasticity unlike the collagens (Wang *et al.*, 2021a) and *Eln* was significantly upregulated in HF (Figure S17A); will this increase the elasticity of the sinus node? Proteoglycans are a major component of the extracellular matrix and form the "filler" substance between cells (https://en.wikipedia.org/wiki/Proteoglycan), but only two proteoglycan transcripts *Bgn* and *Chadl* were significantly affected in HF (Figure S17A). Biglycan (encoded by *Bgn*) is related to inflammation: upregulation in adipose tissue during inflammation may be involved in the perpetuation of the inflammatory milieu (Adapala *et al.*, 2012).

Extracellular matrix affiliated. There are 165 'extracellular matrix affiliated' transcripts listed in Matrisome and there were 39 significant changes (Figure S17B).

Secreted factors. There are 367 'secreted factors' listed in Matrisome and these include signalling molecules known to affect the extracellular matrix; there were 61 significant changes (Figure S18A). TGF-β and WNT signalling are known to play an important role in cardiac fibrosis (Yousefi *et al.*, 2020) and *Tgfb1*, *Tgfb2, Wnt4, Wnt7a* and *Wnt9b* were all upregulated as already discussed (Figures 7C and S18A).

Extracellular matrix regulators. There are 304 extracellular matrix regulators listed in Matrisome and these include the metalloproteinases (*Mmp*) and ADAMTS proteases (*Adamts*) for example (Santamaria and de Groot, 2020); there were 55 significant changes (Figure S18B).

Of the 274 'core Matrisome' and 836 'Matrisome associated' transcripts listed in the Matrisome, 66 and 155 showed significant changes (Figure S19).

Further evidence of an immune response in HF

A large number of cytokine transcripts were significantly altered in the AV node in HF (Figure 7C). Two cytokines, IL-1b (interleukin 1 beta; *Il1b*) and TNF-α (tumour necrosis factor-α, *Tnf*), play a particularly important role in the inflammatory response. The transcript for IL-1b, an interleukin secreted by immune cells including monocytes and macrophages (Lopez-Castejon and Brough, 2011), was significantly upregulated in the AV node in HF (Figures 7C and S20A). IL-1b is well known to be upregulated in HF and is thought to be involved in HF development (Van Tassell *et al.*, 2015). The upregulation of *Il1b* in the AV node is therefore an important (and novel) finding. Figure S20A shows changes in various transcripts involved with interleukins. TNF-α has again been implicated in HF development; for example TNF-α overexpression results in ventricular hypertrophy and dilatation, interstitial fibrosis, apoptosis, and a diminished ejection fraction (Hori and Yamaguchi, 2013). Although there was no change in *Tnf* in the AV node in HF, there were changes in other transcripts involved with TNF-α (Figure S20B). For example, there was an upregulation of *Tnfrsf12a* (TWEAK), a positive regulator of cardiomyocyte proliferation (Novoyatleva *et al.*, 2009), and *Tnfrsf1a* and *Tnfrsf1b*, which encode two receptors for TNF-α (Figure S20B). Members of the transforming growth factor-β (TGF-β) superfamily are known to be activated in HF (Hanna and Frangogiannis, 2019). The TGF-β system stimulates myocyte hypertrophy and cardiac fibrosis (Kapur, 2011). In the AV node in HF there were significant changes in TGF-β superfamily transcripts: there was an upregulation of *Tgfb1*, *Tgfb2*, *Gdf6* and *Gdf15*, but a downregulation of *Gdf7*, *Gdf10*, *Bmp1*, *Bmp3* and *Bmp5* (Figure 7C).

Galectin-3 (*Lgals3*), a β-galactoside-binding lectin, has been proposed to have multifaceted functions in various pathophysiological conditions and can exert cytokine-like regulatory actions in immune cells (Jeon *et al.*, 2010). Elevated serum galectin-3 levels have been detected in patients with almost all types of cardiovascular disease (including HF) and are considered as a biomarker of fibrosis and inflammation and may predict morbidity and mortality (Dong *et al.*, 2018). *Lgals3* was upregulated over three-fold in the AV node in HF (Figure S20C). Galectin-3 exerts cytokine-like regulatory actions through the JAK-STAT pathway (Jeon *et al.*, 2010) and the most abundant STAT transcript, Stat3, was significantly upregulated (Figure S20C).

Remodelling of receptor and G protein transcripts in HF

Although *Chrm2* (responsible for the M2 acetylcholine receptor) was unaffected in HF (Figure S22A), transcripts for one of the principal effectors of the M2 receptor, the ACh-activated K^+ channel, *Kcnj3* and *Kcnj5*, were downregulated as already shown (Figure 3C); there were also some changes in the G protein subunits mediating the effects (Figure S22B). The adenosine A1 receptor also works via the ACh-activated K^+ channel; *Adora1* was reduced by 22.2%, but only approached significance (P=0.072) (Figure S22A). Unexpectedly, *Adrb1*, *Adrb2*, and *Adrb3* (responsible for β-adrenergic receptors) were unaffected in HF (Figure S22A), but as discussed above transcripts for some

regulatory subunits of protein kinase A working downstream of the β-adrenergic receptors were affected. *Adra1b*, the most abundant α-adrenergic receptor transcript, was significantly downregulated as was *Adra1d* (Figure S22A); interestingly *Adra1b* was more abundant than the β-adrenergic receptor transcripts (Figure S22A). α-adrenergic receptors are believed to be cardioprotective in HF (Jensen *et al.*, 2014; O'Connell *et al.*, 2013). Transcripts for the mineralocorticoid receptor (*Nr3c2*; activated by aldosterone, deoxycorticosterone and cortisol) and thyroid hormone receptor-α (*Thra*) were downregulated in HF (Figure S23A). Recently, sinus node cells have been shown to express functional glutamate receptors (Liang *et al.*, 2021) and glutamate receptor transcripts were expressed in the AV node and some were downregulated in HF (Figure S23B).

Remodelling of sarcomeric transcripts in HF

There were significant changes of sarcomeric transcripts in the AV node in HF (Figure S24). Just one change will be highlighted: in HF the ratio of *Myh6*:*Myh7* (α-myosin heavy chain:β-myosin heavy chain) changed from 50.7 to 4.8.

Changes in transcription, translation, and mRNA transcript and protein breakdown underlying the remodelling of the AV node in HF

The remodelling of the AV node in heart failure will involve an increase in transcription and translation of some genes as well as the degradation of some mRNAs and proteins. Figure S25 shows a schematic diagram of the processes involved in in transcription, translation, and mRNA transcript and protein breakdown as well transcripts involved in these processes showing significant changes in HF. Transcription is controlled by histone acetyltransferases (HATs), which make chromatin accessible for transcription, and deacetylation of histones by histone deacetylases (HDACs) results in closed chromatin structure and the inhibition of gene transcription (although HDACs deacetylate many nonhistone proteins as well; McKinsey, 2011). Although there were no changes in HAT transcripts, there was a downregulation of three HDAC transcripts including *Hdac5* in the AV node in HF (Figure S25). Reduced expression of HDACs results in a more open chromatin state to allow gene transcription to occur. In the mouse, knockout of *Hdac5* results in exaggerated cardiac hypertrophy in response to pressure overload and spontaneous pathologic hypertrophy with advancing age (McKinsey, 2011). However, HDACs also have a protective effect and HDAC inhibitors have been identified as a promising therapeutic approach in the treatment of HF (McKinsey, 2011). Eukaryotic initiation factors (EIFs) are involved in the initiation of translation and driving protein synthesis. In HF in the AV node there was an increase in numerous EIFs (Figure S25) suggesting an increase in protein translation rates, a hallmark of HF (Simpson *et al.*, 2020). RNA polymerases are essential for protein translation. In HF in the AV node, there was an increase in *Polr1e*, a subunit of polymerase I (Figure S25). Polymerase I is involved in the synthesis of ribosomal RNA which is essential for protein synthesis (Goodfellow and Zomerdijk, 2013). There was also an increase in Mpp6 (Figure S25), which is again involved in the generation of ribosomal RNA (Schilders *et al.*, 2005). At steady-state, protein degradation has to match translation and, if there is an increase in protein translation rates in HF in the AV node, it is arguable that there should be a concurrent increase in protein degradation. Proteins are tagged for degradation by ubiquitination catalysed by ubiquitin ligases. Once ubiquinated, the protein is degraded by the proteasome. In HF in the AV node, there was an increase in numerous proteasome subunits (Figure S25).

SUPPLEMENTARY DISCUSSION

In this study, an omics technology, RNAseq, was used to investigate changes in the whole transcriptome. In comparison, conventional hypothesis-driven-research is more focussed. Conventional hypothesis-driven-research channels the investigator's efforts and provides a framework for progressing knowledge in a methodical stepwise manner. However, it also blinkers the investigator to the complexity of biological systems potentially leading to erroneous conclusions (Boyett and Lundby, 2020). Omics technologies allows the alternative research strategy of data-driven-discovery, and does not constrain the view of the investigator (Boyett and Lundby, 2020).

Comparison of changes in ion channel expression in the AV node in this and earlier studies of HF

In the mouse model of hypertension-induced HF in this study, there was AV node dysfunction as indicated by a prolongation of the PR interval (Figure 1G). AV node dysfunction has been observed in other animal models by us: (i) in a rabbit model of pressure and volume overload-induced HF, there is an increase in the PR interval, (ii) in a rat model of pulmonary hypertension and right-sided HF there is an increase in the AH interval, Wenckebach cycle length and AV node effective and functional refractory periods measured in the isolated Langendorff perfused heart and a 50% incidence of AV block in the isolated AV node, (iii) in a rat model of ageing there are increases in the AH interval, Wenckebach cycle length and AV node effective refractory period in the isolated AV node, (iv) in a horse model of exercise training there is an increase in the PR interval and incidence of second degree AV block, and (v) in a mouse model of exercise training there are increases in the PR interval, Wenckebach cycle length, and AV node effective refractory period (Nikolaidou *et al.*, 2015; Temple *et al.*, 2016; Saeed *et al.*, 2018; Mesirca *et al.*, 2021). In the mouse model of pressure overload-induced HF in this study, the AV node dysfunction most likely was the result of ion channel remodelling in the AV node (Figure 3) and this is also true of the other animal models. In the rabbit model of pressure and volume overload-induced HF, there is a downregulation of *Hcn1*, *Cacna1d* (Cav1.3), *Gja5* (Cx40) and *Gja1* (Cx43) transcripts (Nikolaidou *et al.*, 2015). In the rat model of pulmonary hypertension and right-sided HF, there is a widespread downregulation of ion channel and related genes, e.g. *Hcn1*, *Hcn2*, *Hcn4*, *Cacna1*(Cav1.2) and *Cacna1d* (Cav1.3) (Temple *et al.*, 2016). In the rat model of ageing, there is a downregulation of HCN4, Nav1.5, RYR2 and Cx43 proteins (Saeed *et al.*, 2018). In the horse model of exercise training there is a downregulation of HCN4 and Cav1.2 protein (Mesirca *et al.*, 2021). In the mouse model of exercise training there is a downregulation of various ion channels transcripts: *Hcn2, Hcn4*, *Cacna1*(Cav1.2), *Cacna1g* (Cav3.1), *Cacna1h* (Cav3.2), *Ryr2*, and various K^+ channel and connexin transcripts; there is also a downregulation of HCN4 and Cav1.2 protein and $I_{Ca,L}$ and I_f (Mesirca *et al.*, 2021). It is concluded from the present study as well as the earlier studies that AV node dysfunction in HF and other conditions is likely to be the result of a transcriptional downregulation of key ion channels involved in AV node function.

Limitation of the study

In this study, the C57Bl6/N mouse TAC model is described as a model of HF as we did previously (Yanni *et al.*, 2020). Previous studies have supported the use of TAC in C57Bl6/N mice as a model of HF (Zi *et al.*, 2019). In this study, HF was defined based on a multitude of symptoms: reduced function (reduced ejection fraction, reduced fractional shortening), increased heart size (increased heart weight, heart weight/body weight ratio, left ventricular mass, left ventricular end diastolic diameter, and left ventricular end systolic diameter), reduced body weight, reduced heart rate, and outward signs of discomfort such as laboured breathing and lack of movement. These signs were evident in most if not all the TAC mice in this study (Figures 1 and S1). Neverthless, the outcomes of TAC were heterogeneous as shown in Figures 1 and S1. For example, the ejection fraction of all the TAC mice was lower than that of the control mice, but it still varied from 29.1 to 61.5%. The criterion for HF with preserved ejection fraction is generally an ejection fraction >50% (Pfeffer *et al.*, 2019), whereas the criterion for HF with reduced ejection fraction is <40% (Murphy *et al.*, 2020). Five of the 10 mice had an ejection fraction >40% and of these three had an ejection fraction >50%. It is possible that some mice displayed HF with a preserved ejection fraction. Hypertrophy cardiomyopathy is characterised by cardiac hypertrophy, a non-dilated left ventricle and a normal or increased ejection fraction, which is not evident in our study (Marian and Braunwald, 2017).

In this study, transcripts were measured in tissue taken from the Triangle of Koch lying between the coronary sinus, the tendon of Todaro and the tricuspid valve annulus (Li *et al.*, 2008). This will not only include the compact node made up of N (nodal) cells, but also transitional tissue made up of AN (atrio-nodal) cells, the inferior nodal extension made up of N cells, and perhaps the start of the penetrating bundle made up of NH (nodal-His) cells (Inada *et al.*, 2009). Whereas the compact node and inferior nodal extension are made up of typical nodal cells, AN and NH cells are more transitional in nature. In a previous study of the rat, we used laser-assisted microdissection to collect tissue from each of the regions and quantitative PCR (qPCR) to measure the expression of selected transcripts (Temple *et al.*, 2016). This, however, is labour intensive and time consuming and would also be more difficult in a smaller animal (the mouse rather than the rat), which is why in this study expression of transcripts was measured in Triangle of Koch biopsies as we did in a

previous study of the mouse (Mesirca *et al.*, 2021). The limitation of this is that the measured transcript expression will be determined by the expression within the different cell types in the Triangle of Koch. To estimate what is determining the measured transcript expression in the present study, five marker transcripts were compared in this study (of the Triangle of Koch) and our previous study (of the various tissues making up the AV junction). The marker transcripts are *Hcn1* and *Hcn4* (known to be highly expressed in the AV node and poorly expressed in the atrial muscle) and *Scn5a*, *Kcnj2* and *Gja1* (known to be poorly expressed in the AV node and highly expressed in the atrial muscle). The table below shows the ratio of the nodal markers (*Hcn1* or *Hcn4*) to the atrial muscle markers (*Scn5a*, *Kcnj2* and *Gja1*) in the present study and our previous study (Temple *et al.*, 2016) of the various tissues of the AV junction. The expression of the marker transcripts in the present study is unlike that of atrial muscle, but only in one instant was it like that of the compact node (*Hcn1*/*Scn5a*); in general, the expression of the marker transcripts in the present study is consistent with the expression in N and AN cells. A limitation of this analysis is that mouse tissue is being compared to rat tissue.

	Triangle of Koch (this study)	Atrial muscle	Tendon of Todaro (AN cells)	Inferior nodal extension (N cells)	Compact node (N cells)	Penetrating bundle (NH cells)	Ventricular muscle
Hcn4/Scn5a	11.58	0.22	2.36	15.25	29.33	15.92	0.10
Hcn4/Kcnj2	10.53	2.13	13.73	84.27	91.81	69.89	1.12
Hcn4/Gja1	1.46	0.18	5.00	25.80	76.24	176.60	0.16
Hcn1/Scn5a	1.84	0.02	0.11	0.77	1.02	0.09	0.00
Hcn1/Kcnj2	1.67	0.20	0.63	4.27	3.20	0.39	0.00
Hcn1/Gja1	0.23	0.02	0.23	1.31	2.66	0.99	0.00

The various signalling pathways discussed above are known to be present in various cell types including immune cells as well as in cardiomyocytes. The Hippo pathway in various cell types is known to be involved in activation of the immune system following cardiac injury (Mia and Singh, 2019). WNT signalling in immune cells plays an important role in immune cell regulation (Chae and Bothwell, 2018). CaMKII is a mediator of inflammatory processes in the heart: CaMKII signalling in the immune system is responsible for the pro-inflammatory cytokine production in macrophages, and CaMKII signalling in the heart influences the degree of the inflammatory response (Beckendorf *et al.*, 2018). The p38-MAPKα/β pathway is involved in the inflammatory response (Clerk and Sugden, 2006) and p38 MAPK overexpression in the heart has been shown to induce gene expression resulting in myocardial cell proliferation, inflammation, and fibrosis (Tenhunen *et al.*, 2006). Therefore, the changes in the signalling pathway transcripts observed in this study may be occurring in for example immune cells rather than cardiomyocytes – further study is required to clarify this.

A final comment

Although hypothesis-driven research has many advantages, it blinkers the investigator to the complexity of biological systems potentially leading to erroneous conclusions, whereas omics technologies and data-driven discovery does not constrain the view of the investigator in this way (Boyett and Lundby, 2020). The current study is a good example of this: it has revealed that HF likely causes AV node dysfunction not as a result of a change in a single or small number of molecules. Instead, the AV node dysfunction likely involves widespread changes in many different cellular systems. This has implications for the development of new treatments.

ADDITIONAL SUPPLEMENTARY FILES

All transcripts.xlsx

For all transcripts detected, this file lists: gene name; mean, SEM and n for both control and HF; ratio of the HF mean to control mean expressed as a percentage; P value; Benjamini-Hochberg-adjusted P value.

Canonical pathways.xlsx

Ingenuity pathway analysis was used to identify canonical pathways significantly associated with cytokines, protein kinases and transcription factors significantly altered in HF. The file shows

the canonical pathways, the P value of the association, and the cytokines, protein kinases and transcription factors involved. The pathways have been organised into different groups. The highlighted signalling pathways are the ones discussed in depth and shown in Figure 8.

SUPPLEMENTARY REFERENCES

Adapala, V. J., Adedokun, S. A., Considine, R. V., and Ajuwon, K. M. (2012). Acute inflammation plays a limited role in the regulation of adipose tissue COL1A1 protein abundance. *Journal of Nutritional Biochemistry* 23, 567-572. 10.1016/j.jnutbio.2011.02.013

Baruscotti, M., Bucchi, A., Viscomi, C., Mandelli, G., Consalez, G., Gnecchi-Rusconi, T., et al. (2011). Deep bradycardia and heart block caused by inducible cardiac-specific knockout of the pacemaker channel gene Hcn4. *Proceedings of the National Academy of Sciences of the United States of America* 108, 1705-10. 10.1073/pnas.1010122108

Beckendorf, J., van den Hoogenhof, M. M. G., and Backs, J. (2018). Physiological and unappreciated roles of CaMKII in the heart. *Basic Research in Cardiology* 113, 29. 10.1007/s00395-018-0688-8

Bernstein, D., Fajardo, G., and Zhao, M. (2011). The role of β-adrenergic receptors in heart failure: differential regulation of cardiotoxicity and cardioprotection. *Progress in Pediatric Cardiology* 31, 35-38. 10.1016/j.ppedcard.2010.11.007

Boyett, M., and Lundby, A. (2020). A new window onto the pacemaker of the heart, the sinus node, provided by quantitative proteomics and single-nucleus transcriptomics. *Journal of Cellular Immunology* 2, 38-41.

Bui, A. L., Horwich, T. B., and Fonarow, G. C. (2011). Epidemiology and risk profile of heart failure. *Nature Reviews Cardiology* 8, 30-41. 10.1038/nrcardio.2010.165

Chae, W. J., and Bothwell, A. L. M. (2018). Canonical and non-canonical Wnt signaling in immune cells. *Trends in Immunology* 39, 830-847. 10.1016/j.it.2018.08.006

Chen, X., Li, Y., Luo, J., and Hou, N. (2020). Molecular mechanism of Hippo–YAP1/TAZ pathway in heart development, disease, and regeneration. *Frontiers in Physiology* 11. 10.3389/fphys.2020.00389

Chiang, D., Y., Alsina, K., M., Corradini, E., Fitzpatrick, M., Ni, L., Lahiri, S., K., et al. (2018). Rearrangement of the protein phosphatase 1 interactome during heart failure progression. *Circulation* 138, 1569-1581. 10.1161/CIRCULATIONAHA.118.034361

Chiang, D. Y., Heck, A. J. R., Dobrev, D., and Wehrens, X. H. T. (2016). Regulating the regulator: insights into the cardiac protein phosphatase 1 interactome. *Journal of Molecular and Cellular Cardiology* 101, 165-172. 10.1016/j.yjmcc.2016.09.009

Clausen, M. V., Hilbers, F., and Poulsen, H. (2017). The structure and function of the Na,K-ATPase isoforms in health and disease. *Frontiers in Physiology* 8, 371. 10.3389/fphys.2017.00371

Clerk, A., and Sugden, P. H. (2006). Inflame my heart (by p38-MAPK). *Circulation Research* 99, 455-458. 10.1161/01.RES.0000241053.89089.c3

Cordero, P., Parikh, V. N., Chin, E. T., Erbilgin, A., Gloudemans, M. J., Shang, C., et al. (2019). Pathologic gene network rewiring implicates PPP1R3A as a central regulator in pressure overload heart failure. *Nature Communications* 10, 2760. 10.1038/s41467-019-10591-5

DeGrande, S. T., Little, S. C., Nixon, D. J., Wright, P., Snyder, J., Dun, W., et al. (2013). Molecular mechanisms underlying cardiac protein phosphatase 2A regulation in heart. *Journal of Biological Chemistry* 288, 1032-1046. 10.1074/jbc.M112.426957

Dirkx, E., da Costa Martins, P. A., and De Windt, L. J. (2013). Regulation of fetal gene expression in heart failure. *Biochimica et Biophysica Acta* 1832, 2414-2424. 10.1016/j.bbadis.2013.07.023

Dong, R., Zhang, M., Hu, Q., Zheng, S., Soh, A., Zheng, Y., et al. (2018). Galectin-3 as a novel biomarker for disease diagnosis and a target for therapy. *International Journal of Molecular Medicine* 41, 599-614. 10.3892/ijmm.2017.3311

Foulquier, S., Daskalopoulos, E. P., Lluri, G., Hermans, K. C. M., Deb, A., and Blankesteijn, W. M. (2018). WNT signaling in cardiac and vascular disease. *Pharmacological Reviews* 70, 68-141. 10.1124/pr.117.013896

Goodfellow, S. J., and Zomerdijk, J. C. (2013). Basic mechanisms in RNA polymerase I transcription of the ribosomal RNA genes. *Subcellular Biochemistry* 61, 211-36. 10.1007/978-94-007-4525-4_10

Han, Y. S., Arroyo, J., and Ogut, O. (2013). Human heart failure is accompanied by altered protein kinase A subunit expression and post-translational state. *Archives of Biochemistry and Biophysics* 538, 25-33. 10.1016/j.abb.2013.08.002

Hanna, A., and Frangogiannis, N. G. (2019). The role of the TGF-β superfamily in myocardial infarction. *Frontiers in Cardiovascular Medicine* 6, 140-140. 10.3389/fcvm.2019.00140

Hori, M., and Yamaguchi, O. (2013). Is tumor necrosis factor-α friend or foe for chronic heart failure? *Circulation Research* 113, 492-494. 10.1161/CIRCRESAHA.113.302024

Huang, Z. M., Prasad, C., Britton, F. C., Ye, L. L., Hatton, W. J., and Duan, D. (2009). Functional role of CLC-2 chloride inward rectifier channels in cardiac sinoatrial nodal pacemaker cells. *Journal of Molecular and Cellular Cardiology* 47, 121-32. 10.1016/j.yjmcc.2009.04.008

Ikeda, S., Mizushima, W., Sciarretta, S., Abdellatif, M., Zhai, P., Mukai, R., et al. (2019). Hippo deficiency leads to cardiac dysfunction accompanied by cardiomyocyte dedifferentiation during pressure overload. *Circulation Research* 124, 292-305. 10.1161/CIRCRESAHA.118.314048

Imanaka-Yoshida, K., Tawara, I., and Yoshida, T. (2020). Tenascin-C in cardiac disease: a sophisticated controller of inflammation, repair, and fibrosis. *American Journal of Physiology-Cell Physiology* 319, C781-C796. 10.1152/ajpcell.00353.2020

Inada, S., Hancox, J. C., Zhang, H., and Boyett, M. R. (2009). One-dimensional mathematical model of the atrioventricular node including atrio-nodal, nodal, and nodal-his cells. *Biophysical Journal* 97, 2117-2127.

Jensen, B. C., O'Connell, T. D., and Simpson, P. C. (2014). Alpha-1–adrenergic receptors in heart failure: the adaptive arm of the cardiac response to chronic catecholamine stimulation. *Journal of Cardiovascular Pharmacology* 63.

Jeon, S. B., Yoon, H. J., Chang, C. Y., Koh, H. S., Jeon, S. H., and Park, E. J. (2010). Galectin-3 exerts cytokine-like regulatory actions through the JAK-STAT pathway. *Journal of Immunology* 185, 7037-46. 10.4049/jimmunol.1000154

Kapur, N. K. (2011). Transforming growth factor-β. Governing the transition from inflammation to fibrosis in heart failure with preserved left ventricular function. *Circulation: Heart Failure* 4, 5-7. doi:10.1161/CIRCHEARTFAILURE.110.960054

Li, J., Greener, I. D., Inada, S., Nikolski, V. P., Yamamoto, M., Hancox, J. C., et al. (2008). Computer three-dimensional reconstruction of the atrioventricular node. *Circulation Research* 102, 975-985.

Liang, D., Xue, Z., Xue, J., Xie, D., Xiong, K., Zhou, H., et al. (2021). Sinoatrial node pacemaker cells share dominant biological properties with glutamatergic neurons. *Protein Cell*. 10.1007/s13238-020-00820-9

Lipson, K. E., Wong, C., Teng, Y., and Spong, S. (2012). CTGF is a central mediator of tissue remodeling and fibrosis and its inhibition can reverse the process of fibrosis. *Fibrogenesis & Tissue Repair* 5, S24. 10.1186/1755-1536-5-S1-S24

Lopez-Castejon, G., and Brough, D. (2011). Understanding the mechanism of IL-1β secretion. *Cytokine Growth Factor Rev* 22, 189-195. 10.1016/j.cytogfr.2011.10.001

Lubbers, E. R., and Mohler, P. J. (2016). Roles and regulation of protein phosphatase 2A (PP2A) in the heart. *Journal of Molecular and Cellular Cardiology* 101, 127-133. 10.1016/j.yjmcc.2016.11.003

Malekar, P., Hagenmueller, M., Anyanwu, A., Buss, S., Streit Marcus, R., Weiss Celine, S., et al. (2010). Wnt signaling is critical for maladaptive cardiac hypertrophy and accelerates myocardial remodeling. *Hypertension* 55, 939-945. 10.1161/HYPERTENSIONAHA.109.141127

Man, J., Barnett, P., and Christoffels, V. M. (2018). Structure and function of the Nppa-Nppb cluster locus during heart development and disease. *Cellular and Molecular Life Sciences* 75, 1435-1444. 10.1007/s00018-017-2737-0

Marian, A. J., and Braunwald, E. (2017). Hypertrophic cardiomyopathy. *Circulation Research* 121, 749-770. 10.1161/CIRCRESAHA.117.311059

McKinsey, T. A. (2011). Targeting inflammation in heart failure with histone deacetylase inhibitors. *Molecular Medicine* 17, 434-41. 10.2119/molmed.2011.00022

McNally, E. M., and Sparano, D. (2011). Mechanisms and management of the heart in myotonic dystrophy. *Heart* 97, 1094-1100. 10.1136/hrt.2010.214197

Megías, M., Molist, P., and Pombal, M. A. (2019). Animal tissues. Atlas of Plant and Animal Histology. http://mmegias.webs.uvigo.es/index.html.
Mesirca, P., Nakao, S., Nissen, S. D., Forte, G., Anderson, C., Trussell, T., et al. (2021). Intrinsic electrical remodeling underlies atrioventricular block in athletes. *Circulation Research* 129, e1-e20. 10.1161/CIRCRESAHA.119.316386
Mia, M. M., and Singh, M. K. (2019). The Hippo signaling pathway in cardiac development and diseases. *Frontiers in Cell and Developmental Biology* 7, 211.
Mohamed, T. M. A., Abou-Leisa, R., Stafford, N., Maqsood, A., Zi, M., Prehar, S., et al. (2016). The plasma membrane calcium ATPase 4 signalling in cardiac fibroblasts mediates cardiomyocyte hypertrophy. *Nature Communications* 7, 11074. 10.1038/ncomms11074
Murphy, S. P., Ibrahim, N. E., and Januzzi, J. L., Jr. (2020). Heart failure with reduced ejection fraction: a review. *JAMA* 324, 488-504. 10.1001/jama.2020.10262
Nikolaidou, T., Cai, X., Stephenson, R. S., Yanni, J., Lowe, T., Atkinson, A. J., et al. (2015). Congestive heart failure leads to prolongation of the PR Interval and atrioventricular junction enlargement and ion channel remodelling in the rabbit. *PLoS One* 10, e0141452. 10.1371/journal.pone.0141452
Novoyatleva, T., Diehl, F., van Amerongen, M. J., Patra, C., Ferrazzi, F., Bellazzi, R., et al. (2009). TWEAK is a positive regulator of cardiomyocyte proliferation. *Cardiovascular Research* 85, 681-690. 10.1093/cvr/cvp360
O'Connell, T. D., Jensen, B. C., Baker, A. J., and Simpson, P. C. (2013). Cardiac alpha$_1$-adrenergic receptors: novel aspects of expression, signaling mechanisms, physiologic function, and clinical importance. *Pharmacological Reviews* 66, 308-333. 10.1124/pr.112.007203
Pfeffer, M. A., Shah, A. M., and Borlaug, B. A. (2019). Heart failure with preserved ejection fraction in perspective. *Circulation Research* 124, 1598-1617. 10.1161/CIRCRESAHA.119.313572
Richter, W., Xie, M., Scheitrum, C., Krall, J., Movsesian, M. A., and Conti, M. (2011). Conserved expression and functions of PDE4 in rodent and human heart. *Basic Research in Cardiology* 106, 249-262. 10.1007/s00395-010-0138-8
Saeed, Y., Temple, I. P., Borbas, Z., Atkinson, A., Yanni, J., Maczewski, M., et al. (2018). Structural and functional remodeling of the atrioventricular node with aging in rats: The role of hyperpolarization-activated cyclic nucleotide-gated and ryanodine 2 channels. *Heart Rhythm* 15, 752-760. 10.1016/j.hrthm.2017.12.027
Santamaria, S., and de Groot, R. (2020). ADAMTS proteases in cardiovascular physiology and disease. *Open Biology* 10, 200333. 10.1098/rsob.200333
Schilders, G., Raijmakers, R., Raats, J. M. H., and Pruijn, G. J. M. (2005). MPP6 is an exosome-associated RNA-binding protein involved in 5.8S rRNA maturation. *Nucleic Acids Research* 33, 6795-6804. 10.1093/nar/gki982
Shen, G.-M., Zhang, F.-L., Liu, X.-L., and Zhang, J.-W. (2010). Hypoxia-inducible factor 1-mediated regulation of PPP1R3C promotes glycogen accumulation in human MCF-7 cells under hypoxia. *FEBS Letters* 584, 4366-4372. 10.1016/j.febslet.2010.09.040
Simpson, L. J., Reader, J. S., and Tzima, E. (2020). Mechanical regulation of protein translation in the cardiovascular system. *Frontiers in Cell and Developmental Biology* 8. 10.3389/fcell.2020.00034
Swaminathan, P. D., Purohit, A., Soni, S., Voigt, N., Singh, M. V., Glukhov, A. V., et al. (2011). Oxidized CaMKII causes cardiac sinus node dysfunction in mice. *Journal of Clinical Investigation* 121, 3277-88. 10.1172/JCI57833
Temple, I. P., Logantha, S. J. R. J., Absi, M., Zhang, Y., Pervolaraki, E., Yanni, J., et al. (2016). Atrioventricular node dysfunction and ion channel transcriptome in pulmonary hypertension. *Circulation. Arrhythmia and Electrophysiology* 9, e003432. 10.1161/circep.115.003432
Tenhunen, O., Rysä, J., Ilves, M., Soini, Y., Ruskoaho, H., and Leskinen, H. (2006). Identification of cell cycle regulatory and inflammatory genes as predominant targets of p38 mitogen-activated protein kinase in the heart. *Circulation Research* 99, 485-493. 10.1161/01.RES.0000238387.85144.92
Vainio, L. E., Szabó, Z., Lin, R., Ulvila, J., Yrjölä, R., Alakoski, T., et al. (2019). Connective tissue growth factor inhibition enhances cardiac repair and limits fibrosis after myocardial infarction. *JACC: Basic to Translational Science* 4, 83-94. 10.1016/j.jacbts.2018.10.007

Van Tassell, B. W., Raleigh, J. M. V., and Abbate, A. (2015). Targeting interleukin-1 in heart failure and inflammatory heart disease. *Current Heart Failure Reports* 12, 33-41. 10.1007/s11897-014-0231-7

Wang, K., Meng, X., and Guo, Z. (2021a). Elastin structure, synthesis, regulatory mechanism and relationship with cardiovascular diseases. *Frontiers in Cell and Developmental Biology* 9.

Wang, Y., Anderson, C., Dobrzynski, H., Hart, G., D'Souza, A., and Boyett, M. R. (2021b). RNAseq shows an all-pervasive circadian rhythm in the transcriptome of the pacemaker of the heart, the sinus node. *Scientific Reports* 11, 3565. 10.1038/s41598-021-82202-7

Yanni, J., D'Souza, A., Wang, Y., Li, N., Hansen, B. J., Zakharkin, S. O., et al. (2020). Silencing miR-370-3p rescues funny current and sinus node function in heart failure. *Scientific Reports* 10, 11279. 10.1038/s41598-020-67790-0

Yousefi, F., Shabaninejad, Z., Vakili, S., Derakhshan, M., Movahedpour, A., Dabiri, H., et al. (2020). TGF-β and WNT signaling pathways in cardiac fibrosis: non-coding RNAs come into focus. *Cell Communication and Signaling* 18, 87. 10.1186/s12964-020-00555-4

Zhang, T., Maier, L. S., Dalton, N. D., Miyamoto, S., Ross, J., Bers, D. M., et al. (2003). The δC isoform of CaMKII Is activated in cardiac hypertrophy and induces dilated cardiomyopathy and heart failure. *Circulation Research* 92, 912-919. 10.1161/01.RES.0000069686.31472.C5

Zhao, Y., Wang, C., Wang, C., Hong, X., Miao, J., Liao, Y., et al. (2018). An essential role for Wnt/β-catenin signaling in mediating hypertensive heart disease. *Scientific Reports* 8, 8996. 10.1038/s41598-018-27064-2

Zhou, Q., Liu, M., Xia, X., Gong, T., Feng, J., Liu, W., et al. (2017). A mouse tissue transcription factor atlas. *Nature Communications* 8, 15089. 10.1038/ncomms15089

Zi, M., Stafford, N., Prehar, S., Baudoin, F., Oceandy, D., Wang, X., et al. (2019). Cardiac hypertrophy or failure? - A systematic evaluation of the transverse aortic constriction model in C57BL/6NTac and C57BL/6J substrains. *Current Research in Physiology* 1, 1-10. 10.1016/j.crphys.2019.10.001

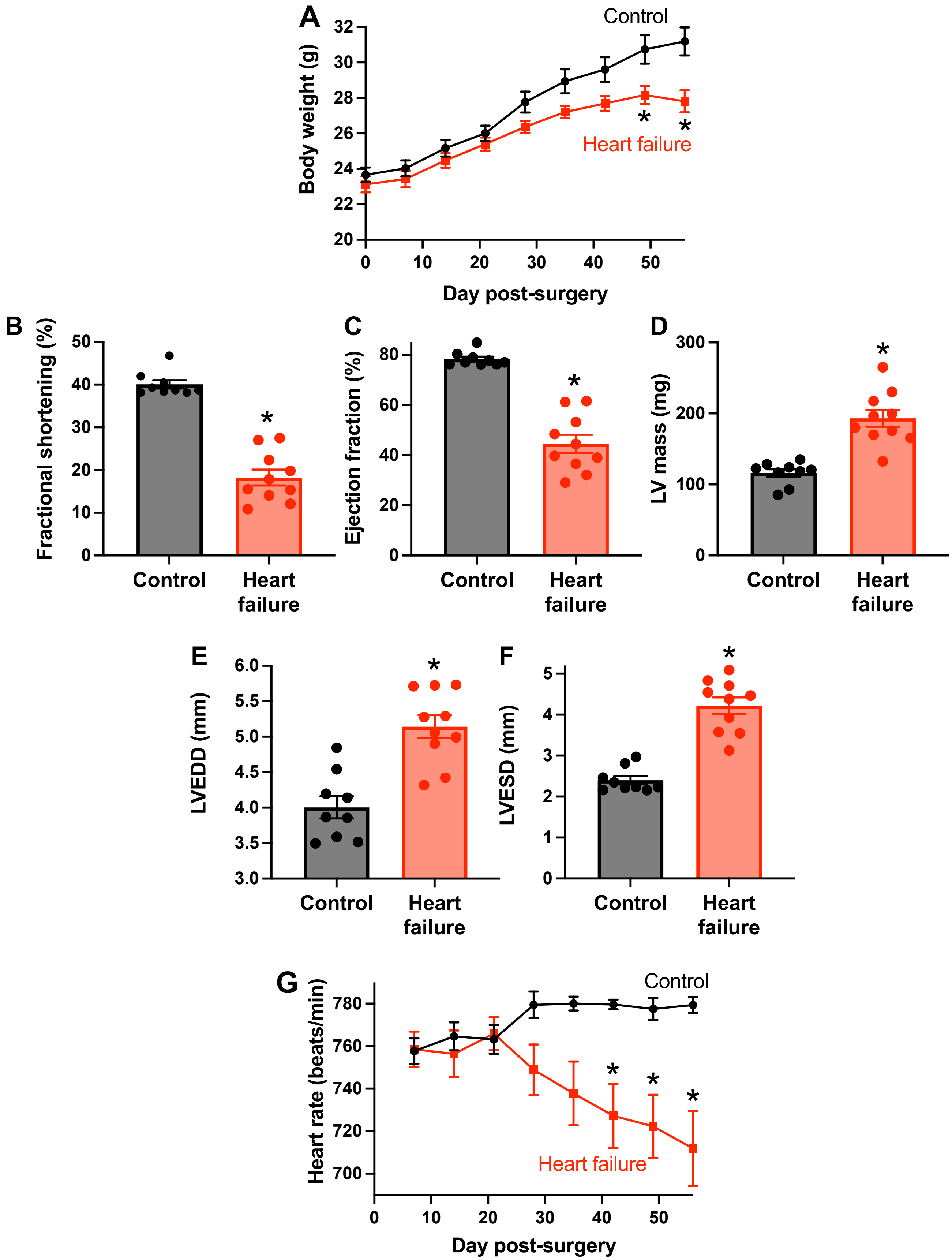

Figure S1. Further characterisation of the mouse TAC model of HF. A, mean (±SEM) body weight of control (n=9; subject to sham surgery) and HF (n=11; subject to TAC surgery) mice in the days following surgery. **B-F**, mean (+SEM and individual data points) fractional shortening (**B**), ejection fraction (**C**), left ventricular (LV) mass (**D**), left ventricular end diastolic diameter (LVEDD; **E**) and left ventricular end systolic diameter (LVESD; **F**) at the end of the experiment in the control and HF mice (n=9 and 10). **G**, mean (±SEM) heart rate of control (n=9) and HF (n=11) mice in the days following surgery. *P<0.05.

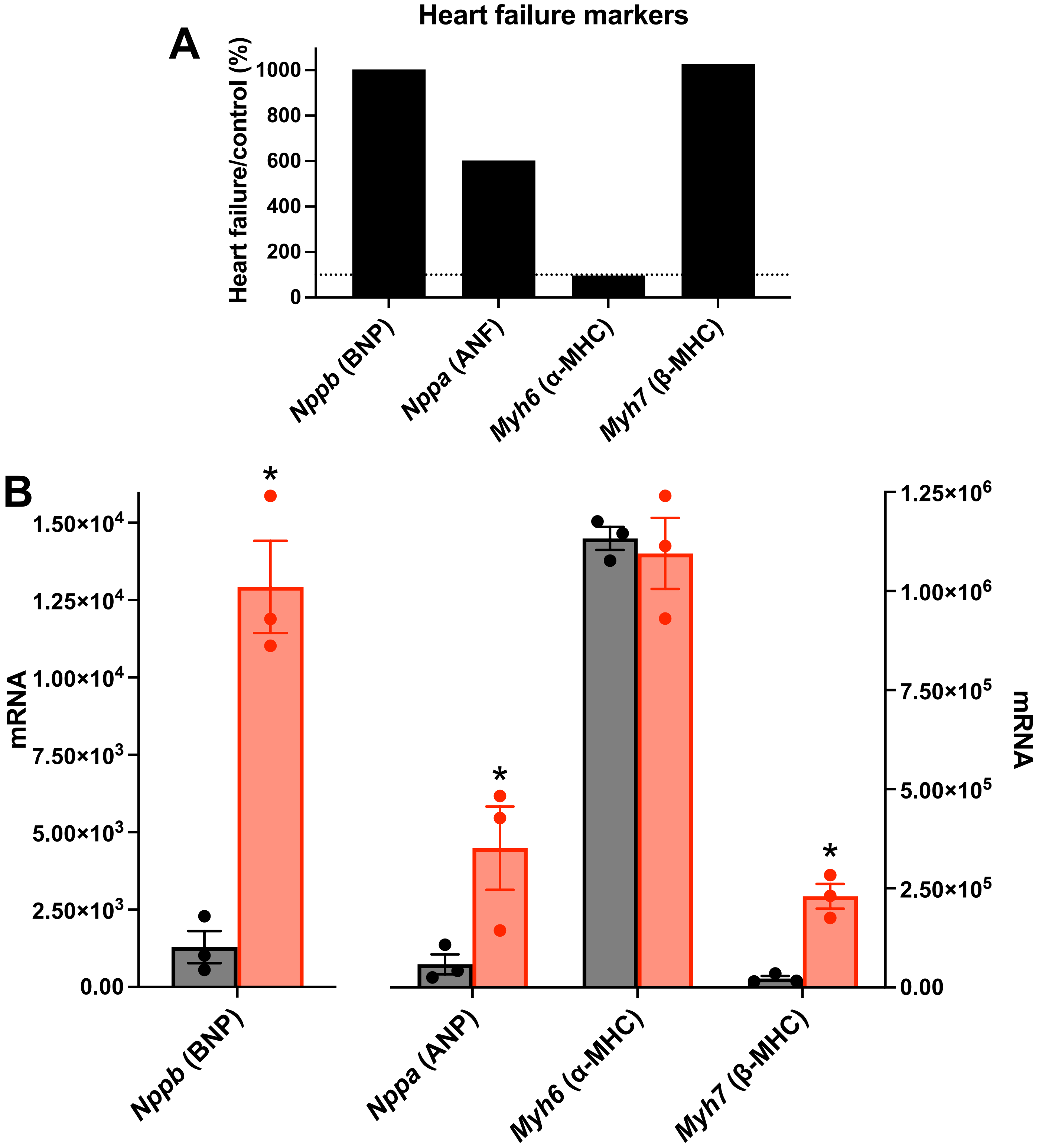

Figure S2. Expected changes in HF markers in the AV node in HF. A, expression of HF markers (transcripts) in HF mice as a percentage of that in control mice. Black dotted line corresponds to 100%. **B**, mean (+SEM) expression (and individual data points) for HF markers (transcripts) in control (black bars) and HF (red bars) mice. *P<0.05.

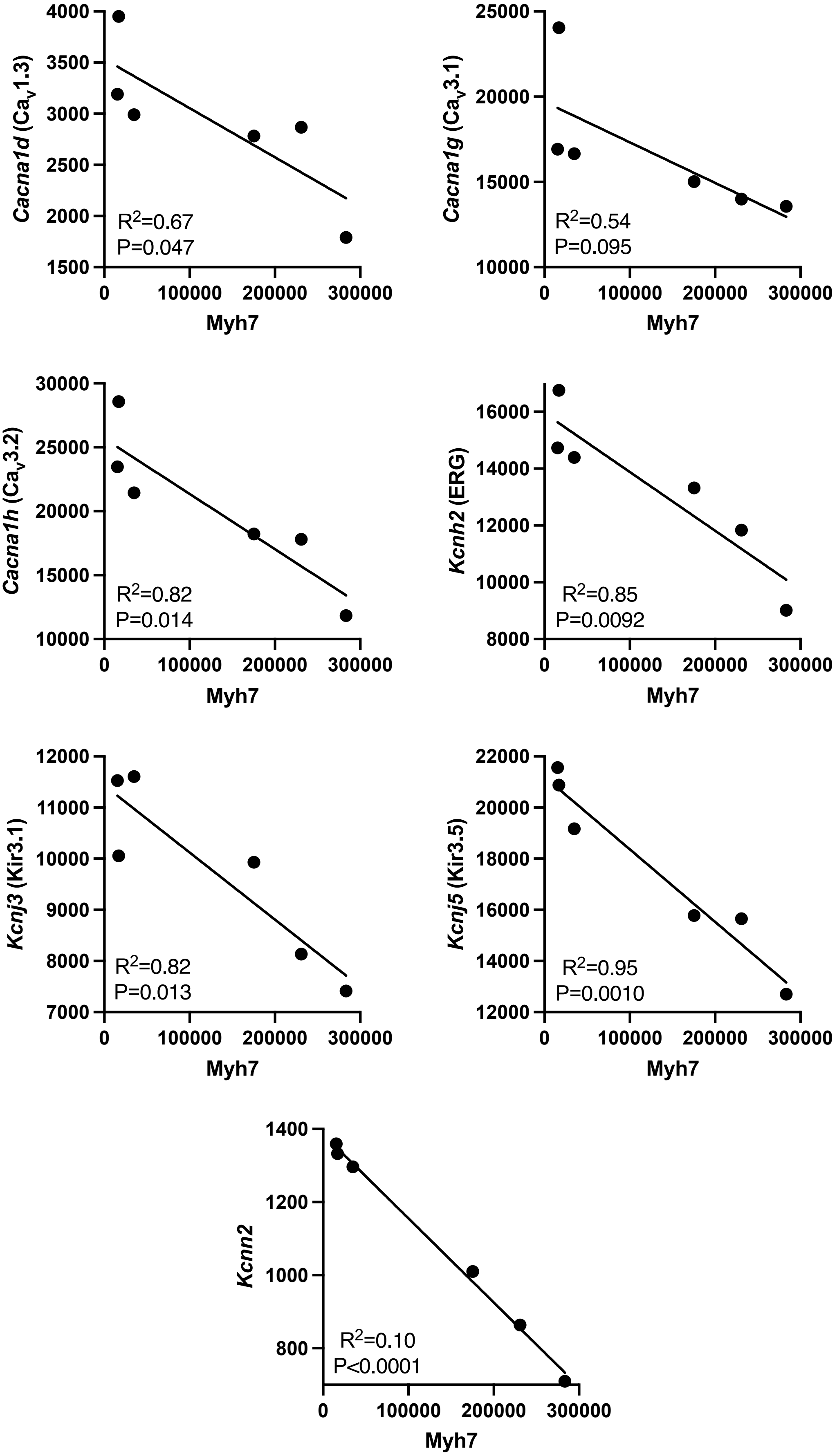

Figure S3. Correlation between key ion channel transcripts and the HF marker, Myh7. Each point corresponds to one of six pooled samples (three control and three HF samples). Each pooled sample is made of AV node biopsies from three mice. The data have been fitted with a straight line and the R^2 value and the P value (of a slope of zero) are shown. In each case the control samples are clustered near the X-axis origin.

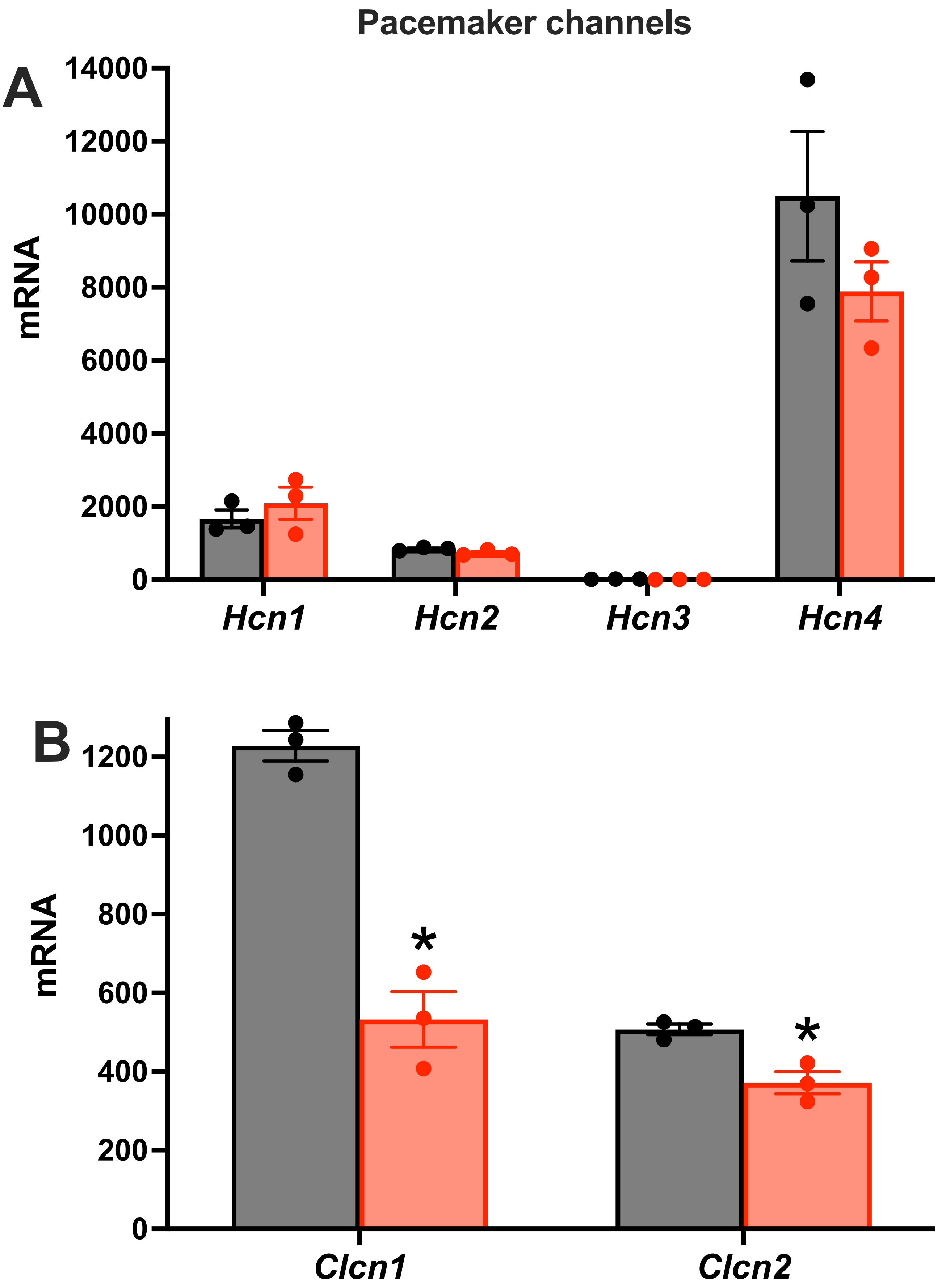

Figure S4. Few changes in pacemaker channel transcripts in the AV node in HF. A and **B**, mean (+SEM) expression (and individual data points) for *Hcn* (**A**) and Cl^- channel (**B**) transcripts in control (black bars) and HF (red bars) mice. *P<0.05.

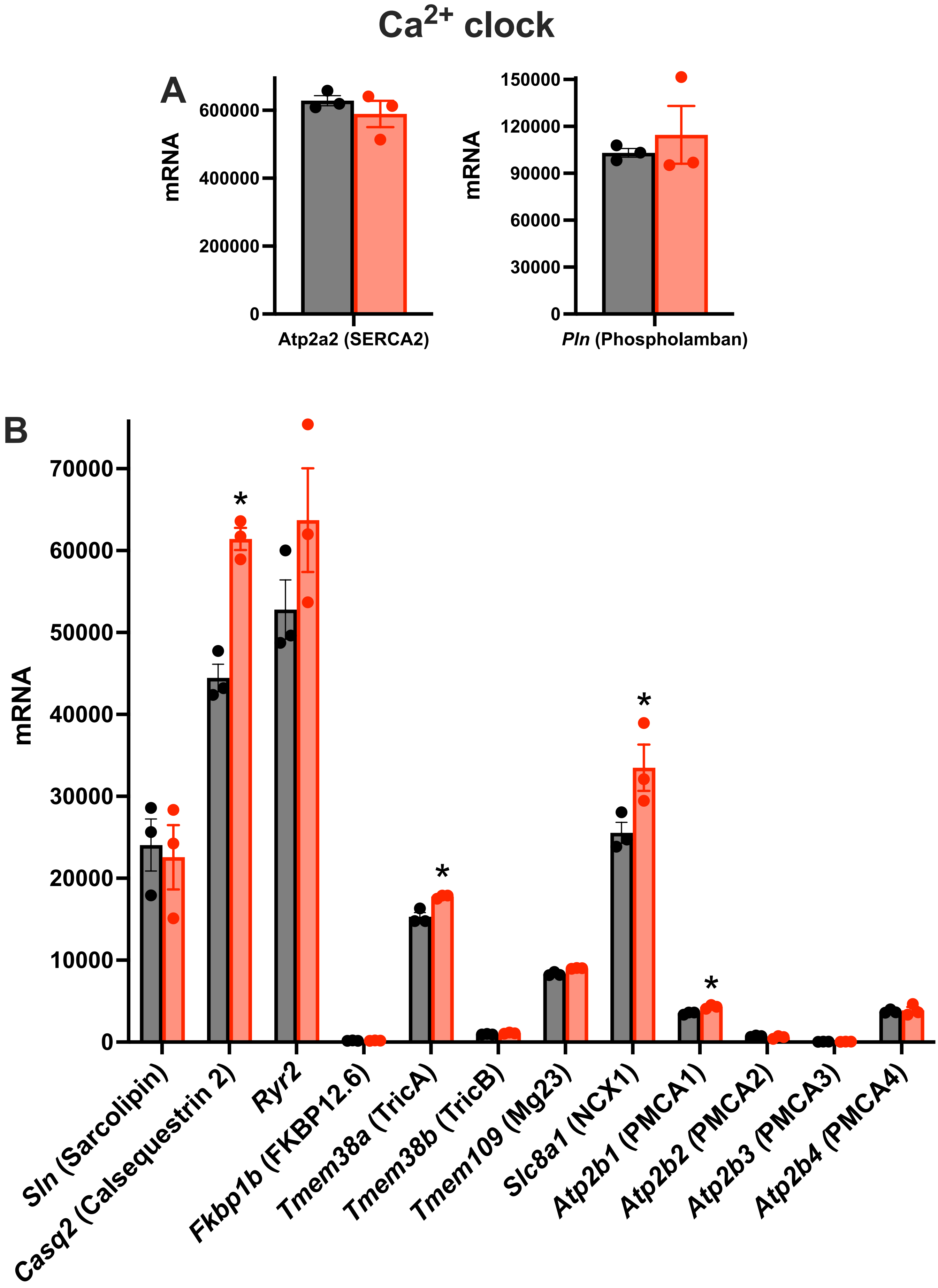

Figure S5. Ca^{2+} clock transcripts in the AV node in HF. A and **B**, mean (+SEM) expression (and individual data points) for highly expressed (**A**) and more poorly expressed (**B**) Ca^{2+} clock transcripts in control (black bars) and HF (red bars) mice. *P<0.05.

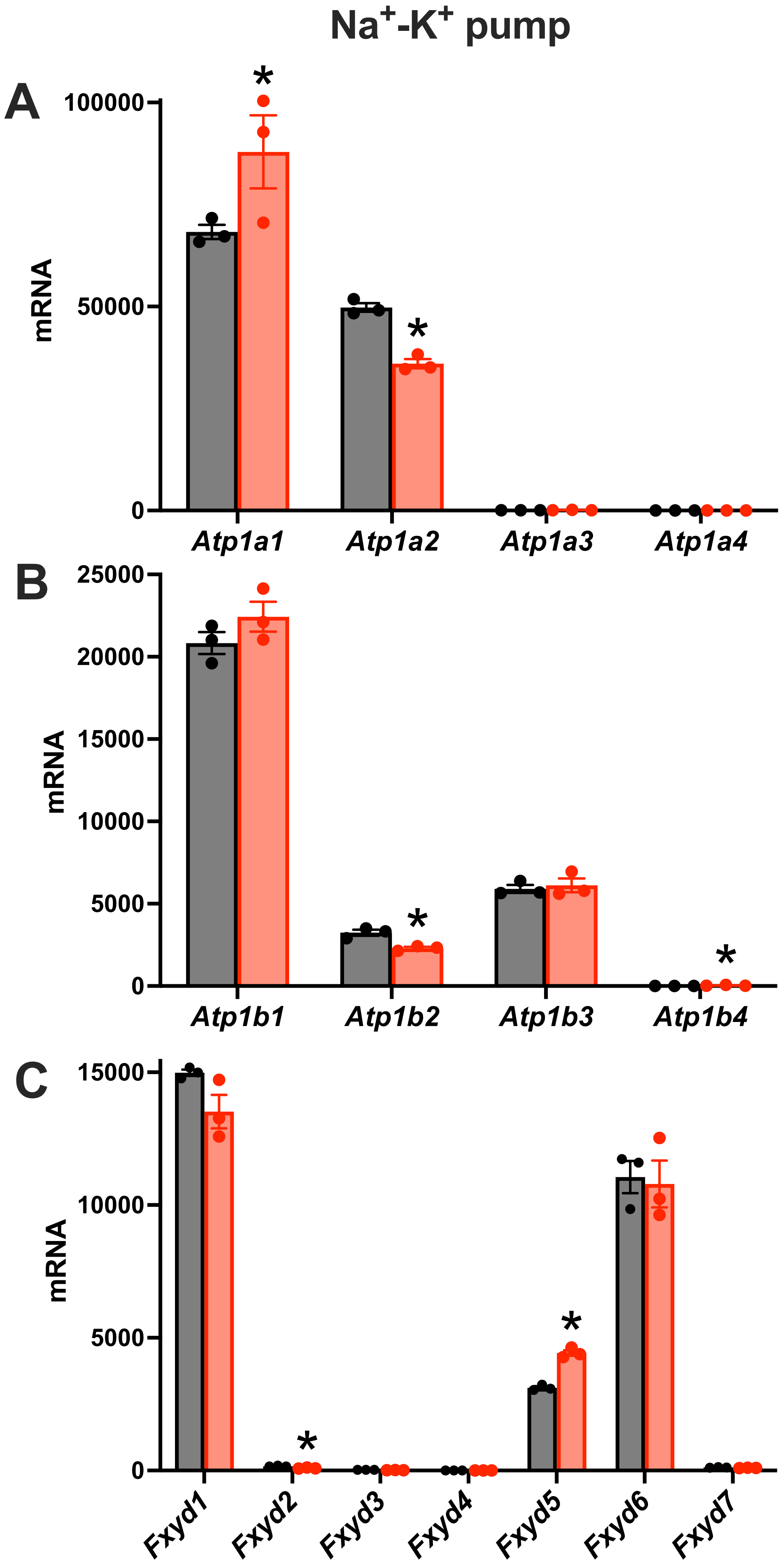

Figure S6. Subtle changes in Na$^+$-K$^+$ pump transcripts in the AV node in HF. A-C, mean (+SEM) expression (and individual data points) for Na$^+$-K$^+$ pump α subunits (**A**), Na$^+$-K$^+$ pump β subunits (**B**) and Na$^+$-K$^+$ pump regulatory molecules (**C**) in control (black bars) and HF (red bars) mice. *P<0.05.

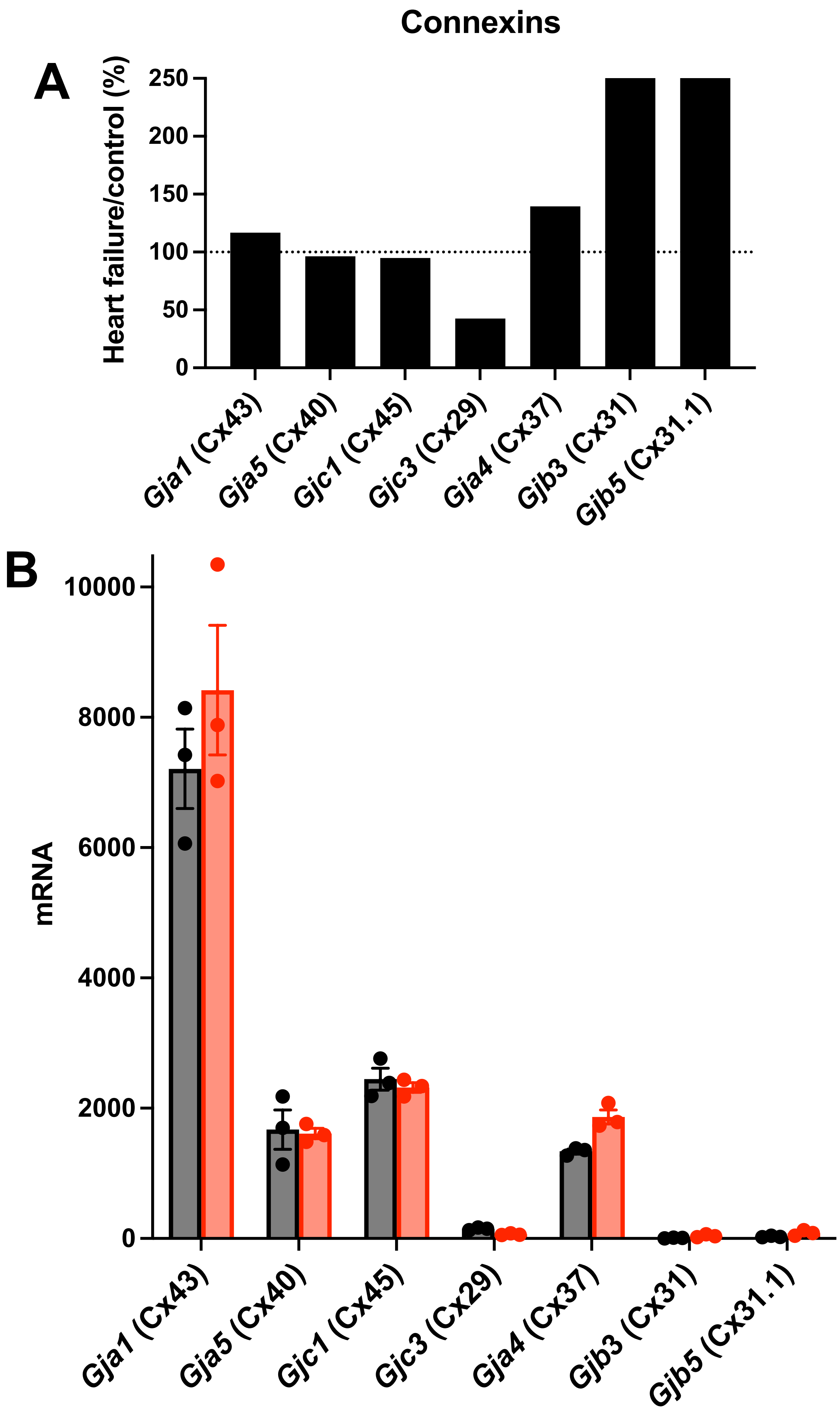

Figure S7. No changes in gap junction transcripts in the AV node in HF. A, expression of connexin transcripts in HF mice as a percentage of that in control mice. Black dotted line corresponds to 100%. **B**, mean (+SEM) expression (and individual data points) for connexin transcripts in control (black bars) and HF (red bars) mice. No significant differences observed.

Glucose and fatty acid transporters

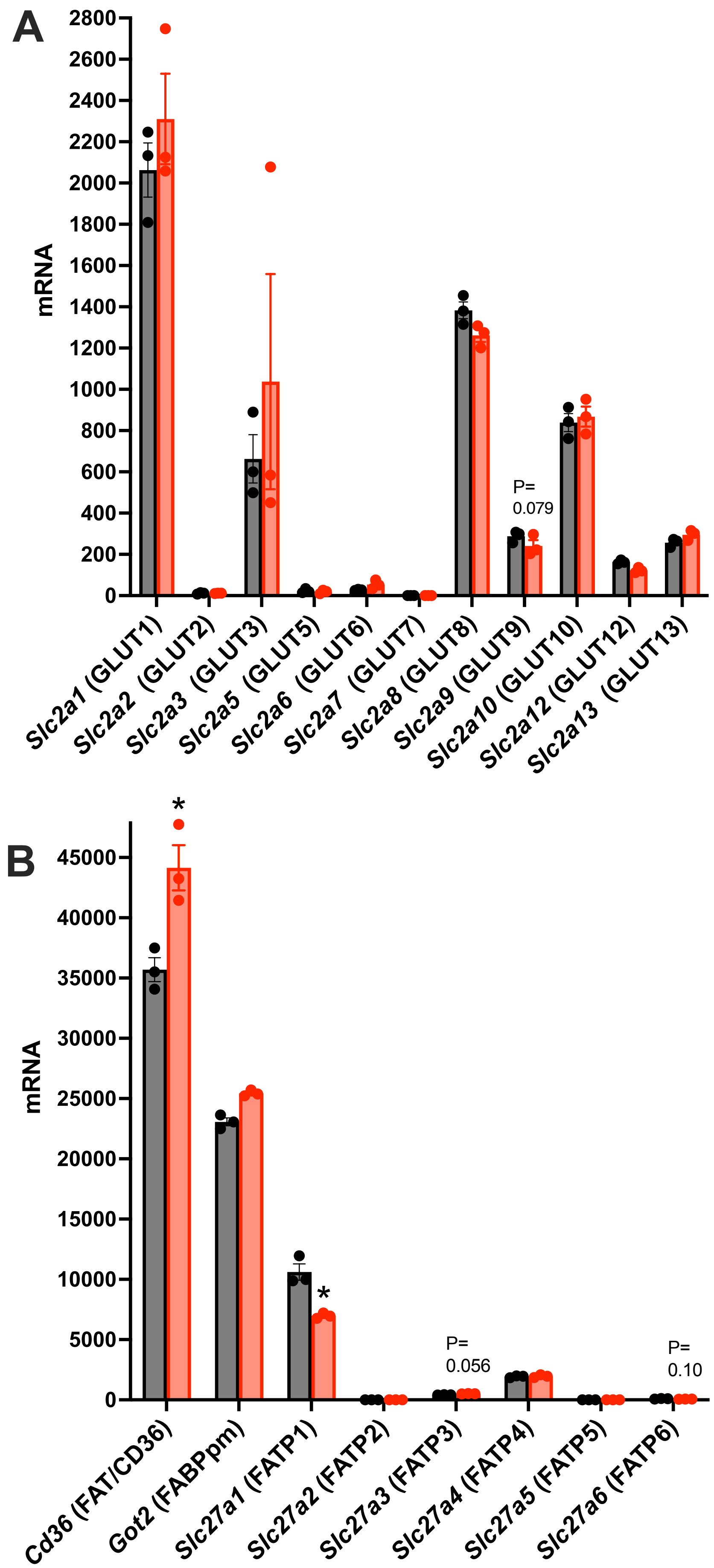

Figure S8. Changes in glucose (A) and fatty acid (B) transporter transcripts in the AV node in HF. Mean (+SEM) expression (and individual data points) for control (black bars) and HF (red bars) mice shown. *P<0.05.

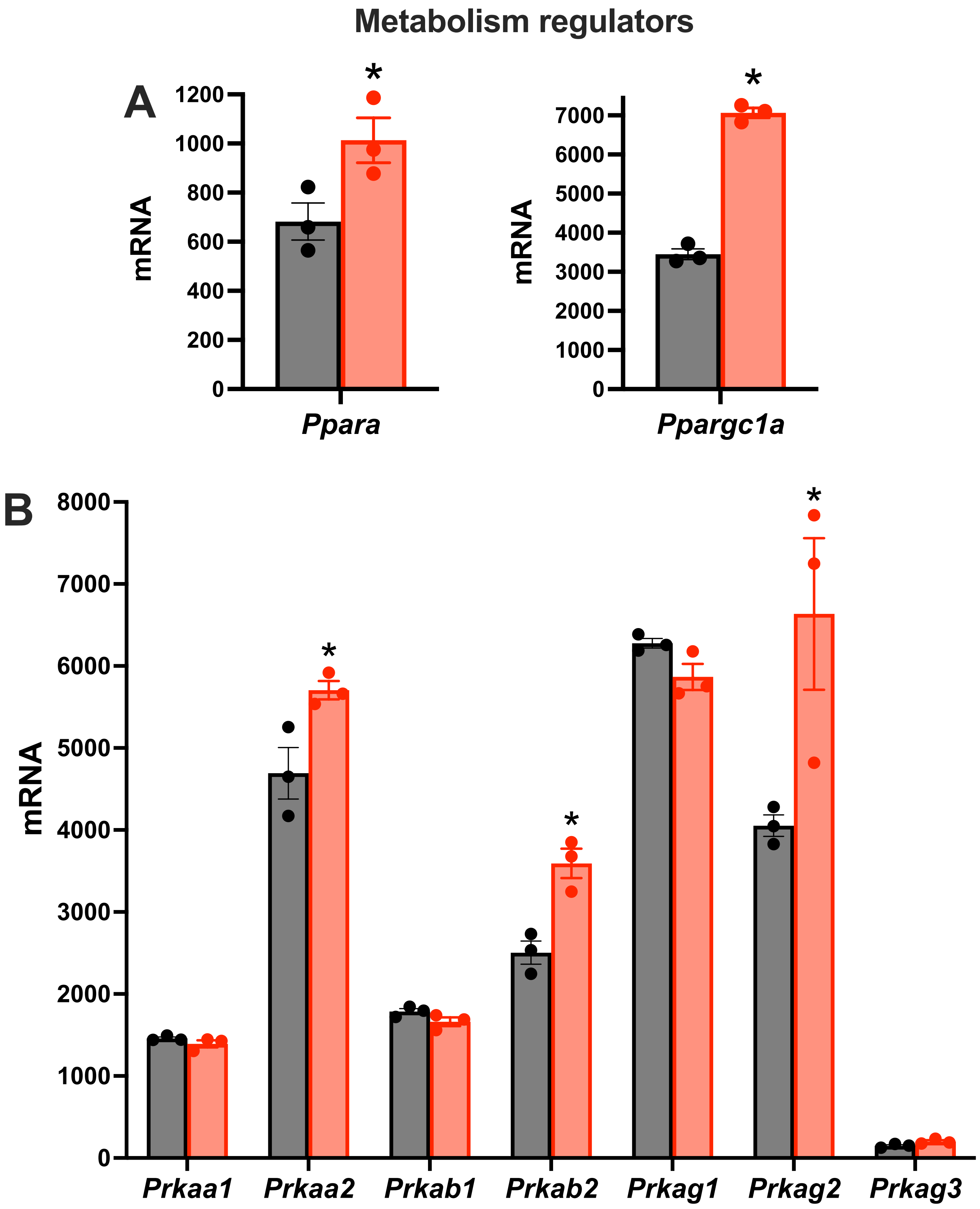

Figure S9. Changes in peroxisome proliferator-activated receptor alpha (*Ppara*) and peroxisome proliferator-activated receptor gamma coactivator 1-alpha (*Ppargc1a*) transcripts (A) and AMP-activated protein kinase subunit transcripts (B) in the AV node in HF. Mean (+SEM) expression (and individual data points) for control (black bars) and HF (red bars) mice shown. *P<0.05.

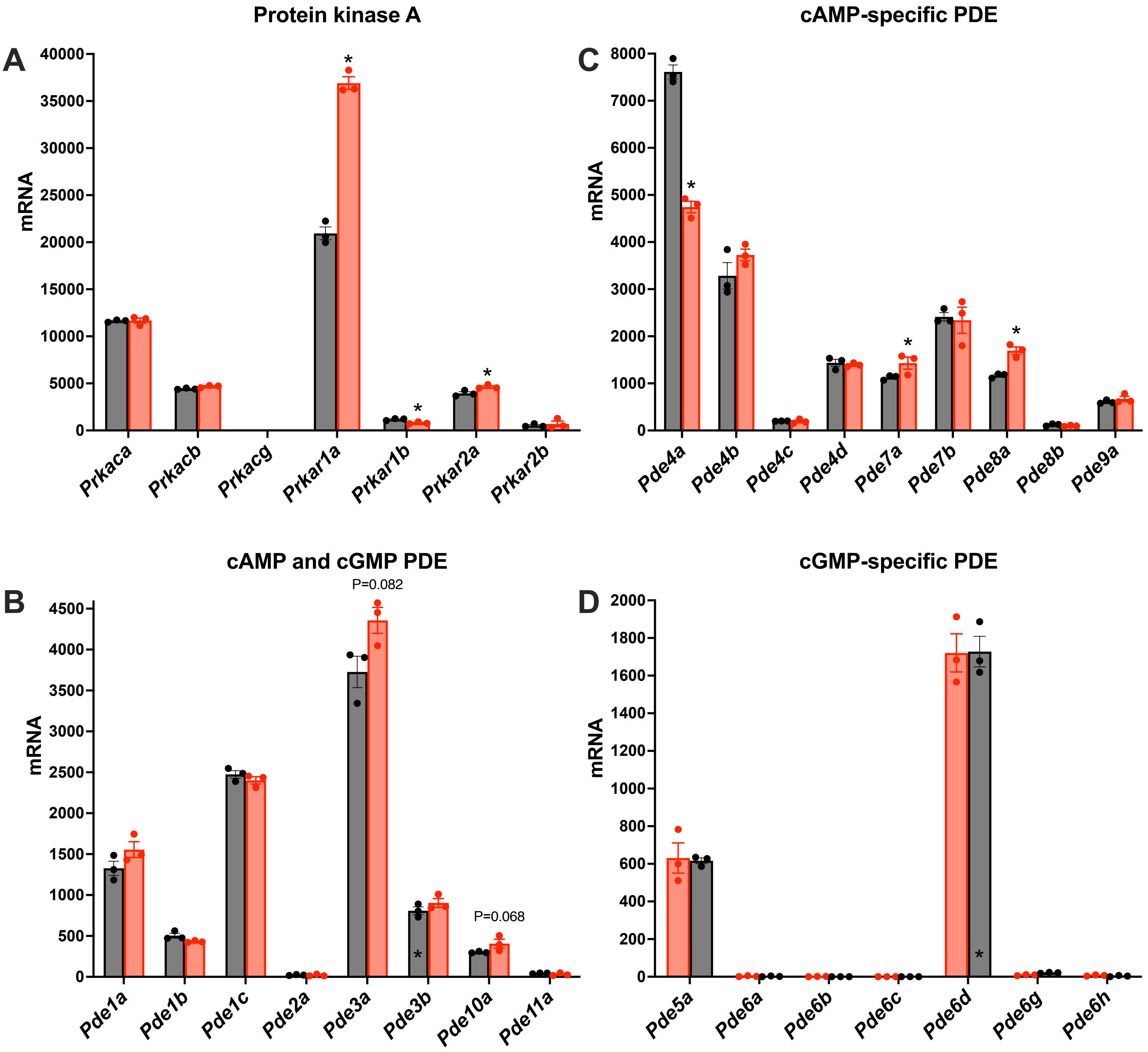

Figure S10. Changes in protein kinase A (A) and phosphodiesterase (B-D) transcripts in the AV node in HF. Mean (+SEM) expression (and individual data points) for control (black bars) and HF (red bars) mice shown. *P<0.05.

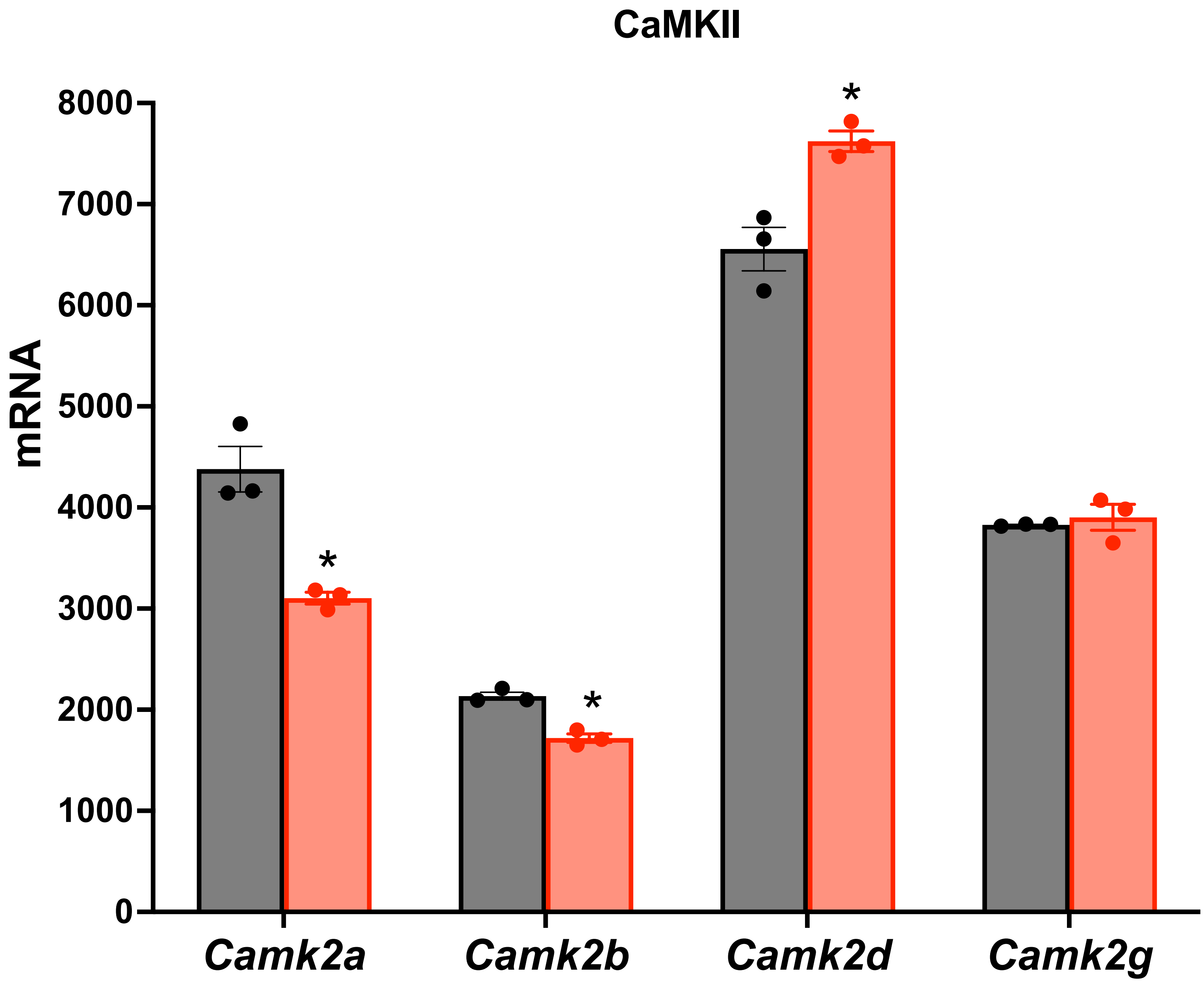

Figure S11. Changes in Ca^{2+}-calmodulin-dependent protein kinase II transcripts in the AV node in HF. Mean (+SEM) expression (and individual data points) for control (black bars) and HF (red bars) mice shown. *P<0.05.

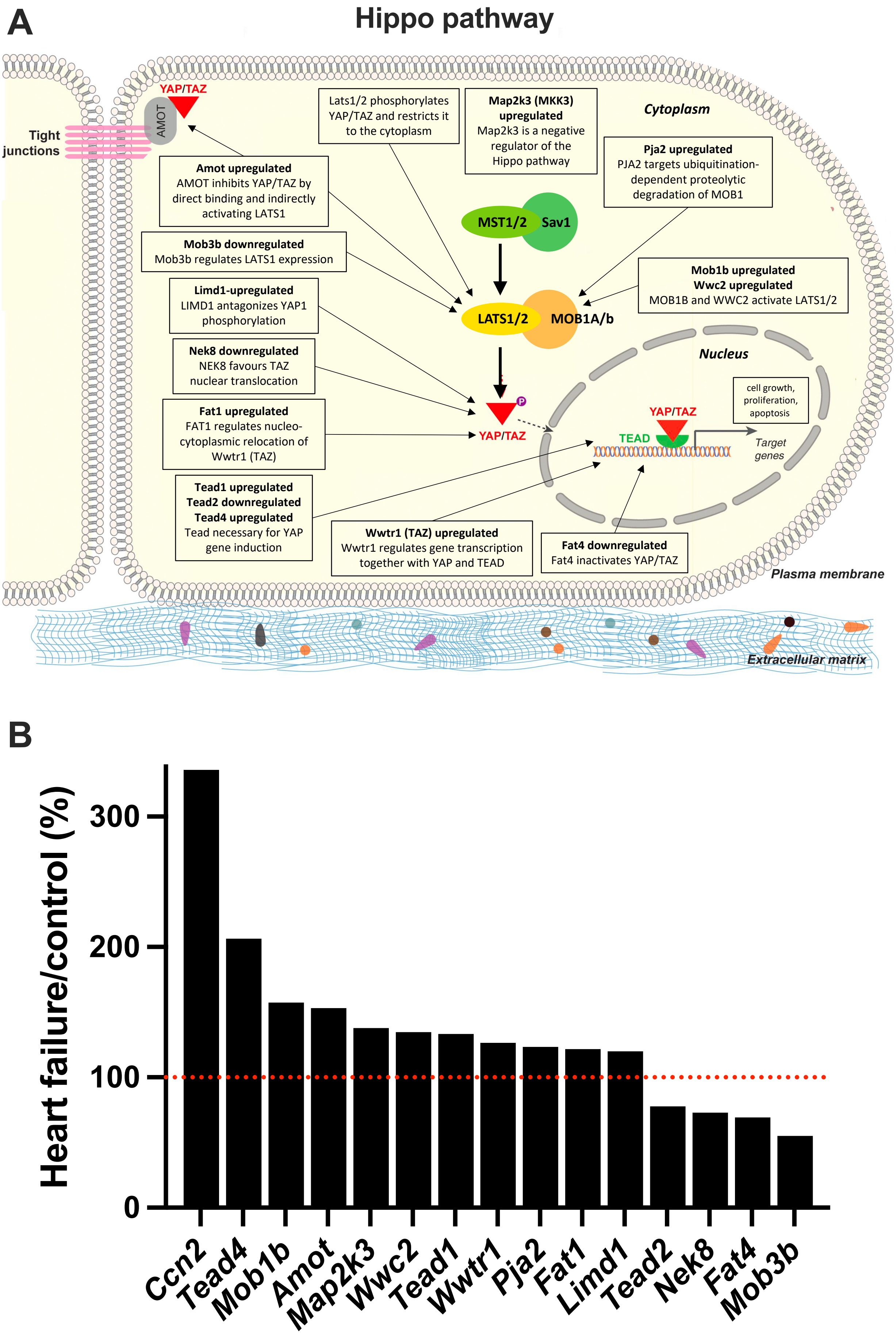

Figure S12. Changes in Hippo pathway transcripts in the AV node in HF. A, schematic diagram of the Hippo pathway and changes in HF. **B**, significant changes in the expression of Hippo pathway transcripts. Expression in HF mice is shown as a percentage of that in control mice. Red dotted line corresponds to 100%.

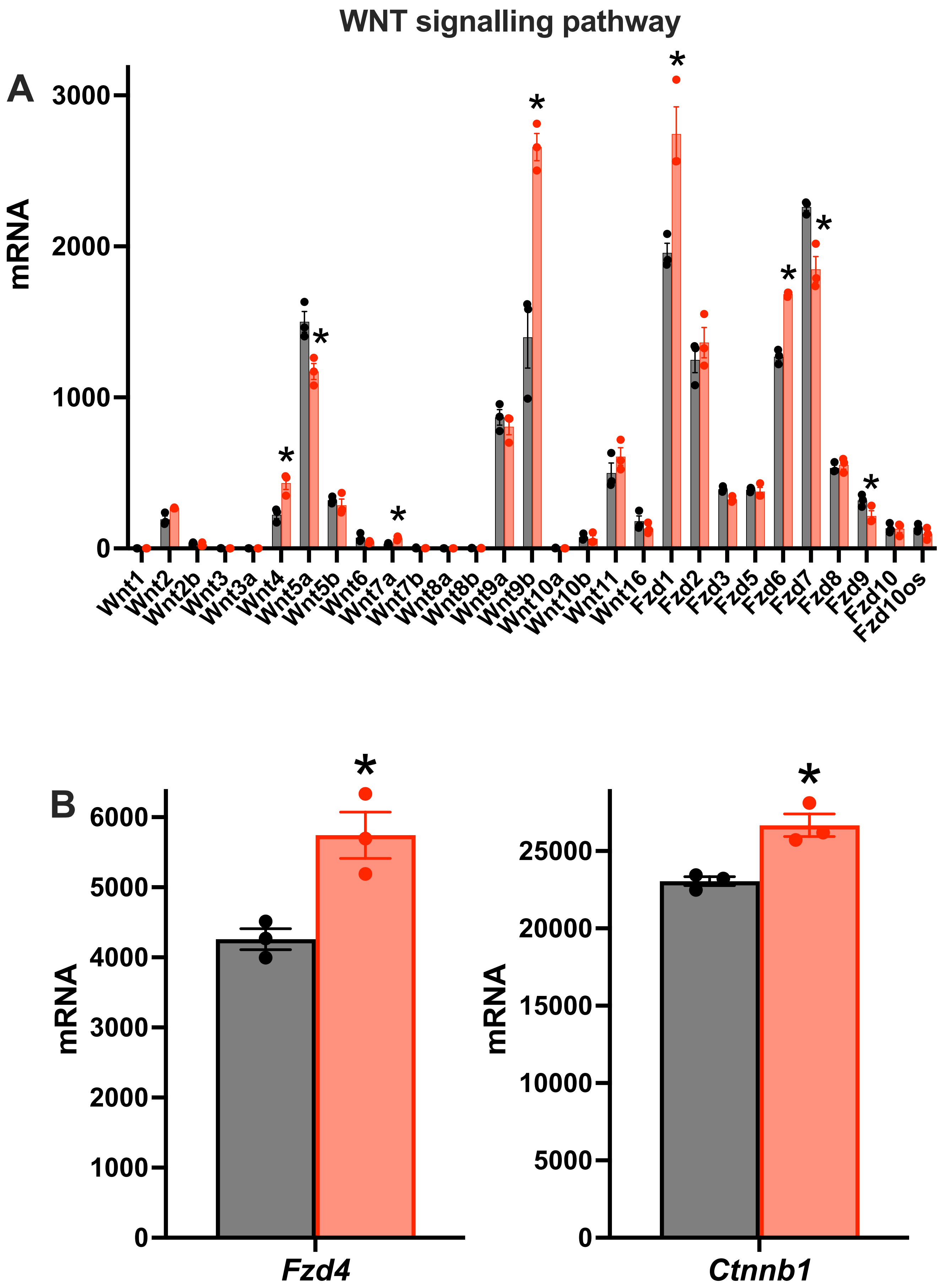

Figure S13. Changes in WNT pathway transcripts in the AV node in HF. A and **B**, mean (+SEM) expression (and individual data points) for lowly (**A**) and highly (**B**) expressed transcripts in control (black bars) and HF (red bars) mice. *P<0.05.

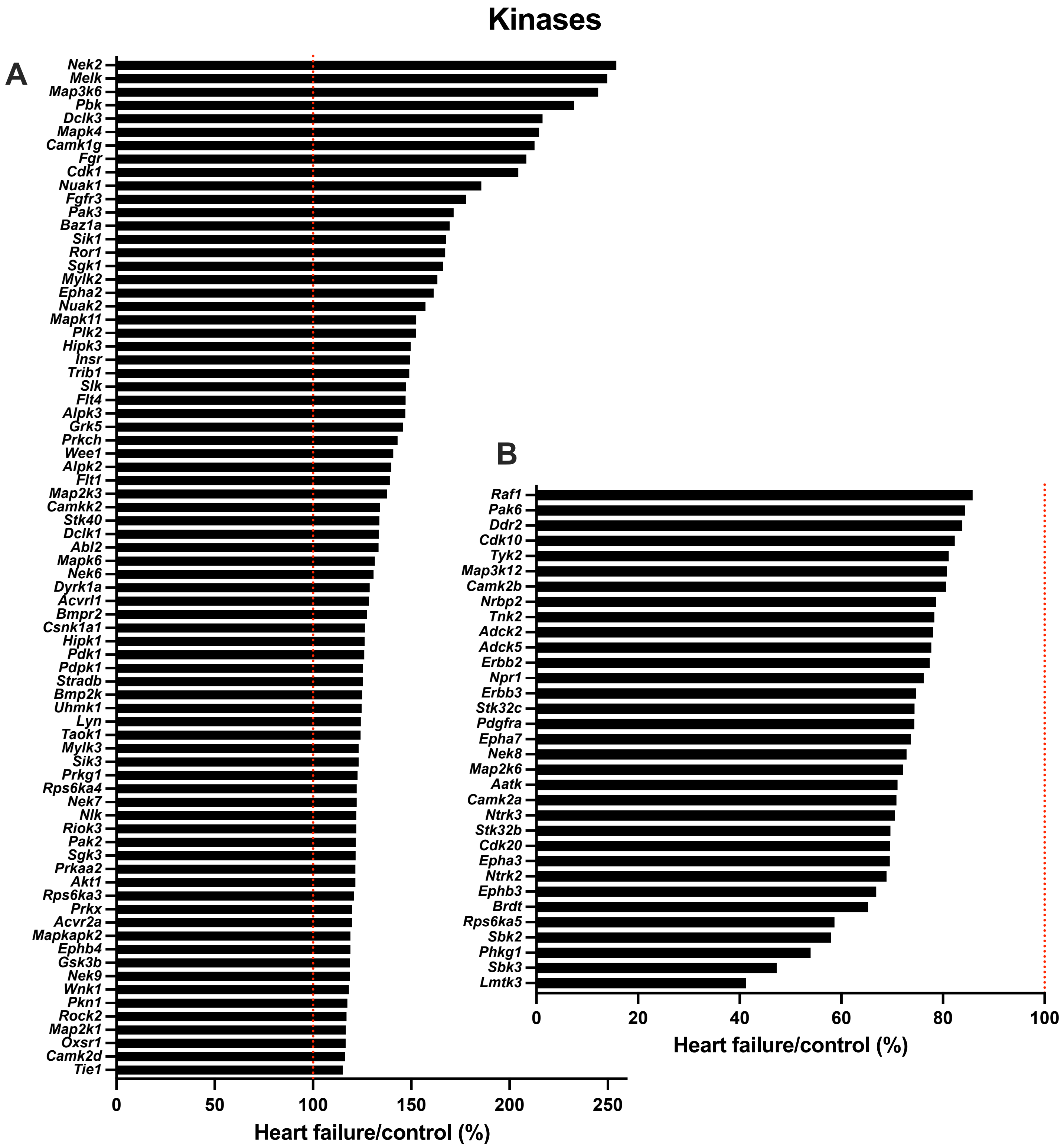

Figure S14. Changes in kinase transcripts in the AV node in HF. A, kinase transcripts significantly upregulated in HF. **B**, kinase transcripts significantly downregulated in HF. Expression in HF mice is shown as a percentage of that in control mice. Red dotted lines correspond to 100%.

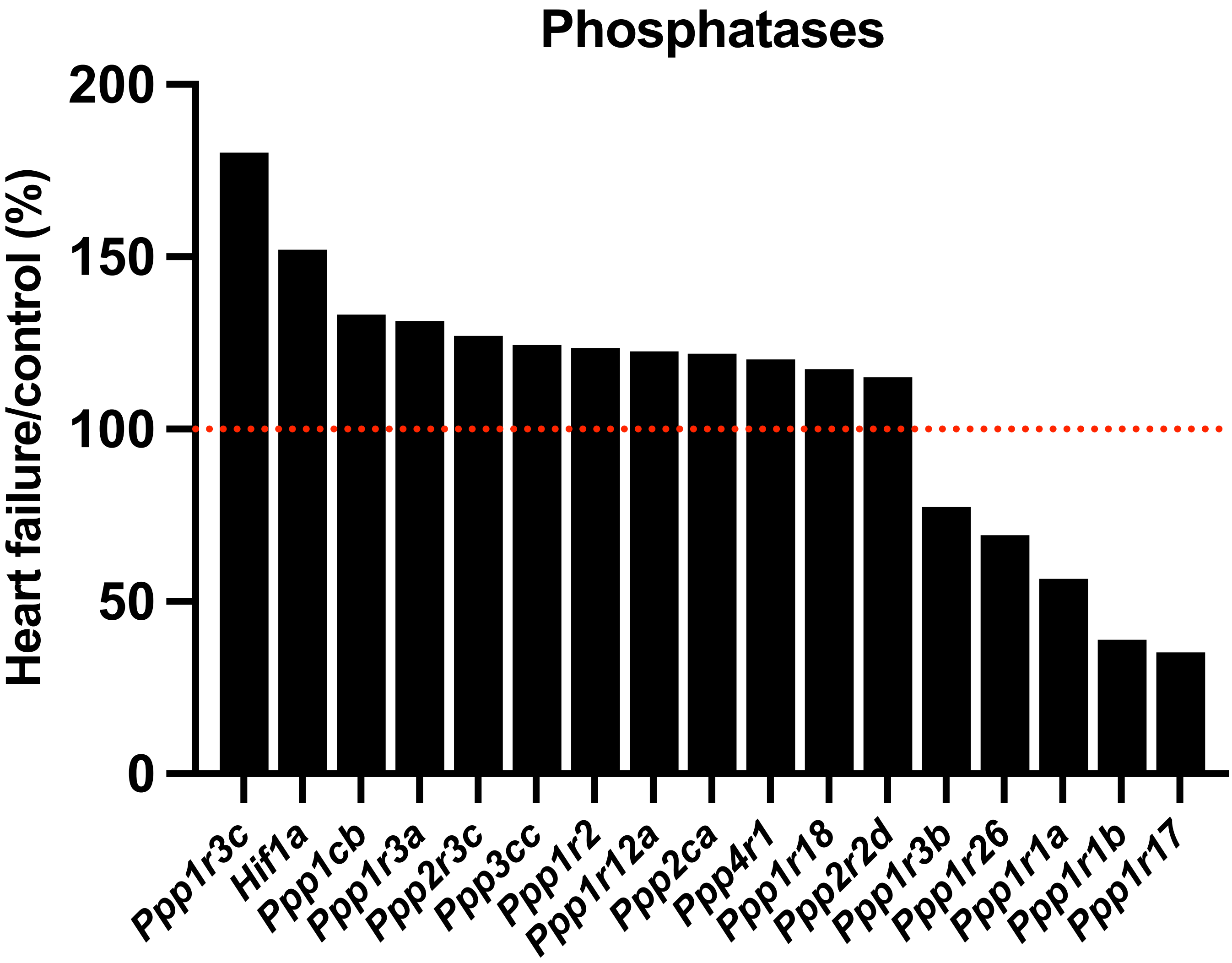

Figure S15. Changes in phosphatase transcripts in the AV node in HF. Significant changes in the expression of phosphatase transcripts and a phosphatase-related transcript shown. Expression in HF mice is shown as a percentage of that in control mice. Red dotted line corresponds to 100%.

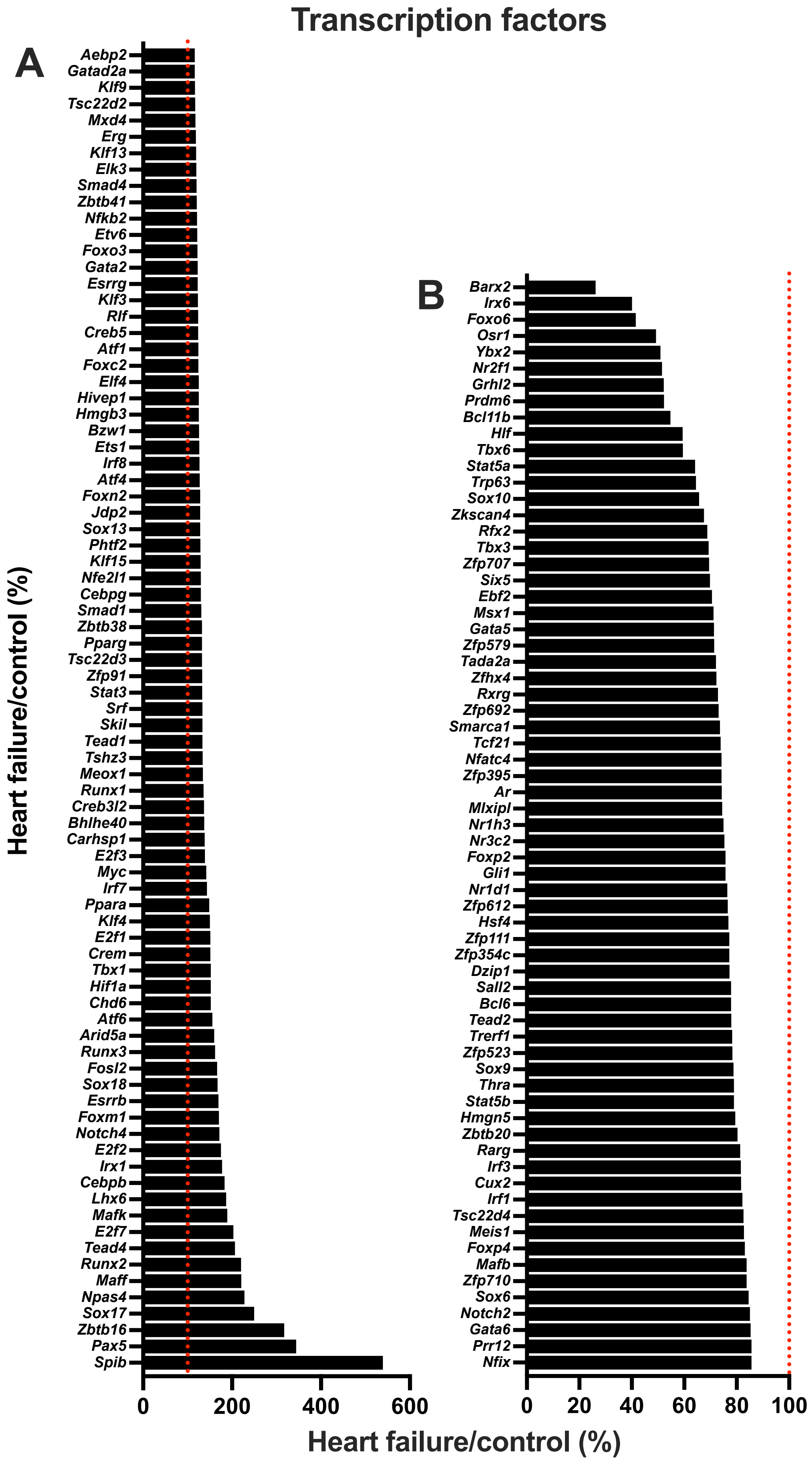

Figure S16. Changes in transcription factor transcripts in the AV node in HF. A, transcription factor transcripts significantly upregulated in HF. **B**, transcription factor transcripts significantly downregulated in HF. Expression in HF mice is shown as a percentage of that in control mice. Red dotted lines correspond to 100%.

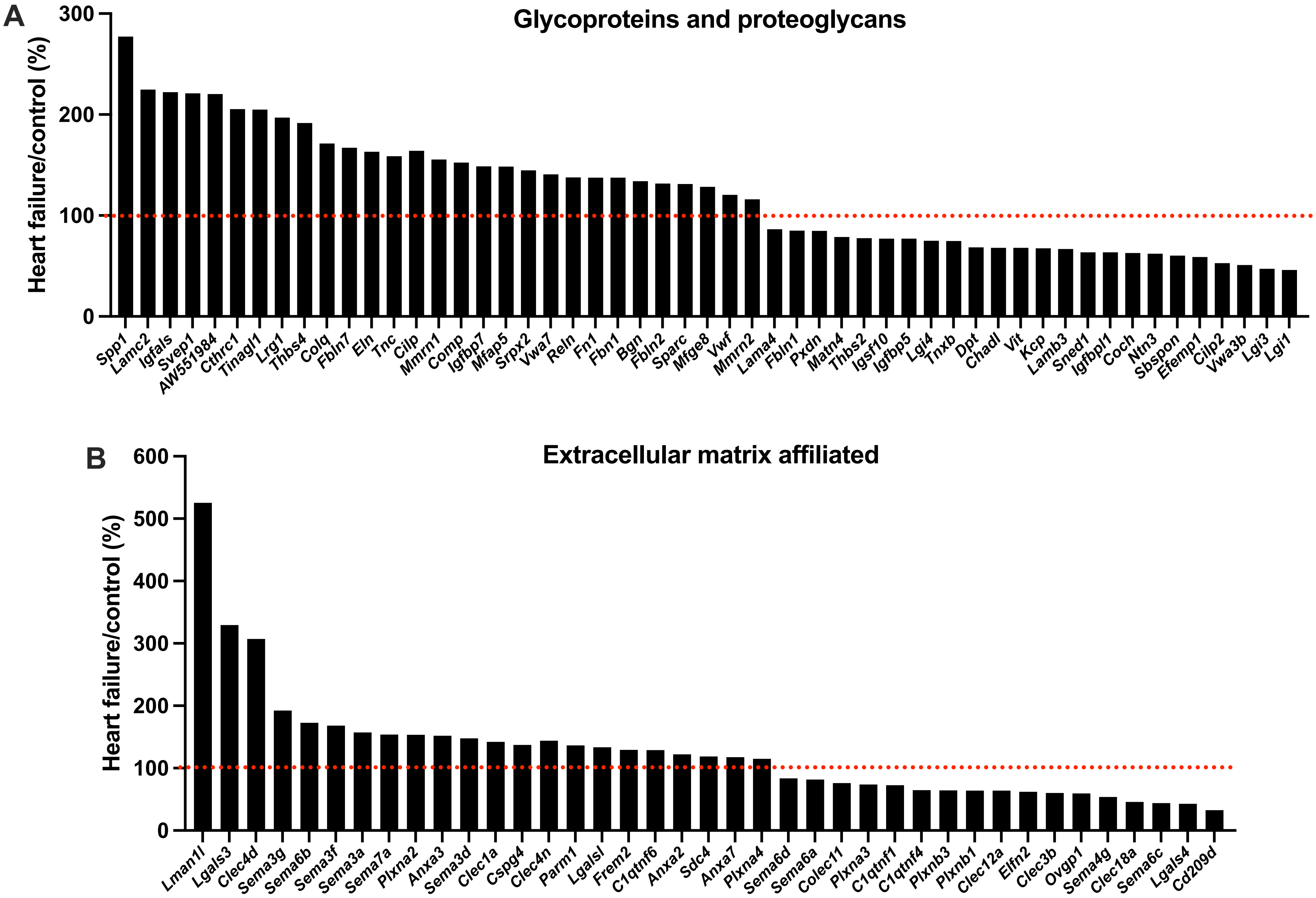

Figure S17. Significant changes in Matrisome transcripts responsible for the extracellular matrix in the AV node in HF – glycoprotein and proteoglycan (A) and extracellular matrix affiliated (B) transcripts. Expression in HF mice is shown as a percentage of that in control mice. Red dotted lines correspond to 100%.

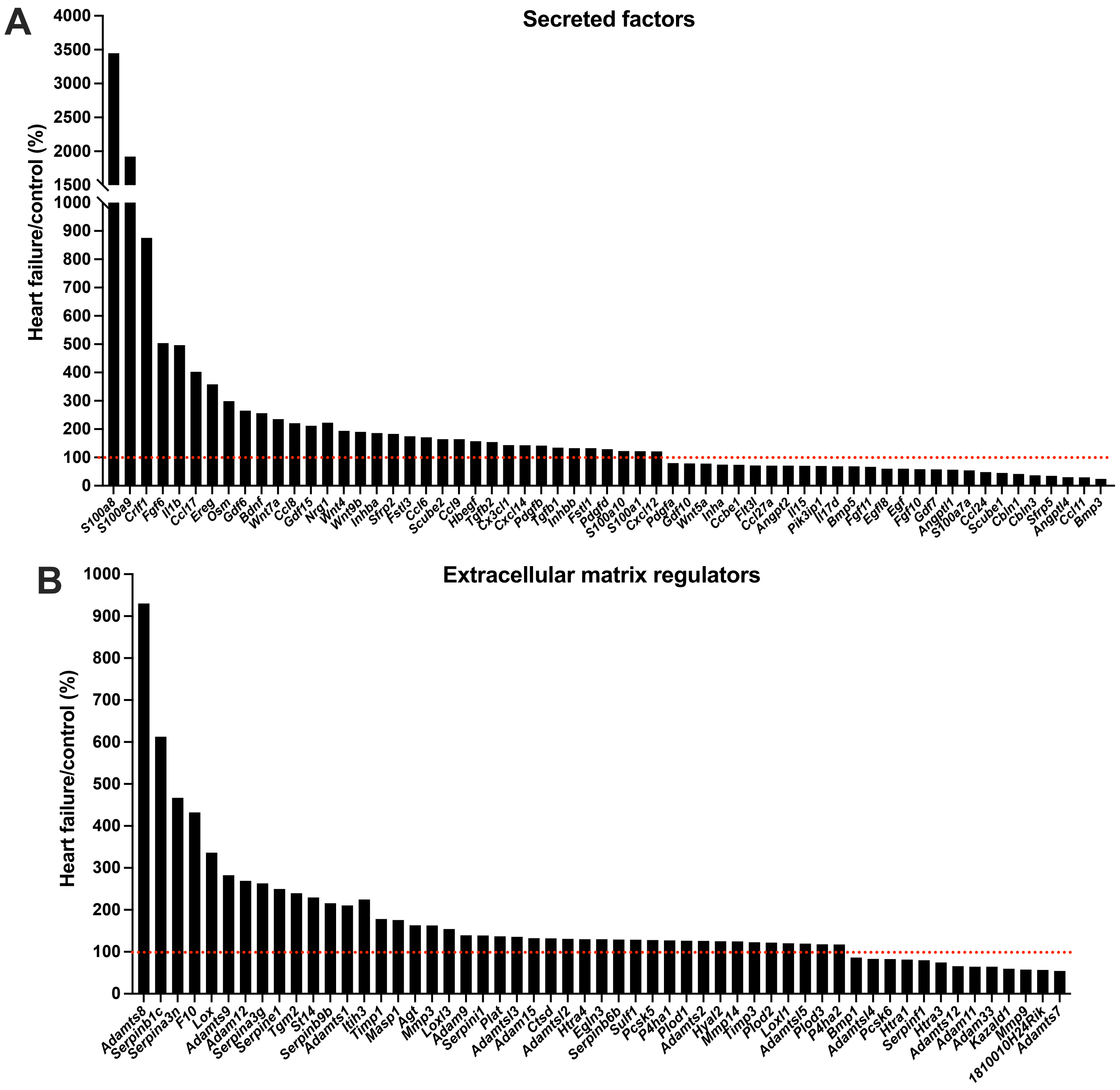

Figure S18. Significant changes in Matrisome transcripts responsible for the extracellular matrix in the AV node in HF – secreted factor (A) and regulator (B) transcripts. Expression in HF mice is shown as a percentage of that in control mice. Red dotted lines correspond to 100%.

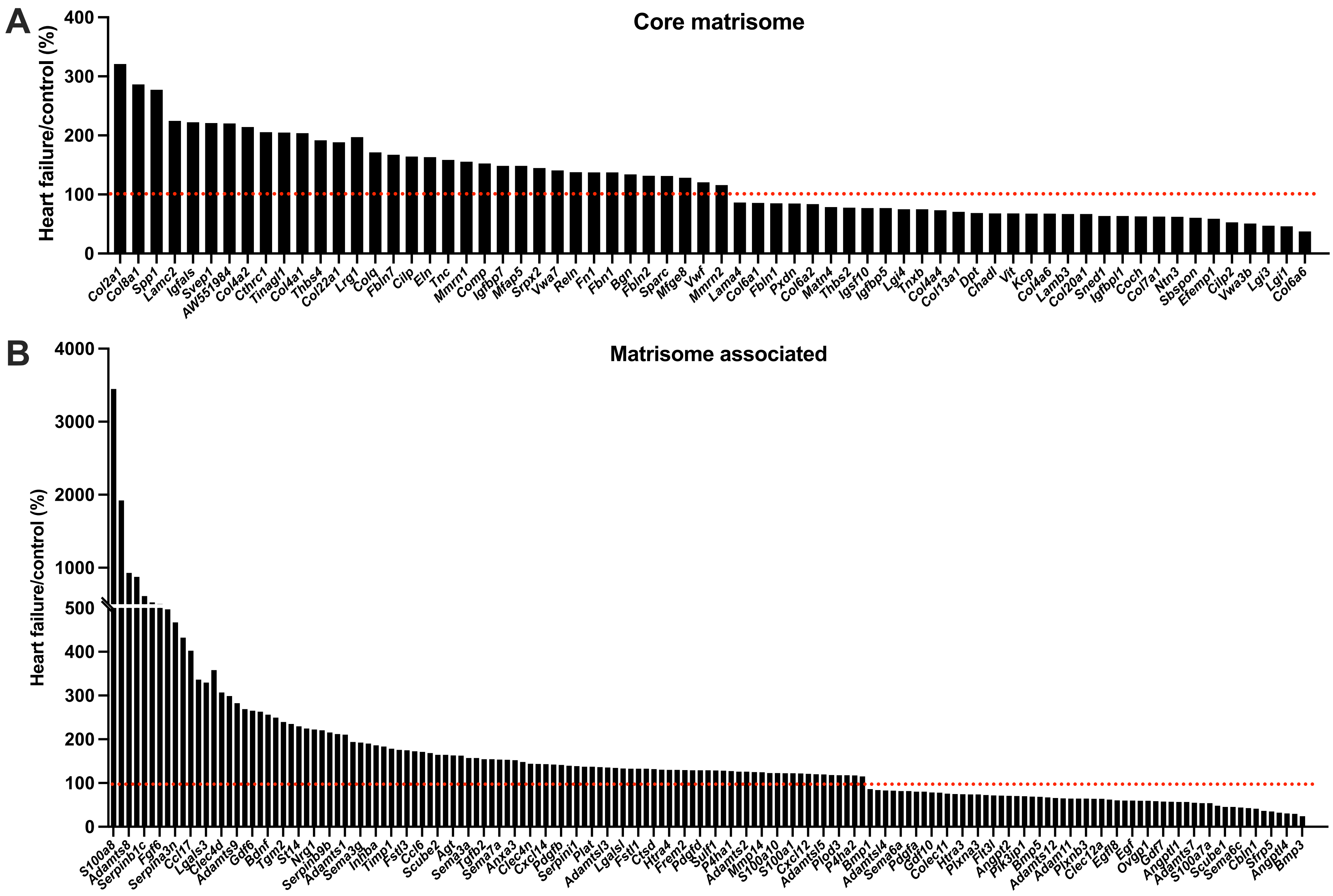

Figure S19. Significant changes in Matrisome transcripts responsible for the extracellular matrix in the AV node in HF – core Matrisome (A) and Matrisome associated (B) transcripts in the AV node in HF. Expression in HF mice is shown as a percentage of that in control mice. Red dotted lines correspond to 100%.

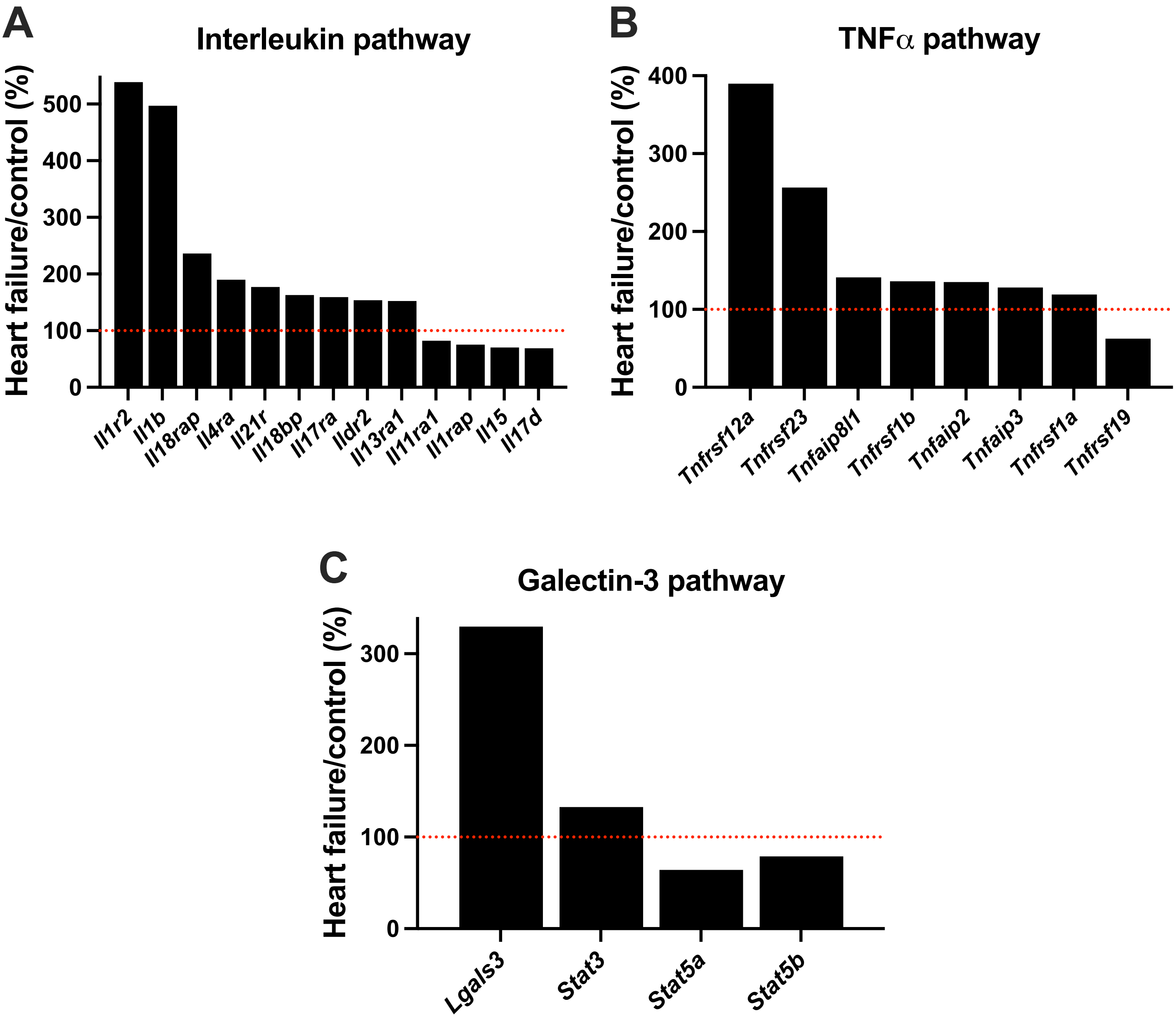

Figure S20. Significant changes in cytokine transcripts in the AV node in HF. Changes in interleukin pathway (**A**), TNFα (**B**) and galectin-3 pathway (**C**) transcripts shown. Expression in HF mice is shown as a percentage of that in control mice. Red dotted lines correspond to 100%.

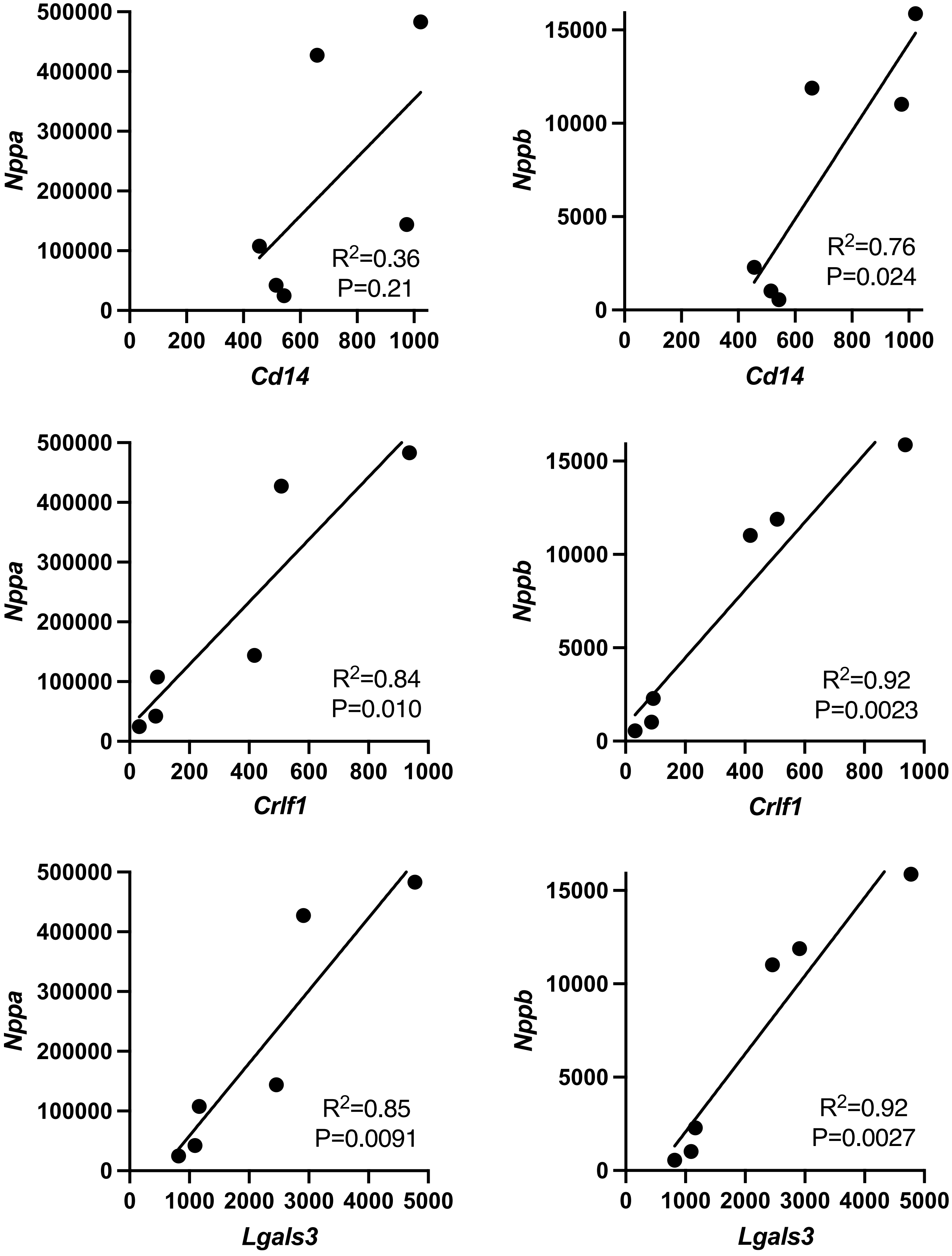

Figure S21. Relationship between two HF markers (*Nppa* and *Nppb*) and a macrophage marker (*Cd14*), a cytokine transcript (*Crlf1*), and a transcript for a cytokine-like molecule known to be associated with HF (*Lgals3*). Each point corresponds to one of six pooled samples (three control and three HF samples). Each pooled sample is made up of AV node biopsies from three mice. The data have been fitted with a straight line and the R^2 value and the P value (of a slope of zero) are shown. In each case the control samples are clustered near the origin.

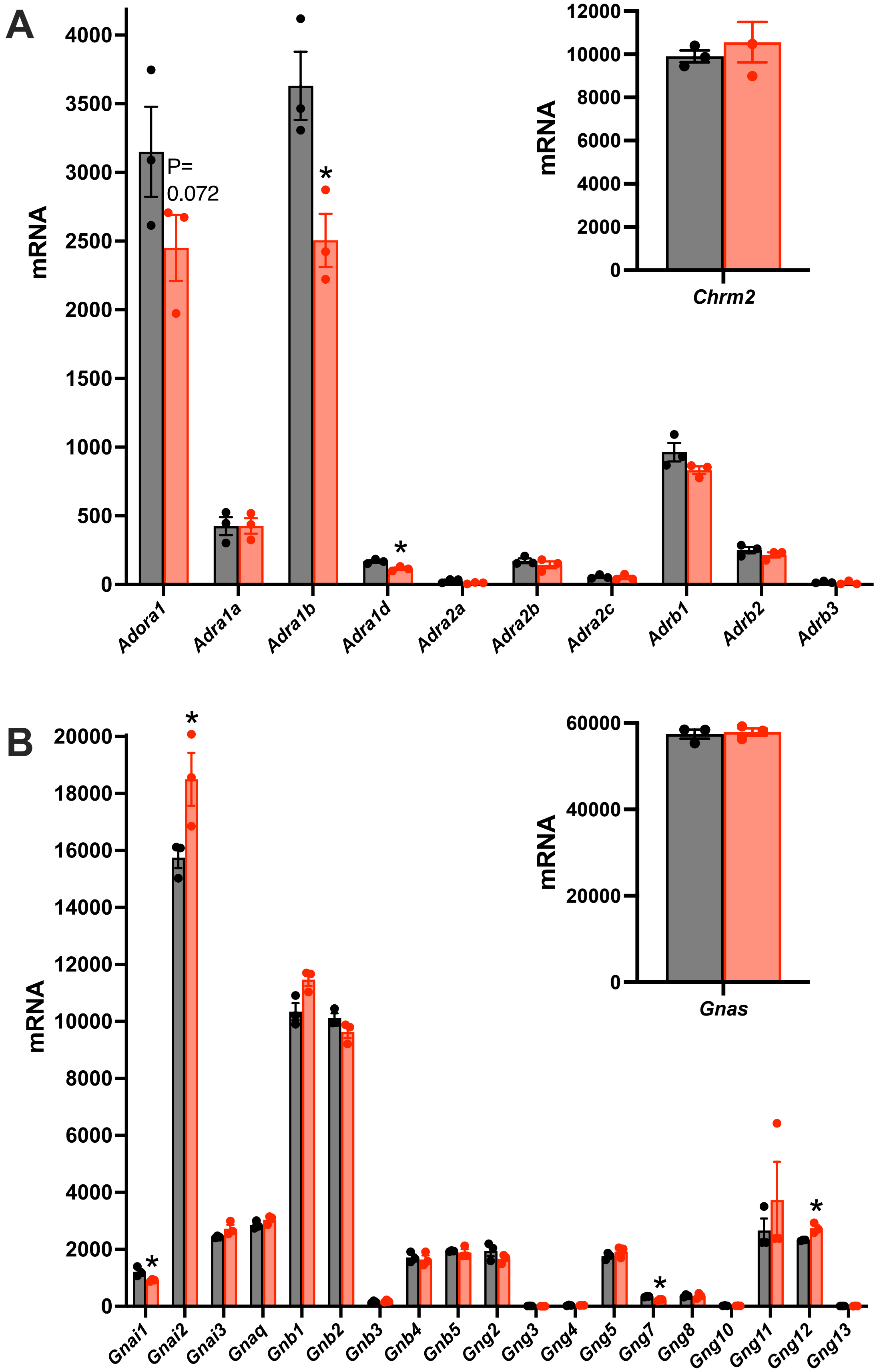

Figure S22. Changes in receptor transcripts in the AV node in HF. A, mean (+SEM) expression (and individual data points) for adenosine, adrenergic and muscarinic (inset) receptor transcripts in control (black bars) and HF (red bars) mice. **B**, mean (+SEM) expression (and individual data points) for G protein subunit transcripts in control (black bars) and HF (red bars) mice; data for *Gnas* shown in the inset. *P<0.05.

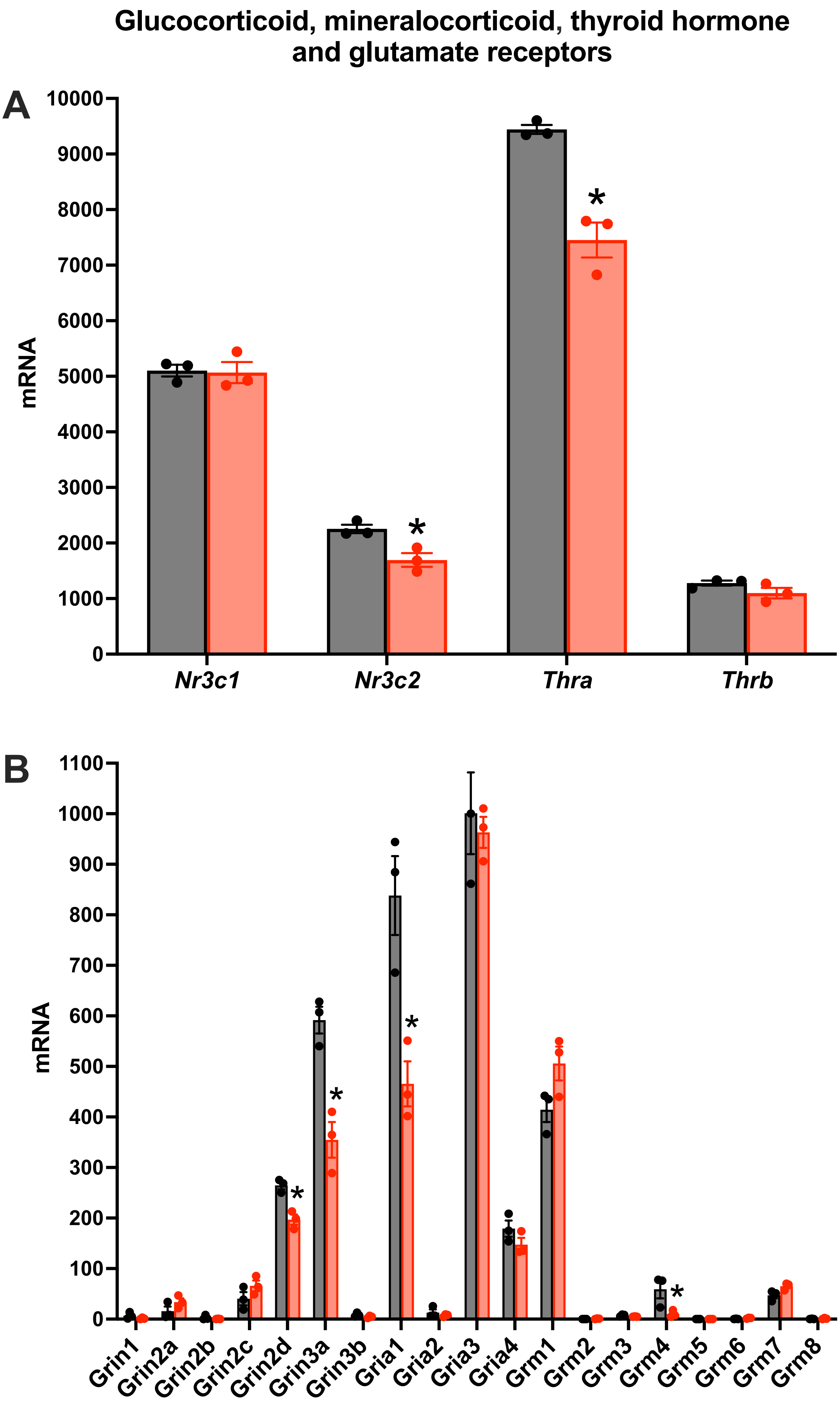

Figure S23. Changes in glucocorticoid (cortisol), mineralocorticoid (aldosterone) and thyroid hormone receptors (A), and glutamate receptors (B) in the AV node in HF. Mean (+SEM) expression (and individual data points) for control (black bars) and HF (red bars) mice shown. *P<0.05.

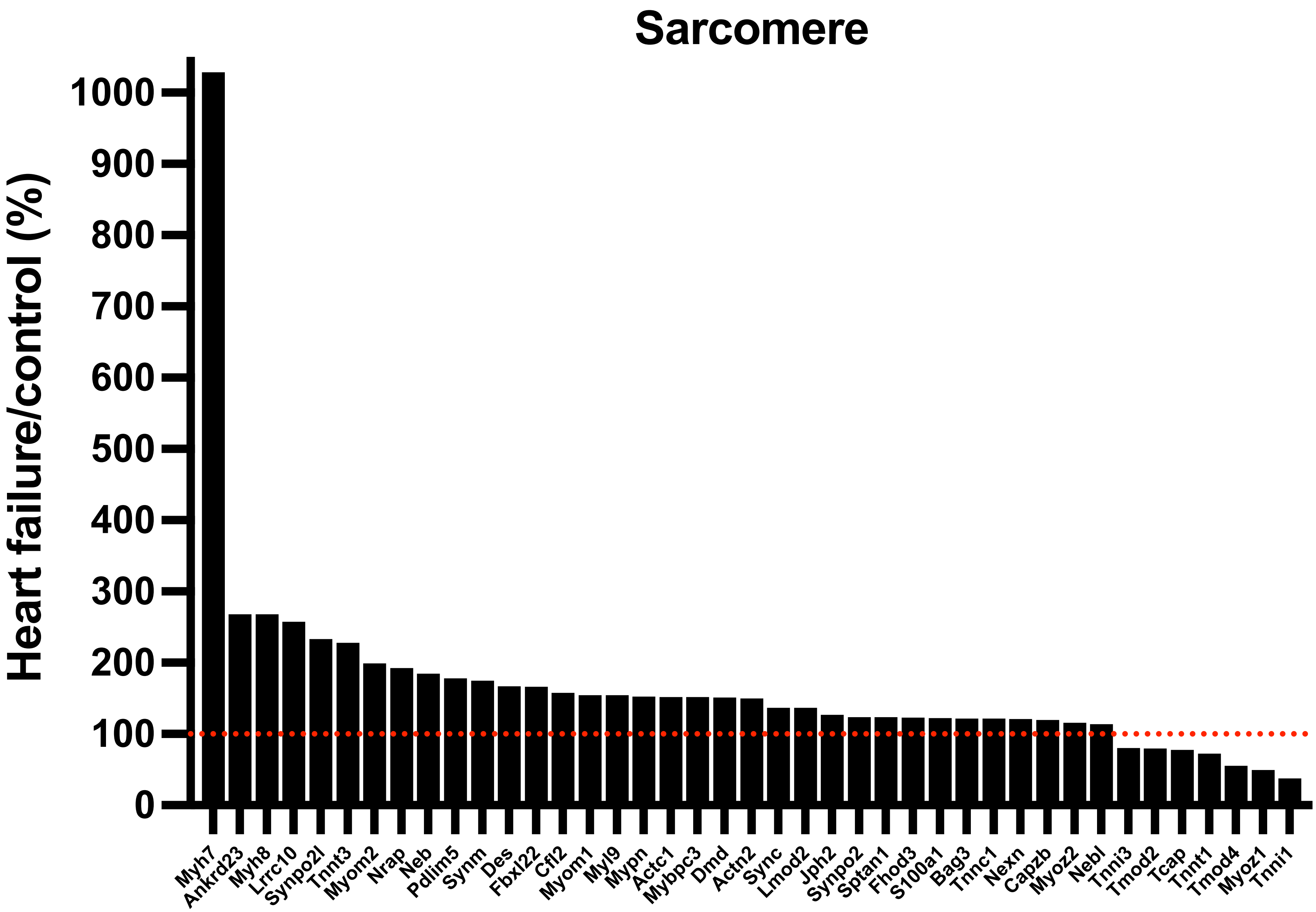

Figure S24. Significant changes in sarcomeric transcripts in the AV node in HF. Expression of transcripts in HF mice shown as a percentage of that in control mice. Red dotted line corresponds to 100%.

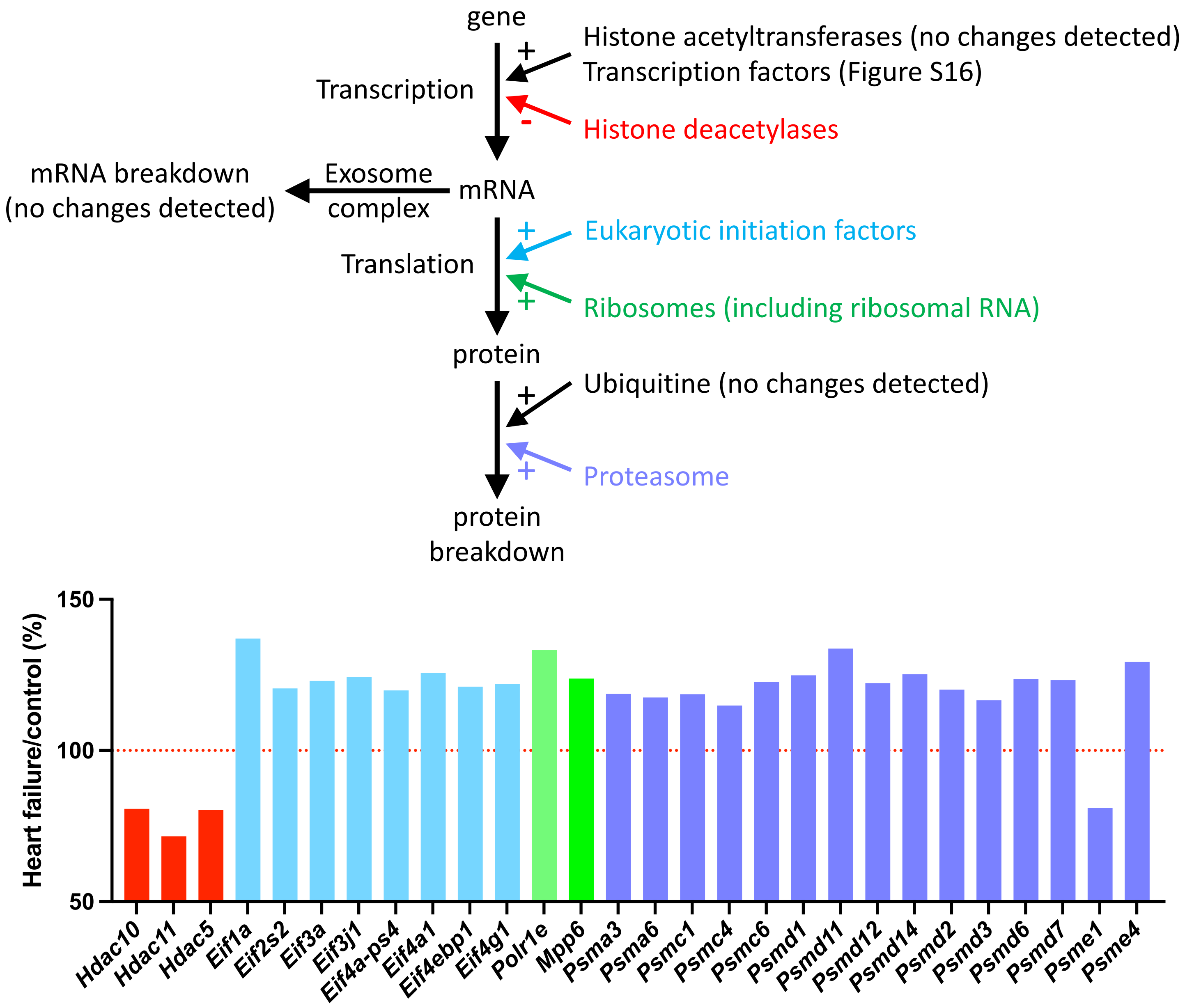

Figure S25. Transcription, translation, and mRNA transcript and protein breakdown. Top, schematic diagram of the cycle of transcription, translation, and RNA and protein degradation (modified from Wang *et al.*, 2021b). Bottom, expression of transcripts involved in transcription, translation, and mRNA transcript and protein breakdown in HF (as a percentage of that in control). Colours correspond to the different parts of the pathway above. Red dotted line corresponds to 100%.
